# Unveiling the role of chromosome structure morphology on gene function through chromosome conformation analysis

**DOI:** 10.1186/s13059-024-03472-8

**Published:** 2025-02-13

**Authors:** Yuxiang Zhan, Asli Yildirim, Lorenzo Boninsegna, Frank Alber

**Affiliations:** 1 Department of Microbiology, Immunology and Molecular Genetics, University of California Los Angeles, Los Angeles, CA 90095 USA; 2 Institute for Quantitative and Computational Biosciences, University of California Los Angeles, Los Angeles, CA 90095 USA; 3 Department of Quantitative and Computational Biology, University of Southern California, Los Angeles, CA 90089 USA

**Keywords:** Chromosome structure, Genomics, Conformational states, Gene transcription, Dimension reduction, Cell-to-cell heterogeneity, Genome structure variation

## Abstract

Single-cell chromosome conformations vary significantly among individual cells. We introduce a two-step dimensionality reduction method for density-based, unsupervised clustering of single-cell 3D chromosome structures from simulations or multiplexed 3D-FISH imaging. Our method clusters up to half of all structures into 5–12 prevalent conformational states per chromosome. These states are distinguished by subdivisions into chromosome territory domains, whose boundary locations influence subnuclear positions and speckle associations of certain genes and establish long-range structural variations of more than 10 Mb. Territory domain boundaries are found at few sequence locations, shared among cell types and often situated at syntenic breakpoints.

**Supplementary Information:**

The online version contains supplementary material available at 10.1186/s13059-024-03472-8.

## Background

With the advent of single-cell super resolution imaging [1–5], multiplexed FISH imaging [6–11], single-cell genomics experiments [12–16], and data-driven genome modeling [17–28], it is now possible to analyze 3D structures of chromosomes and entire genomes at the single cell level. Chromatin loops, topological associated domains (TADs), and patterns of chromatin compartmentalization are readily detected in ensemble averaged Hi-C data [29–32] but are very dynamic in nature and subsequently show large stochastic variations at the single cell level [2, 33]. For instance, chromatin loops, detected at specific locations in ensemble Hi-C of mammalian cells, are likely present only in 3 to 6.5% of cells at any given time [34] and TAD domain boundaries are rarely observed at the ensemble average position but are rather stochastically distributed, because of dynamic loop extrusion processes [2, 19, 35, 36]. Thus, detailed analysis of single-cell chromosome structures are only meaningful when considering the entirety of structural variability observed in a cell population [37–41]. In contrast to chromatin loops and TADs, there has been comparatively less research into the structural variations of entire chromosomes between cells, and its role in the cell-to-cell variability of long-range chromatin interactions. Recent evidence from multiplexed FISH imaging [6, 9, 37] and single-cell Hi-C experiments [13, 14, 42, 43] suggests large-scale structural variations of chromosome morphologies between single cells. For instance, single chromosome clustering identified five subpopulations of conformations for chromosome-V of *C. elegans* embryos, which revealed at a coarse resolution the relative spatial positions of 22 TADs to each other [40]. Other studies focused mostly on smaller fragments of chromosomes [37, 41]. However, to our knowledge no method exists to study at higher-resolution chromosome conformational states within the context of the subnuclear environment. Moreover, polymer simulations have observed transitions between open and more globular conformations of chromosomal regions, as for instance a dumbbell-like conformation for a 2-Mb segment of human chromosome 2 [37, 44].

Variations in chromosome conformations could in principle affect a gene’s location within the nuclear topography, defined as the subnuclear location with respect to nuclear bodies, radial position and nuclear compartments and thus its exposure to functional compartments and nuclear bodies, which have been shown to be of relevance for gene function [45]. For instance, gene transcription can be heightened in the close vicinity of nuclear speckles [46–48]. However, up to this point chromosome structural diversity was studied mostly on chromosomal regions of individual loci rather than entire chromosomes and without considering a chromosome’s orientation within the nuclear topography of single cells. Furthermore, it remains understudied whether large-scale variations in the spatial chromosome morphology play a role in regulating gene function and thus be a source for transcriptional heterogeneity between single cells.

In this study, we tackle these issues by identifying prevalent subpopulations of 3D chromosome structures for entire human chromosomes in the context of the nuclear topography of single cells. We also examine how these structural variations affect the functional microenvironment of genomic regions within the nucleus. We address several important questions, which are non-trivial due to the stochastic, fluid nature of 3D chromosome structures. First, can the structures of entire human chromosomes from different single cells be classified into prevalent structural states that define distinct chromosome morphologies? Second, do chromosome morphologies of prevalent structural states influence gene functions? To address these questions, we first introduce an approach for unsupervised clustering of an ensemble of single-cell chromosome structures, extracted from 3D structures of entire human genomes, either taken from chromatin tracing experiments (for instance, multiplexed DNA-MERFISH imaging [6] and SeqFISH + [9]) or genome structure models generated with our data-driven IGM (Integrated Genome modeling) approach [17]. Because chromosome structures are dynamic in nature, classifying them based on 3D coordinates is challenging. This is because certain functionally unrelated regions can show substantial variability in their relative positions, potentially obscuring the detection of functionally relevant structural similarities among other chromosomal regions within a subpopulation of structures. Our approach effectively addresses this challenge by formulating the task of clustering individual chromosome structures as a problem of identifying peaks in a density distribution within a reduced 2-dimensional space. In this space, each data point corresponds to a chromosome conformation, and the detection of local maxima in the probability density function determines the positions of densely populated clusters of chromosomes with similar 3D conformations. As a result, our approach determines subpopulations of chromosomes with similar 3D morphology. Unlike other unsupervised clustering methods [37, 40, 41], our approach does not coerce all the conformations into a defined set of clusters. Instead, it rather detects only those morphologies that are substantiated by a sufficient number of similar structures at a given sample size. Furthermore, we examine significantly larger chromosomes from the entire human genome and concentrate our analysis on the connection between chromosome morphology and nuclear topography.

We observed that, depending on the specific chromosome and sample size, up to half of all chromosome structures can be grouped into approximately 5 to 12 major conformational classes. These chromosome morphologies are distinguished by the presence of characteristic chromosome territory subdivisions, which partition the chromosome conformation into one or multiple chromosome territory domains. These domains and the sequence locations of their boundaries play a crucial role in establishing long-range conformational variations between chromosomes. We validated our results with data from multiplex DNA-MERFISH imaging [6] along with single-cell sci-HiC experiments [13]. Noticeably, territory domain boundaries are not randomly distributed along the sequence; instead they are found only at a few specific locations in the sequence of a chromosome. These boundaries often mark a transition between gene-poor and gene-dense regions and about half of these boundaries are also located at syntenic breakpoints. Furthermore, most territory domain boundaries can be found in chromosomes of different cell types (e.g., GM12878, H1-hESC and HFFc6).

Interestingly, different chromosome morphologies alter the preferential positions of specific chromosomal regions within the nucleus, in particular in terms of their radial positions and distances from nuclear speckles. Short distances from nuclear speckles have previously been linked to increased gene transcription activity [19, 47–49].

Using spatial transcription data from RNA- and DNA-MERFISH experiments [6], we found that certain genes exhibit increased transcription when the chromosome adopts a specific morphology that favors its position in a more interior location within the nucleus. Therefore, our observations suggest that prevalent chromosome morphologies may contribute to modulating the functional properties of chromosomal regions, which could, at least in part, account for the cell-to-cell heterogeneity of gene expression among cells. Our method provides an important approach to study chromosome conformational variations and reveal links between chromosome morphologies and gene functions.

## Results

### Structure generation

We first apply our approach to an ensemble of diploid 3D genome structures that were generated at 200 kb resolution from ensemble Hi-C data [17, 19, 31]. Our population-based 3D genome modeling method (IGM—Integrative Genome Modeling, Methods) [17, 19, 50, 51] generates a large sampling of 10,000 diploid single-cell 3D genome structures per cell type, which, as a whole, reproduce the input Hi-C data and predict with high accuracy other orthogonal experimental data [17, 19]; namely, average radial positions of genomic regions from GPseq experiments [52], mean speckle distances from SON TSA-seq [49], mean distances and contact frequencies to the nuclear periphery from lamin B1 TSA-seq [49] and lamin B1 pA-DamID [53]. Moreover, predicted chromosome structures are in good agreement with single-cell 3D chromosome conformations from multiplex DNA-MERFISH experiments [6, 17, 19], and also reproduce with high correlation speckle and lamina association frequencies of genomic regions from DNA MERFISH [17, 19]. We first focus our analysis on genome structure models from lymphoblastoid cells (GM12878) from previously published work [19], human fibroblast (HFFc6) [17] and human embryonic stem cells (H1ESC)), and later classify genome structures from DNA-MERFISH experiments [6].

### Approach

**Fig. 1:**
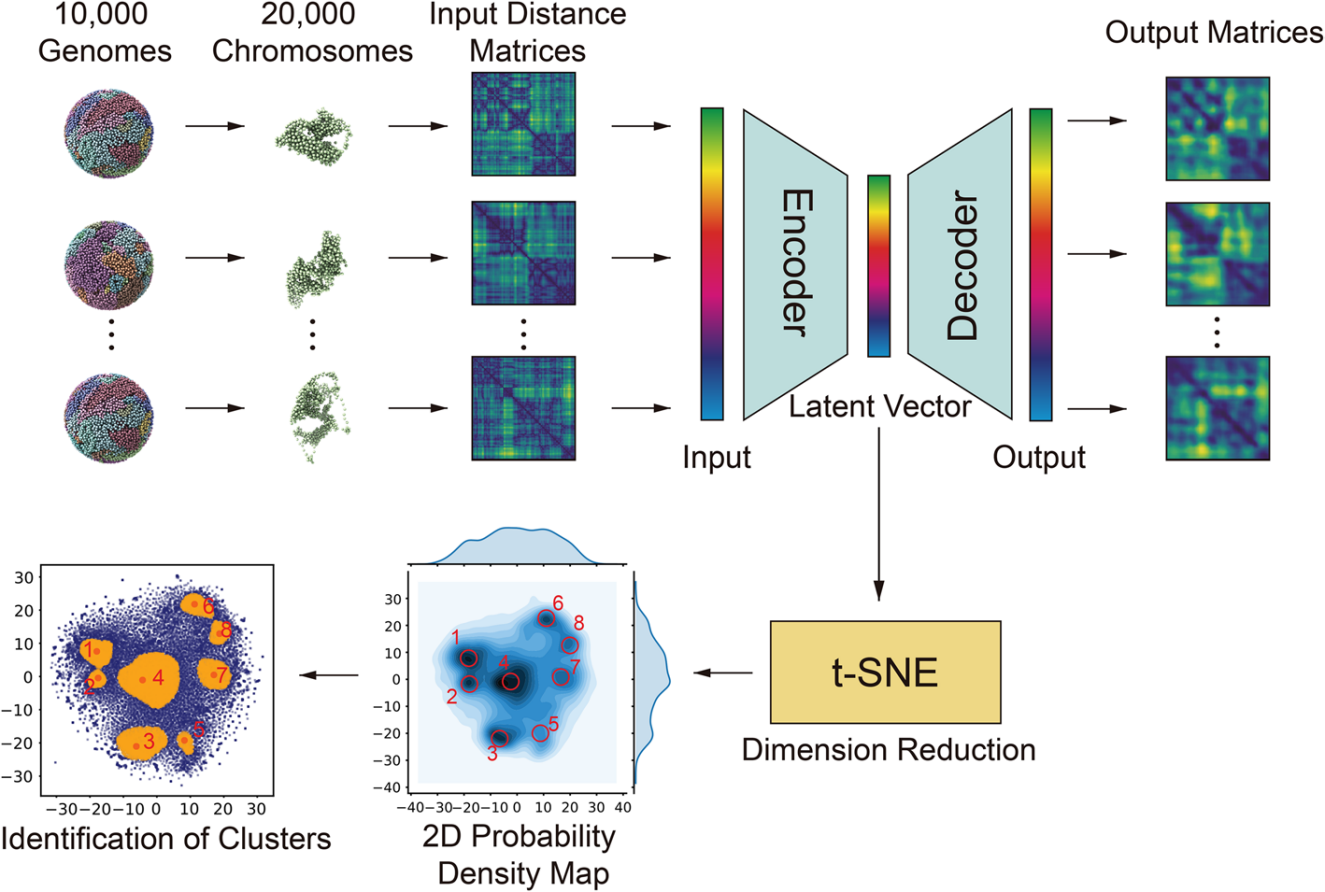
Overview of the two-step dimension reduction. Every chromosome structure from each cell is represented by an input distance matrix, which is constructed by calculating pairwise Euclidean distances between each pair of loci in the chromosome structure. After preprocessing, the matrix is then used as the input of the autoencoder. After minimizing the loss between input matrices and output matrices, the latent vectors are then embedded by t-SNE [54] to obtain a distribution of all chromosome structures in 2D space. The resulting distribution is further used for peak detection and identification of clusters of chromosome structures (Methods)

To classify chromosome morphologies, we first isolate individual chromosome copies from each whole genome structure in the cell population, resulting in a total of 20,000 chromosome structures for each autosome (Fig. 1). For each of those structures, we then construct a normalized distance matrix, which then serves as input into a dimension reduction and clustering scheme (Fig. 1). We use a two-step dimension reduction approach to cluster chromosome structures based on their distance matrices into morphological states (Methods). Specifically, each normalized distance matrix is envisioned as a 2D image. Our two-step process combines a convolutional autoencoder (consisting of an encoder and a decoder module) with a dimensionality reduction step using t-distributed stochastic neighbor embedding (t-SNE) [54] (Methods). The encoder module reduces a distance matrix to a latent vector that can reconstruct the original matrix by the decoder module. The method reduces the dimensionality, while preserving enough information to reconstruct the original image. To construct a convolutional autoencoder, we use convolutional layers, max pooling layers and up sampling layers, which is frequently used for image embedding and classification (Additional file 1: Fig. S1) (Methods). t-SNE, a method to separate data points in a reduced data space, is then used to project the latent vectors (generated by the autoencoder) onto a lower dimensional space (Fig. 1). Finally, we use a kernel density estimation to calculate a probability density function (pdf) that represents the chromosome conformational space in the t-SNE reduced dimensions. The resulting density probability matrix shows a balanced distribution of local maxima separated by deep valleys (Fig. 1 lower panels), indicating the marked presence of a number of preferred conformational states (Methods). We then determine these local maxima as cluster centers and identify structures associated to each cluster center by watershed segmentation of the probability density distribution (Fig. 1 lower left panel, and Methods). Chromosome structures that fall into the same segmentation are assigned to the same conformational cluster.

**Fig. 2:**
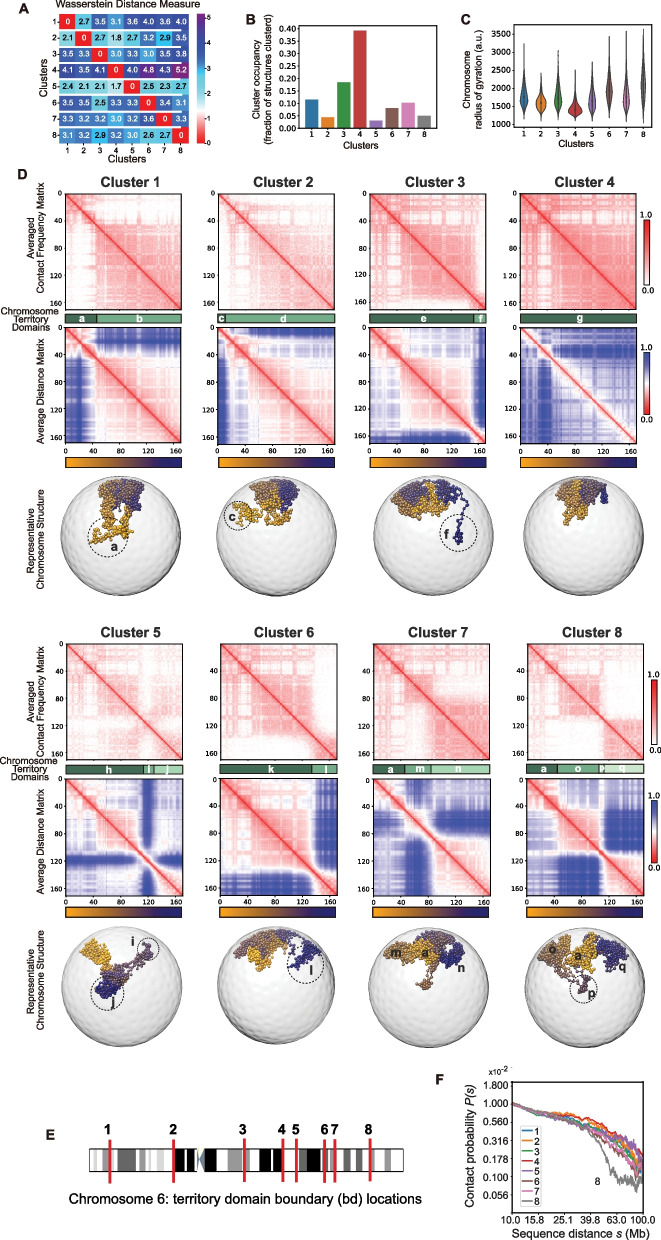
Clustering of chromosome 6 structures reveals dominant chromosome morphologies (Shown are results for GM12878 cells).** A** Pairwise dissimilarity measure between chromosome structures in the 8 detected clusters for chromosome 6. The dissimilarity matrix is calculated by measuring the average Wasserstein distance [56] between all intra-chromosomal distance distributions between two clusters. Each entry represents the log fold ratio between the inter-cluster dissimilarity and the intra-cluster dissimilarity. Positive off-diagonal values indicate larger dissimilarity between structures of different clusters than those within a cluster. **B** The cluster occupancy of the 8 predicted clusters for chromosome 6. The occupancy is defined by the number of chromosome structures in a cluster divided by the total number of structures in all clusters. **C** The distributions of the radius of gyration of all structures in each cluster (Methods). **D** For each cluster the following information is shown (Methods): (top panel) The average contact frequency matrix calculated from all structures in a cluster; (second panel from top) different shade of green indicate the location of chromosome territory domains; (third panel from top). The average distance matrix calculated from all chromosome structures in the cluster; (fourth panel from top) Selected example of a chromosome structure in the cluster. Numbers and circles indicate chromosomal regions of the corresponding chromosome territory domains. The color bar indicates the sequence position of each chromosomal region. We also highlighted several specific genomic regions (regions I, II, and II) below the RadRatio and RGRatio profiles, which are compared and discussed in the text. **E** The genomic positions of the 8 detected domain boundaries on chromosome 6. **F** Contact probabilities versus different ranges of sequence distances for all predicted clusters from the model. We observe similar contact probabilities at smaller scales in all clusters. At larger scales, cluster 4 has the largest contact probabilities while cluster 8 has the smallest contact probabilities

To assess our clustering performance, we employ Silhouette analysis [55]. The Silhouette coefficients of all clustered chromosomes are larger than 0.5 (some reaching up to 0.72), thus confirming that structures in different clusters are well separated in the reduced space (Additional file 1: Table S1). To further quantify the structural similarity of chromosomes within and between clusters, we calculate the average Wasserstein distance metric [56]. The dissimilarity of chromosomes in two different clusters is defined as the average Wasserstein distances of all intra-chromosomal distance distributions, calculated from all chromosomes in each cluster (Fig. 2A). We normalize this dissimilarity measure by the average Wasserstein distance for chromosomes in the same cluster (Methods). We observe that the average Wasserstein dissimilarity measure is always substantially larger (i.e., ~ 2–fourfold) for structures in different clusters, showcasing the structural distinction between chromosomes in the different clusters (Fig. 2A, row-wise comparison). We also found similar results when assessing clusters with other distance measures, including a Euclidean distance measure and Gaussian dissimilarity [37, 57], confirming an overall higher similarity for structures within than between clusters (Methods) (Additional file 1: Fig. S2). (For comparison with other clustering methods see SI (Additional file 1: Fig. S3AB, S4AB).)

### Assessing robustness of clustering method

Because our approach relies on density-based clustering, the cluster detection requires sufficient numbers of similar chromosome structures. We therefore assessed the robustness of our method with a decreasing number of input structures (Additional file 1: Fig. S5). Using 20,000 chromosome structures, we identified eight prevalent conformational clusters for chromosome 6 and between five and 12 clusters for other chromosomes. The same clusters were detected when the number of input structures was reduced to at least 15,000. As the number of input structures decreases further, the number of detected clusters declines (Additional file 1: Fig. S5). For example, with 10,000 input structures, seven of the eight clusters were identified, and with 5000 structures, five of the eight clusters were detected (Additional file 1: Fig. S5). This reduction in cluster detection is due to the difficulty in identifying very low-occupancy clusters with smaller number of input structures, as detecting local density maxima via kernel density estimation requires a sufficient number of structures that are similar to each other. However, clusters with higher occupancy are consistently identified, with nearly identical average distance matrices, underscoring the robustness of our method even with smaller input sizes. Therefore, a larger sample of structures may reveal additional clusters that could go undetected with a smaller sample size (Additional file 1: Fig. S5).

We also tested the robustness of our method with respect to the resolution of the chromosome structures. We downsampled all chromosome structures from 200 kb to 3 Mb resolution, meaning chromatin regions were sampled at intervals of only 3 Mb. Despite the substantially lower structural resolution, we can still identify six out of eight clusters detected in our original 200 kb resolution chromosome structures, with very similar contact patterns (Additional file 1: Fig. S6).

Finally, we also generated a negative control consisting of 20,000 genome structures modeled as random self-avoiding chromosome homopolymers (without Hi-C restraints) constrained only by nuclear volume. As anticipated, the negative control failed to produce our observed clusters (Additional file 1: Fig. S6). As a positive control, we created several datasets consisting of chromosome structures derived from average cluster distance matrices, with varying levels of random noise sampled from a Gaussian distribution (standard deviation ranging from 0.1 to 0.8). Our method successfully separated all data points in the conformation space and accurately clustered all structures from each positive control dataset into their correct clusters, regardless of the noise levels (Additional file 1: Fig. S7).

### A large fraction of chromosomes can be clustered into prevalent structure morphologies

Depending on the specific chromosome, about 25–50% of all chromosome structures can be clustered into 5 to 12 prevalent morphology states (Additional file 1: Table S1, Fig. S8). For instance, about 40% of all chromosome 6 structures and about 50% of chromosome 22 structures can be clustered into 8 and 11 dominant conformational states, respectively (Fig. 1, lower left panel). The number of structures in each cluster varies (Fig. 2B). For chromosome 6, cluster 4 has the highest occupancy containing ~ 40% of all clustered structures, while all other clusters have an occupancy of less than 20% (Fig. 2B). Unclustered structures located midway between two cluster centers have conformations that can be considered as intermediates between the structures in both clusters and have relatively low copy numbers in the population (Additional file 1: Fig. S9). As expected, the overall shape of chromosomes varies between clusters. For instance, cluster 8 of chromosome 6 contains structures with the most extended conformations, having 50% larger average radius of gyration than chromosomes in cluster 4, which shows the lowest average radius of gyration and highest uniformity in shape (Fig. 2C). Chromosome clusters with large average radius of gyration typically show the largest cell-to-cell variations in their shape within the cluster (e.g., clusters 6, 8).

### Chromosome conformations are distinguished by territory domains

Differences in chromosome structures between clusters become more apparent when we compute the average contact frequency maps and distance matrices from all chromosomes in each cluster (Fig. 2D). Every cluster displays a unique contact frequency pattern (Fig. 2D). Most clusters (and 60% of all clustered chromosomes) show visible boundaries in the average distance and contact frequency matrices that divide the chromosome territory into spatial subdivisions, with increased contact frequencies within, and reduced contact frequencies between territory domains (e.g., clusters 1, 3, 6, and 8 in Fig. 2D). These territory domain boundaries are most evident in the average distance matrix of a cluster (Fig. 2D), showing increased distances (i.e., dark blue off-diagonal regions in av. distance matrices) and thus spatial segregation between territory domains (Fig. 2D). Consistently, these boundaries appear as local maxima in insulation score profiles calculated from the averaged cluster distance matrices (Methods) (Additional file 1: Fig. S10, S11, S12) or from single-cell structures (Additional file 1: Fig. S10, S11, S12). They also tend to delineate regions with distinct average radial positions. For instance, cluster 3 contains a territory domain boundary at sequence position 155 Mb (boundary 8 in Fig. 2D,E), which separates a small q-terminal domain *f* (155–171 Mb) from the remainder of the chromosome territory (domain *e*), as depicted in Fig. 2D. A representative chromosome structure of cluster 3 shows the small q-terminal territory domain spatially separated from the bulk of the remaining chromosome territory (Fig. 2D (cluster 3, lower panel)). Noticeably, the territory domain boundary shows relatively low chromatin fiber condensation and thus acts as a hinge region allowing the separation of domain *f* (in cluster 3) from the bulk of the chromosome territories (domain *e*) (see section below, and representative structure in Fig. 2D)).

Cluster 6 exhibits another territory domain boundary at around 134-Mb sequence position (Fig. 2D,E), which separates a slightly larger chromosome territory domain at the q-arm terminal end of the chromosome (domain *l* at 134–171 Mb, Fig. 2D) from the remainder of the chromosome territory (domain *k*).

Instead, cluster 2 contains a relatively small chromosome territory domain at the p-arm terminal end of chromosome 6 (domain ***c*** in cluster 2 (0–14 Mb), Fig. 2D). Overall, territory domain boundaries are located at a few specific locations in the chromosome (Fig. 2E). In chromosome 6, we find a total of 8 territory domain boundaries and a total of 17 territory domains across the 8 chromosome morphology clusters (domains *a*–*q* in Fig. 2D,E, middle panel).

The sequence locations of domain boundaries and the size of territory domains have profound implications for the decay rate of the contact probability P(s) as a function of genomic separation (Fig. 2F). This measure is traditionally used in the Hi-C field to investigate the polymeric nature of chromosome folding patterns. At shorter sequence distances (< 10 Mb), the decay rate is similar for chromosomes in all clusters. However, at larger sequence distances, the decay rate varies substantially for chromosomes in different clusters (Fig. 2F). For chromosomes with no or only a small territory domain (e.g., clusters 2 and 4), the decay rate is similar to those of the ensemble average. However, with increasing number and sizes of territory domains, we observe a decrease in long-range chromatin interaction probability for regions separated in sequence between 10 and 100 Mb, in particular for cluster 8, which contains the most fragmented chromosome morphology with 4 chromosome territory domain boundaries (Fig. 2F). These observations affirm the role of territory domain boundaries on modulating long-range chromatin distances.

### Morphology clusters are validated by imaging and sc-Hi-C experiments

**Fig. 3:**
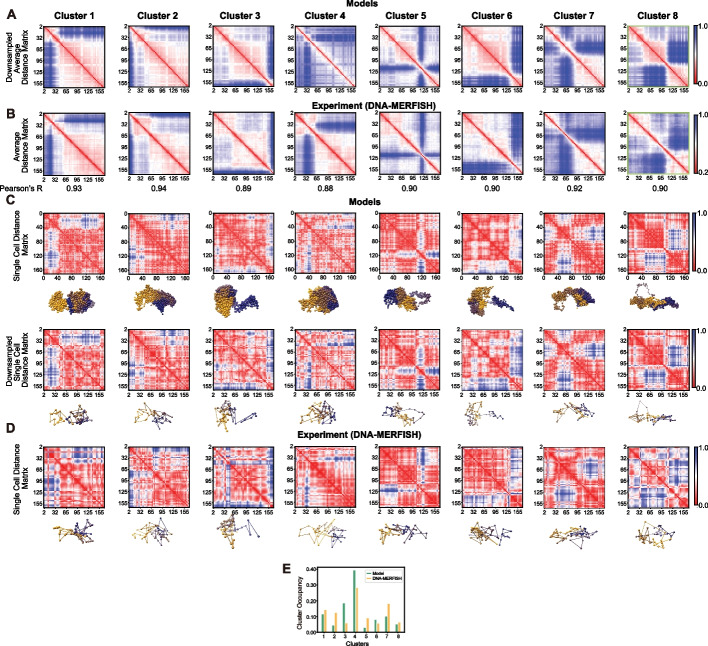
Chromosome clusters can be validated by imaging experiments.** A** Average distance matrices from modeled chromosomes (chromosome 6) in each cluster downsampled to the respective coverage as observed in DNA-MERFISH experiments [6]. Tick labels of all distance matrices indicate sequence location in Mb. For comparison with experiment distance matrices were down sampled to the same coverage in the experiment (window size 3 Mb). **B** The corresponding average distance matrices of chromosomes from DNA-MERFISH experiments for each cluster [6] together with their Pearson’s R with distance matrices from the model. **C** Selected representative examples of single-cell modeled structures and the corresponding downsampled version for chromosome structures for each cluster. Matrices are calculated with window size 200 kb. **D** Average distance matrices and selected representative single-cell chromosome structures from DNA-MERFISH experiment for each cluster. In panels **A**, **B**, lower panel **C** and **D** distance matrices are shown at 3-Mb resolution (coverage in DNA MERFISH experiment); in upper panel **C** the distance matrix is shown at 200 kb resolution.** E** Comparison of the cluster occupancy between the chromosome conformational clusters observed in our models and corresponding chromosome conformational clusters from DNA-MERFISH experiments

We assessed our findings with chromosome tracing data from multiplex DNA-MERFISH imaging [6]. Due to the relatively small sample size (~ 7000 chromosome structures) and low coverage (~ 3 Mb step size), density-based clustering can only recover the 3 clusters with the highest occupancy (clusters 1, 4, and 7) (Additional file 1: Fig. S13). This is also found when clustering a downsampled version of the modeled population, where we find fewer clusters which are still among the original detected clusters at the 200-kb resolution (Additional file 1: Fig. S6). This is anticipated from simulations of density-based clustering, which detects a smaller number of clusters with smaller sampling sizes (Additional file 1: Fig. S5). Nevertheless, more than half of all DNA MERFISH structures show high structural similarity to one of our predicted cluster morphologies (Methods), and thus about 56% of all DNA-MERFISH structures (about 4000 structures) can be classified to one of the eight chromosome morphology clusters (Fig. 3A–D). The average distance matrices for each cluster computed from the classified DNA-MERFISH structures showed high similarity with those from our models, and confirmed the locations of all domain territory boundaries (Pearson’s *r* > 0.88) (Fig. 3A,B). For instance, DNA MERFISH structures confirm the small chromosome territory domain at the p-terminal end of chromosome 6 in clusters 3 and 6 at the sequence location 134 and 155 Mb, respectively, both in distance matrices and single-cell representative structures (Fig. 3A–D). DNA MERFISH also confirmed the more fragmented chromosome morphologies of cluster 8 and cluster 5 (Fig. 3A–D), including the depleted intra-chromosomal interactions for the territory domain *i* (sequence location 114–127 Mb). It is noteworthy that cluster 4 shows also the highest occupancy (28%) in DNA MERFISH structures (Fig. 3E), while about 72% of classified structures contain at least one territory domain boundary.

We also assessed our findings with available single cell Hi-C (sci-HiC) data of GM12878 cells [13] (Additional file 1: Fig. S14AB). To increase the relatively low contact coverage (on average only 3879 contacts per cell at 200 kb resolution), we applied the scHiCluster imputation method [58] (Additional file 1: Fig. S14B). About 8500 out of 11,000 imputed single-cell contact matrices could then be classified based on their similarity to the average contact maps of our detected clusters (Methods) (Additional file 1: Fig. S14B). The average contact frequency maps for each cluster show good agreement with those from our models, including locations of territory domain boundaries (Additional file 1: Fig. S14B). A control experiment, where off-diagonal contact entries in each sci-HiC contact map were randomized, resulted in cluster averages that did not reproduce the contact patterns of our clusters (Additional file 1: Fig. S14C).

### Territory domain boundaries influence long-range chromosome structure variation

**Fig. 4:**
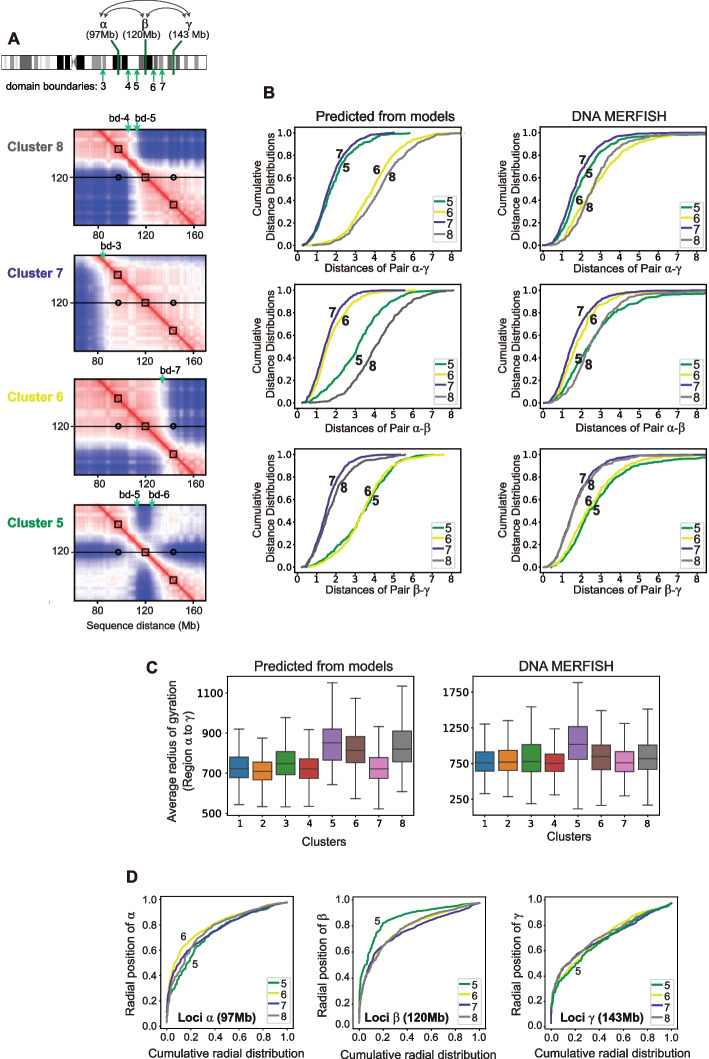
Cumulative distance analysis unveils cell-to-cell variations of chromosome structures influenced territory domain boundaries. (Shown are results for GM12878 cells).** A** The genomic positions of three studied loci α (97 Mb), locus β (120 Mb), and locus γ (143 Mb) showed together with their locations within distance matrices of predicted clusters 5, 6, 7, and 8. **B** (Left column) Cumulative distance distributions of three pairs of selected locations at locus α (97 Mb), locus β (120 Mb), and locus γ (143 Mb) for clusters 5, 6, 7, and 8 of modeled structures. (Right column) Cumulative distance distributions of three pairs of selected locations at locus α (97 Mb), locus β (120 Mb) and locus γ (143 Mb) for clusters 5, 6, 7, and 8 of matched DNA-MERFISH structures [6]. **C** Distributions of average radius of gyration for a downsampled region between 97 and 143 Mb of predicted clusters for both model and DNA MERFISH [6]. **D** Cumulative radial position distributions of three selected locations at locus α (97 Mb), locus β (120 Mb), and locus γ (143 Mb) for cluster 5, cluster 6, cluster 7, and cluster 8 of modeled structures. Note that cluster 5 has a larger radial position distribution than other clusters at locus β

The specific locations of territory domain boundaries greatly influence the long-range distance distributions between genomic regions. For instance, even though loci ${\alpha}$, $\upbeta$, and $\upgamma$ (Fig. 4A) at sequence locations 97, 120, and 143 Mb are equidistant to each other along the sequence, the pairwise 3D distance distributions involving these three regions show substantial differences in clusters 5, 6, 7, and 8 due to differences in the domain boundary locations in each cluster (Fig. 4A,B). For instance, loci $\upbeta$ and $\upgamma$ are located in the same territory domain in clusters 7 and 8, while being in different territory domains in clusters 5 and 6. Subsequently, the cumulative distributions of loci $\upbeta$–$\upgamma$ distances are substantially shifted to smaller values in clusters 7 and 8, while the distance distributions for the same loci pair are shifted to substantially larger values in clusters 5 and 6 (Fig. 4B). In contrast, in clusters 5 and 7 the loci ${\alpha}$ and $\upbeta$ are located in the same territory domain and therefore have distance distributions with substantially smaller distances than in clusters 6 and 8, where the two loci reside in different territory domains. Finally, loci ${\alpha}$ and $\upgamma$, separated by 46 Mb sequence distance, show relatively smaller distances in clusters 7 and 5, where both loci are in the same territory domain, while distances are dramatically shifted to larger values in clusters 6 and 8, where both loci are separated by territory domain boundaries (Fig. 4B). (For cumulative distance analysis for all clusters see SI (Additional file 1: Fig. S15AB)).

The corresponding distance distributions from DNA-MERFISH experiments show the same general behavior (Fig. 4B): regions within the same territory domains have distance distributions shifted to smaller values, whereas distances are shifted to larger values when loci were positioned at opposing sides of a territory domain boundary (Fig. 4B).

Noticeable the 46-Mb region centered at loci $\upbeta$ (stretching between loci $\alpha$ –$\upgamma$, Fig. 4A) also shows a substantially larger radius of gyration in cluster 5 than in other clusters, in both, our models and DNA MERFISH clusters (Fig. 4C).

### Chromosome morphologies influence nuclear locations of chromosomal regions

Next we aim to address the following question: Does the morphology of a chromosome structure influence the nuclear positions of its chromosomal regions? We can analyze chromosome structures within the nuclear topography, for instance, by measuring the radial positions of chromosomal regions. Loci $\upbeta$ (at sequence location 120 Mb) is part of a small territory domain in cluster 5 (domain ***i***: 114–127 Mb in Figs. 2D, 4A) and is bordered by two territory domain boundaries (boundary bd-5 and bd-6 in Fig. 4A). This region has very low gene density and is mostly composed of chromatin of the inactive B-compartment, specifically, the B3 subcompartment annotation [31]. It is noticeable that this region shows significantly more peripheral radial position in cluster 5 than in any other cluster (*p*-values of Welch’s *t*-test for cluster 5 vs cluster 6, 7, 8 = 3.16e − 08, 1.39e − 13 and 3.37e − 08 (Additional file 1: Table S2)). For instance, the cumulative radial distribution of loci $\upbeta$ is dramatically shifted to higher peripheral radial positions in cluster 5 than in clusters 6, 7, and 8 (Fig. 4D, middle panel), while the neighboring loci ${\alpha}$ and $\upgamma$ show relatively more interior locations in cluster 5 than in clusters 6, 7, and 8 (Fig. 4D, left and right panels). This means that domain *i* (including loci $\upbeta$) in cluster 5 protrudes out of the chromosome territory towards the periphery of the nucleus (Fig. 4D), which explains the loss of intra-chromosomal interactions and increased spatial distances of this region in the average distance matrix (Fig. 2D). When loci $\upbeta$ is part of other, larger territory domains in clusters 6, 7, and 8, it shows substantially more interior radial locations (Fig. 4D, middle panel). (Cumulative radial distributions for all clusters are shown in SI (Additional file 1: Fig. S15CD)).

**Fig. 5:**
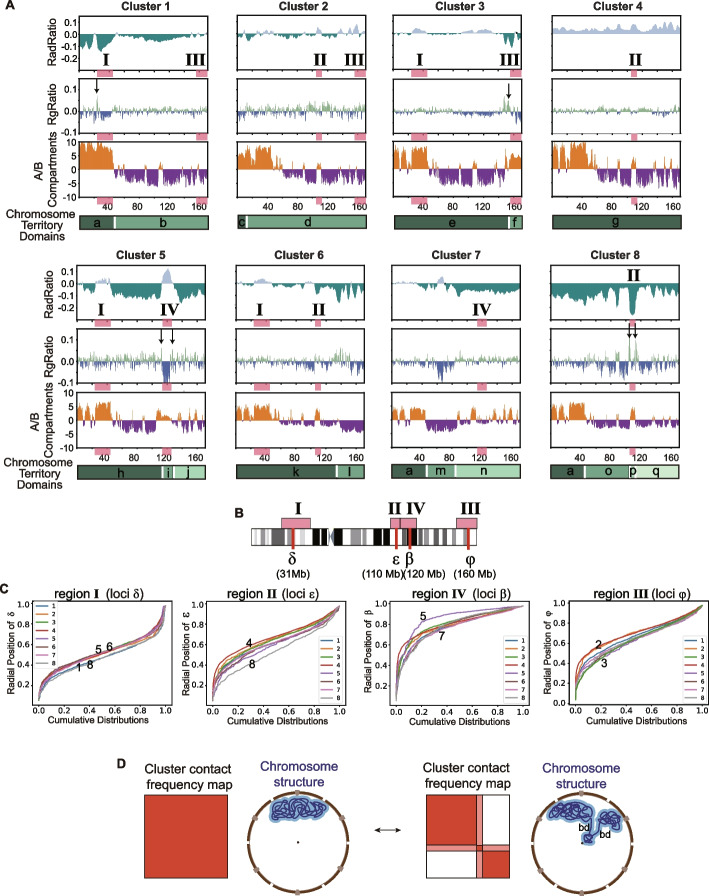
Chromosome morphologies show preferences in nuclear locations. (Shown are results for GM12878 cells). **A** For each cluster the following information is shown (Methods): (top panel) RadRatio: the log fold ratio of the average radial position in the cluster with respect to full ensemble average. (A negative RadRatio value indicates that its average radial position in the cluster is closer to the nuclear interior than in the overall population as a whole.); (second panel from top) RGRatio: the log fold ratio of RG in the cluster with respect to the value in the full ensemble; (third panel from top) the A/B compartment profile from the averaged contact frequency matrix of chromosomes in the cluster; (fourth panel from top) different shades of green indicate the location of chromosome territory domains. Also shown as labeled red blocks are the genomic positions of 4 selected regions I, II, III, and IV. These regions show particularly different behavior in RadRatio and RGRatio between the clusters: (region I (24–48 Mb), II (105–114 Mb), III (155–171 Mb), and IV (114–127 Mb)). **B** The genomic positions of the 4 selected regions I, II, III, and IV in a schematic chromosome as well as 4 selected loci (δ (31 Mb), ε (110 Mb), β (120 Mb), and φ (160 Mb)) on chromosome 6. **C** Cumulative radial position distributions of four selected locations at locus δ (31 Mb) of region I, locus ε (110 Mb) of region II, locus β (120 Mb) of region IV, and locus φ (160 Mb) of region III. **D** Proposed scheme of switching between different conformational states

To better quantify cluster-specific preferences in radial positions of genomic regions, we compute the log ratio between the average radial positions in a cluster and the ensemble of all clustered chromosomes (RadRatio) (Methods) (Fig. 5A, second profile panel from top). We see that RadRatio profiles differ substantially between all clusters of chromosome 6, in particular for clusters 1, 3, 5, 6, and 8, which show pronounced positive and negative peaks in the RadRatio profile (Fig. 5A). A negative RadRatio value indicates that the average radial position of a genomic region is shifted to the nuclear interior in a cluster in comparison to structures of the cell population as a whole. For instance, the RadRatio profiles in clusters 1 and 3 differ substantially across the entire chromosome (Fig. 5A). Among the eight clusters, four specific genomic regions particularly differ in their RadRatio profiles (regions I (24–48 Mb), II (105–114 Mb), III (155–171 Mb), IV (114–127 Mb)) (Fig. 5A,B). Region I contains the MHC gene cluster at location 28.5–33.5 Mb. In cluster 1, region I is significantly shifted towards more interior nuclear positions in comparison to the same region I in clusters 3, 5, and 6, which even show more exterior nuclear locations than in the population average (*p*-values of Welch’s *t*-test [59] = 4.06e − 12, 1.02e − 03 and 6.17e − 09, respectively, for comparing cluster 1 to clusters 3, 5, and 6 (Additional file 1: Table S2)) (Fig. 5A–C). The genomic region II forms a small territory domain in cluster 8 (domain *p* in cluster 8, Fig. 5A,B), which shows strongly negative RadRatio, and therefore loops towards the nuclear interior. Instead, the same region II in cluster 6 is part of a larger territory domain with significantly higher RadRatio (*p*-values = 1.04e − 09, Welch’s *t*-test for clusters 8 and 6 (Additional file 1: Table S3)), while in cluster 2 the same region II is even shifted towards the nuclear periphery in comparison to the population average (see negative RadRatio for regions II in cluster 2 in Fig. 5A) (*p*-values = 4.95e − 17, Welch’s *t*-test for clusters 8 and 2 (Additional file 1: Table S3)). In cluster 3, region III forms a small chromosome territory domain at the q-terminal chromosome end (domain *f* in cluster 3 in Figs. 2D, 5A,B). This territory domain loops towards the nuclear interior in cluster 3, as shown by the negative RadRatio profile and the representative structures (Figs. 5A, 2D). In clusters 1 and 2, the same region III shows a more exterior nuclear location and does not form a separate domain, but is part of a larger chromosome territory domain (Fig. 5A). In addition, region IV (114–127 Mb), the aforementioned region that forms the small domain *i* in cluster 5, is gene poor and contains the long gene NKAIN2 that spans about 1-Mb genomic DNA, extends towards the nuclear exterior in cluster 5, while the same region IV in cluster 7 is embedded in a larger territory domain and shows a significantly more interior nuclear location (Figs. 5A–C, 2D) (*p*-values = 1.39e − 13, Welch’s *t*-test (Additional file 1: Table S4)). Moreover, in cluster 4, without chromosome territory boundaries, the radial positions for almost all chromosomal regions are shifted towards the nuclear periphery in comparison to the population average (see positive values in RadRatio profile in Fig. 5A) (Additional file 1: Table S3)) (Fig. 5C).

### Chromatin A/B compartments

To gain insights into how chromosome conformations influence gene function, we calculated A/B compartments in each cluster by eigenvector decomposition of its contact frequency matrix following the approach in Rao et al. [31] ((Fig. 5A third panel and Additional file 1: Fig. S16). The first principal component (PC1) corresponds to A and B compartment patterns in clusters 1, 2, 3, 4, and 5. For clusters 6, 7, and 8, the second principal component (PC2) correlates with A-B compartments, while PC1 correlates with territory domains (Additional file 1: Fig. S16). This may be explained by the larger number of domains and relatively large changes in radial positions at domain boundaries, in particular for clusters 7 and 8. Our observations are similar to a recent study in mouse cortex brain cells [60], where PC1 components correlated with chromosomal megadomain structures in some neuronal cells, while the PC2 component correlated with A/B compartments. Interestingly, we observe considerable differences in A/B compartment annotations across clusters (Pearson’s correlations to ensemble PC1 profile range between 0.72 and 0.99). Notably, chromatin regions exhibiting the greatest structural variations between clusters (regions I–IV) also show the largest shifts in A/B compartment eigenvalues (Fig. 5A). The most striking difference occurs at the p-terminal end of chromosome 6 in cluster 3 (region III), which shifts from a B to an A compartment, while in most other clusters it remains in the B compartment. Similarly, region IV is annotated as part of the A compartment only in cluster 5, while in all other clusters it remains in the B compartment. Additionally, the q-terminal regions in cluster 1 (including region I) display substantially higher positive eigenvalues compared to other clusters. These findings suggest that shifts in chromosome conformations may be associated with changes in chromatin functional properties.

### Chromatin fiber condensation

Chromosome morphologies show also differences in the local chromatin fiber condensation, which is measured by the radius of gyration (RG) over a sliding 1-Mb window [17, 19]. Differences are best illustrated by the log ratio between the average RG values in a cluster and the overall ensemble average (RgRatio) (Methods) (Fig. 5A, second panels from top). The RGRatio shows substantial differences between the clusters, most noticeable at territory domain boundaries that coincide with major transitions in the radial positions of adjacent genomic regions (Fig. 5A). For instance, at region II (105–114 Mb) in cluster 8 forms a small territory domain (*p*) that loops towards the nuclear interior (Fig. 5A–C). The two domain *p* boundaries at region II (105–114 Mb) show distinct maxima in the RGRatio values, indicating a more extended chromatin fiber in cluster 8 to allow for the radial transition of domain *p* (Fig. 5A, B, indicated by two arrows). This is also shown in representative structures of cluster 8 (Fig. 2D) showing an extended fiber at the domain boundary, acting as a hinge region to facilitate the radial transition of domain *p* towards the nuclear interior (Figs. 2D, 5D). Similarly, the gene-poor region IV (114–127 Mb), which extends towards the nuclear exterior, is flanked by regions with increased fiber extension (peaks in RGRatio), while region IV itself shows substantially higher fiber compaction in cluster 5 than in the ensemble average (i.e., negative RgRatio values) (Fig. 5A–C, indicated by two arrows, 5A).

### Chromosome morphologies influence inter-chromosomal interactions

**Fig. 6:**
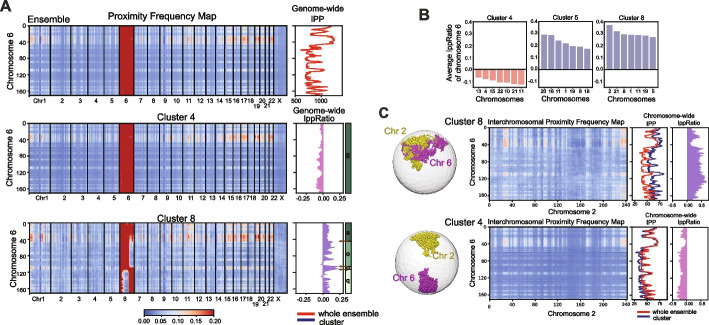
Comparison of inter-chromosomal proximity frequency map and associated features for chromosomes in different clusters (Shown are results for GM12878 cells). **A** (left panels) The average proximity frequency matrix between structures of chromosome 6 and structure of all other chromosomes in the genome for different clusters (Methods), (right panels) Inter-chromosomal proximity profile (IPP), defined as the total number of inter-chromosomal contact proximities of a genomic region with any other chromosomal region of any chromosome divided by the total number of genome structures in a cluster (Methods). The red line shows the genome-wide IPP profile calculated from the whole ensemble of structures, while the blue line shows the IPP profiles calculated from the structures in each cluster. IppRatio, defined as the log ratio of IPP values in a cluster over the IPP value calculated from the ensemble of all clustered structures. Each row of panels shows these properties calculated from different clusters, namely clusters 4, and 8. **B** Ranking of the average IppRatios between chromosome 6 and the other chromosomes in different clusters. Only the 7 top ranked chromosomes leading to the highest averaged IPPRatio are shown.** C** (top left panel) Interchromosomal proximity frequency map between chromosome 6 and chromosome 2 calculated from structures in cluster 8 and (bottom left panel) and structures in cluster 4. (Top middle panel) IPP profile of chromosome 6 considering interactions only to structures of chromosome 2 in cluster 8 (blue curve) and (bottom middle panel) in cluster 4 (blue curve). For comparison also the ensemble profile as in **A** are shown in red (Top right panel). IppRatio profiles between chromosomes 6 and 2 in cluster 8 and (Bottom right panel) in cluster 4. Also shown are representative structures of chromosome 6 and 2 in cluster 8 (top left) and cluster 4 (bottom left)

For most genomic regions, interchromosomal proximity matrices vary considerably between different morphology clusters (Fig. 6A, left panels and Additional file 1: Fig. S17). To quantify these differences, we computed the IppRatio, defined as the logarithmic ratio of the total count of a genomic region’s inter-chromosomal contacts within a cluster and the total count across all structures in the population (Methods) (Fig. 6A, right profile panels). For instance, region II (105–114 Mb) in cluster 8 (i.e., domain *p*) shows higher IppRatio in cluster 8 than in any other cluster (Fig. 6A), partially because of the more interior and exposed location of this region in cluster 8 (Fig. 6A, lower right panels). Overall chromosome morphologies with territory domain boundaries show higher propensity for interchromosomal interactions. Cluster 4 containing chromosomes without territory domain boundaries displays reduced inter-chromosomal interactions than clusters 5 and 8 (Fig. 6B,C). Interestingly, chromosomes in different clusters favor interactions with different chromosomes (Fig. 6B,C). Chromosome 6 in cluster 8 shows the highest average IppRatio with chromosomes 2, 21, and 8, while cluster 5 shows highest interchromosomal interactions with chromosome 20 and 16 instead (Fig. 6B,C).

### Chromosome morphologies influence gene functions

**Fig. 7:**
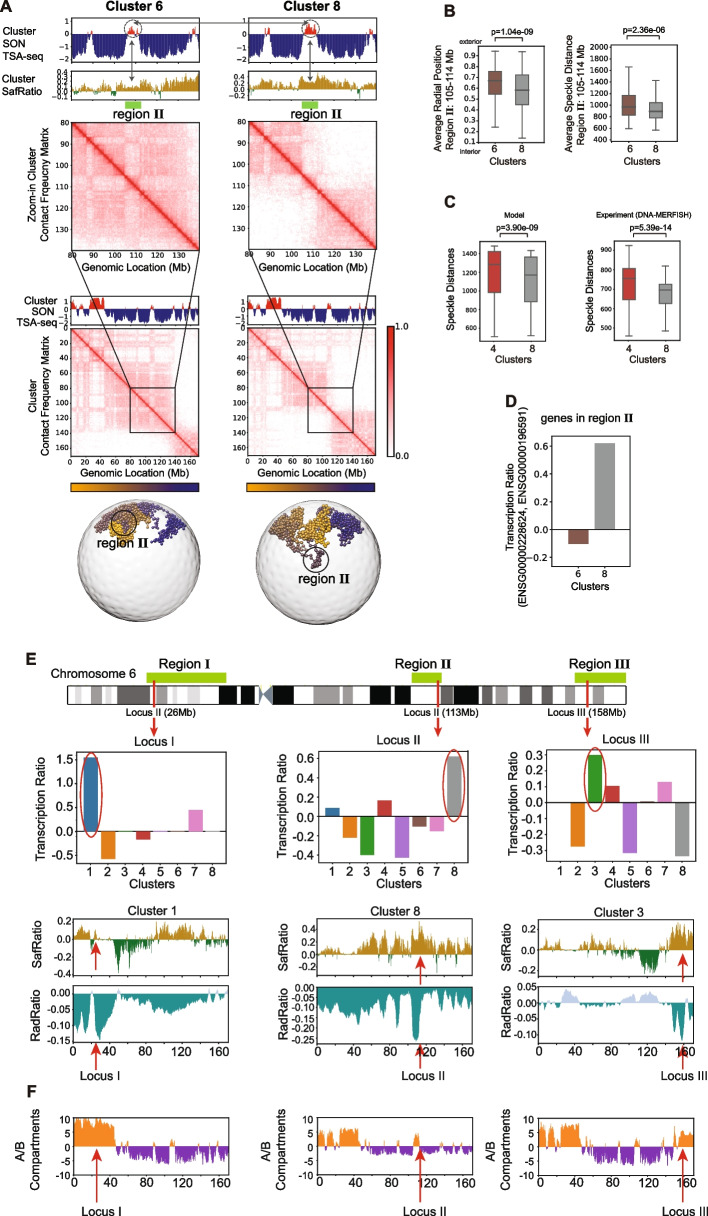
Potential linkage between chromosome morphologies and gene functions (Shown are results for GM12878 cells).** A** Contact frequency matrices and 4 profiles of different structural properties for chromosome 6 in clusters 6 and 8. (Top panel) SON TSA-seq predicted from genome structures in each cluster. Positive values indicate shorted mean distances to nuclear speckles. (Second panel) SafRatio, log ratio of SAF calculated from chromosomes in the cluster over SAF calculated from structures in the whole ensemble (Methods). (third panel from top) Average contact frequency matrices calculated from structures in the cluster. The first four panels show data for a zoomed-in genomic region in chromosome 6. The fifth and sixth panels from top show the predicted cluster SON TSA-seq and cluster average contact frequency matrices for the full-length chromosome 6. The bottom panel shows selected representative structures for chromosome 6 in each cluster. Also indicated is region II (genomic location (105–114 Mb)), which is discussed in the text. **B** Distributions of the average radial position and average speckle distances for a region II (genomic location: 105–114 Mb) in clusters 4, 6, and 8 as well as the *p*-values of Welch’s *t*-test [59] between two pairs of clusters. Differences between clusters of the radial positions and average speckle distances are significant. **C** Distributions of speckle distance (SpD) of all imaged chromosomal regions in cluster 4 and cluster 8 from models (left panel) and from DNA MERFISH experiments (right panel). Distances are stat. significant different between cluster 4 and 8 for both models and experiments. *p*-values of the paired *t*-test for both model and DNA MERFISH are shown [6]. We found the average SpD of cluster 4 is larger than that of cluster 8 in both model and DNA MERFISH. **D** The transcription ratio, log ratio of average transcription level calculated from the DNA-MERFISH cluster over average transcription level calculated from all clustered DNA-MERFISH structures of a locus from region II with genes ENSG00000228624 and ENSG00000196591 for cluster 6 and cluster 8 [6]. **E** (Top panel) The genomic positions of region I (24–48 Mb), region II (105–114 Mb), and region III (155–171 Mb) together with locus I (26 Mb), locus II (113 Mb), and locus III (158 Mb) on chromosome 6. (Second panel from top) The transcription ratio of the three selected locus I, II, and III for all clusters measured by DNA-MERFISH [6] (Methods) (Third panel from top) SafRatio of cluster 1, 8, and 3. (Fourth panel from top) RadRatio of cluster 1, 8, and 3. **F** A/B compartments (PC1) of clusters 1, 8, and 3

To examine if chromosome morphologies affect the disposition of genomic regions to nuclear speckles, we simulated SON TSA-seq data from the chromosomes in each cluster. SON TSA-seq is an experimental method that measures mean cytological distances to nuclear speckles [49]. We previously showed that we can predict speckle locations and SON TSA-seq data with good agreement to experiments (Pearson’s *r* = 0.87 between prediction and experiment [19] and Pearson’s *r* = 0.79 for speckle association frequency (SAF)) [17, 19]. We found that SON TSA-seq signals (and thus mean speckle distances) vary considerably for specific chromosomal regions in different clusters (Fig. 7A and Additional file 1: Fig. S18). For instance, region II (105–114 Mb) in cluster 8 (i.e., territory domain *p* in cluster 8) shows the highest SON-TSA-seq signals among all clusters (Fig. 7A) and thus its mean speckle distance is significantly smaller than the same region in cluster 6 (*p*-value = 2.36e − 06, Welch’s *t*-test (Additional file 1: Table S2)) (Fig. 7B, right panel). Cluster 4 shows the overall largest speckle distances (*p*-value = 3.90e − 09, paired *t*-test) (Fig. 7C, left panel). This observation is also confirmed by DNA MERFISH experiment [6] (*p*-value = 5.39e − 14, paired *t*-test) (Fig. 7C, right panel). The predicted mean speckle distances in different clusters are also confirmed by high correlations with those from DNA MERFISH experiments (Pearson’s correlations range from 0.71 to 0.81) (Additional file 1: Fig. S19A).

Highly transcribed genes are often found close to nuclear speckles [6, 29, 59] and thus higher SON TSA-seq signals generally correlate with higher transcriptional activity [19, 46–48]. We therefore speculate that in cluster 8 transcriptionally active genes in region II (105–114 Mb) have higher transcription levels than the same genes in cluster 6 or any other cluster. We validated this hypothesis with spatial transcriptomics data from RNA-MERFISH experiments, which is jointly measured with DNA-MERFISH chromosome tracing [6]. We selected genes in regions I, II and III for which RNA MERFISH transcriptomics data is available and which show the largest differences in speckle associations between clusters (highest SafRatio levels). For instance, in region II two such genes (ENSG00000228624 and ENSG00000196591) were measured by RNA MERFISH data [6]. These genes show substantially higher nascent transcription levels in cluster 8 than the same genes in cluster 6 (Fig. 7D). Indeed, cluster 8 shows the highest transcription levels of these genes among all clusters (Fig. 7E), confirming our hypothesis (Fig. 7DE). Similarly we located a gene (ENSG00000224843) within chromosomal region I with available transcription data [6] (locus I in Fig. 7E). In cluster 1, this gene is part of a territory domain that loops towards the nuclear interior showing smaller speckle distances than in other clusters. Notably, also this gene exhibits the highest transcription level in cluster 1 (red arrows indicate gene locus I in Fig. 7E). We also identified three genes (ENSG00000271913, ENSG00000164691, and ENSG00000226032) located at loci III in region III with available RNA MERFISH data. In cluster 3, region III forms a small territory domain at the p-terminal chromosome end, which separates from the bulk chromosome territory and loops towards the nuclear interior showing the smallest speckle distances among all clusters. Indeed, RNA MERFISH experiments confirm that the nascent transcription levels of these genes are highest in cluster 3 than in any other cluster (Fig. 7E). These observations indicate that variations in chromosome conformations correlate with gene transcription levels, possibly through variations in the specific nuclear locations of genes in different conformations. However, further experimental evidence is required to establish a definitive causal link between chromosome conformation and gene expression.

### Characteristic features of chromosome territory domain boundaries

**Fig. 8:**
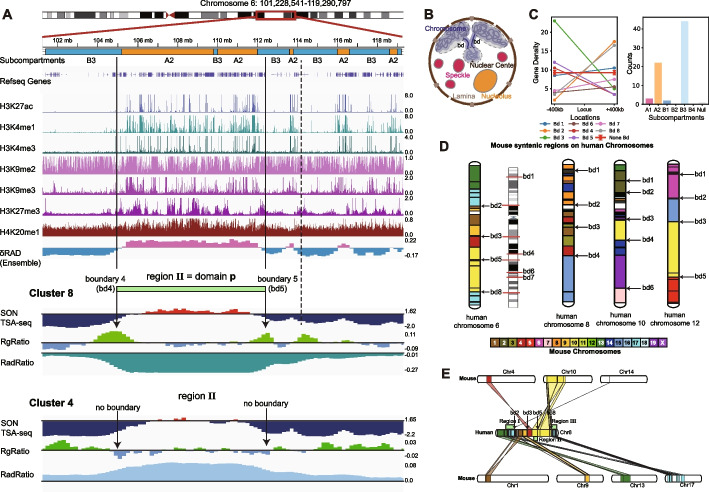
Territory domain boundaries are related with gene function and synteny blocks. **A** Characteristic features for a chromosomal region spanning across region II, which forms territory domain 3 in cluster 8 and the same region II in cluster 4. Shown are epigenetic marks and other features for this sequence region. From the top to the bottom, the displayed features are chromosome sequence location, Hi-C subcompartments, refseq genes, H3K27ac, H3K4me1, H3K4me3, H3K9me2, H3K9me3, H3K27me3, H4K20me1, and the ensemble structural variability (δRAD) calculated from all structural models. In addition, the following features are shown for the same regions calculated from cluster 8 and cluster 4: SON TSA-seq, RgRatio, and RadRatio (Definitions as in Figs. 2 and 4, Methods). Also shown are lines that indicate the territory domain in cluster 8 and corresponding domain boundaries that overlap with regions of reduced chromatin compaction (RGRatio) (bd1 and bd2). For bd2 two alternative boundaries exist in the cluster (bd2 and bd2’). **B** Illustration of schematic features of a chromosome morphology with three territory domains. Shown are also nuclear bodies. bd regions indicate domain boundaries that show increased decompaction of the chromatin fiber (i.e., RG) in comparison to the ensemble average, and which allow the territory domain to loop towards the nuclear interior, while other territory domains remain at the periphery. **C** Average gene density changes from the downstream 400-kb region to the upstream 400-kb region at domain boundary regions (BD) and none domain boundary regions (none BD) with 95% confidence interval. We observe sharper changes of average gene density at domain boundary regions rather than none domain boundary regions. The barplot of subcompartment distribution at domain boundary regions (BD) together with their downstream 800-kb regions and the upstream 800-kb regions of domain boundary regions (BD), where we observe dominant A2 and B3 proportions. **D** Illustration of synteny blocks of human chromosomes 6, 8, 10, and 12 shown together with alignments between boundaries of synteny blocks and domain boundaries found by the model. **E** Illustration of synteny blocks of human chromosome 6 and their mapping from mouse chromosomes 1, 4, 9, 10, 13, 14, and 17. Four domain boundaries related with region I, II, and III can be aligned with the boundaries of synteny blocks

Territory domain boundaries are found at a few genomic locations. These regions are often located close to transitions between gene-poor and gene-rich chromosomal regions and coincide often with boundaries between Hi-C subcompartments, as defined by Rao et al. [31] (Fig. 8A–C, Additional file 1: Fig. S20AB). For instance, in chromosome 6 most territory domain boundaries align with boundaries between the A2 and B3 subcompartments (Fig. 8A,C). If a boundary is present in a cluster, it also often coincides with major transitions in the radial position and pronounced peaks in RGRatio profiles (e.g., RadRatio and RGRatio profiles of region II in cluster 8 vs cluster 4) (Fig. 8A,B, Additional file 1: Fig. S20AB). We also noticed that domain boundaries often separate chromosomal regions with generally high and low cell-to-cell variability in their radial positions (see δRAD profiles in Fig. 8A).

**Fig. 9:**
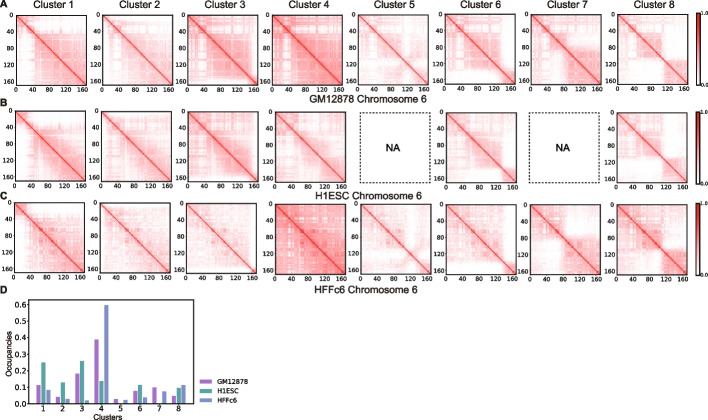
Comparative analysis of predicted clusters of chromosome 6 from genome structures of GM12878, H1-hESC, and HFFc6 cells.** A** The contact frequency matrices of the 8 predicted clusters of chromosome 6 in GM12878 cells. **B** The contact frequency matrices of the predicted clusters of chromosome 6 in H1-hESC cells. Clusters 5 and 7 were not observed in H1-hESC cells and are indicated by “N/A”. **C** The contact frequency matrices of the predicted clusters of chromosome 6 in HFFc6 cells. **D** Comparison of the cluster occupancy between the chromosome conformational clusters observed in GM12878, H1-hESC, and HFFc6 cells

Strikingly, territory domain boundaries often overlap with synteny breakpoints, which separate syntenic blocks. In syntenic blocks, the collinearity (i.e., order) of homologous genes are conserved across genomes of different species. Syntenic breakpoints are boundaries between syntenic blocks where genomic rearrangements have occurred during evolution. Strikingly, 4 out of 8 territory domain boundaries in chromosome 6 and 50–83% territory domain boundaries of all chromosomes are found at syntenic breakpoints between the mouse and human genome (Fig. 8D, E). Most dramatically this is seen in chromosome 12, where almost all major syntenic breakpoints align with chromosome territory domain boundaries (Fig. 8D). We can only speculate why some syntenic breakpoints coincide with territory domain boundaries. It is possible that syntenic blocks contain genes that are functionally constrained to be colocalized and thus must maintain this co-localization in chromosome conformations. It is also possible that the location of a territory domain boundary may increase the probability for a chromosomal rearrangement at this particular sequence location during evolution. If syntenic breakpoints align with territory domain boundaries in GM12878 cells, then they should also be found in other human cell types. We applied our clustering protocol to genome structures generated from Hi-C data of two additional cell types, the human embryonic cell H1 hESC, and the fibroblast cell HFFc6 (Fig. 9A–C). Indeed, in all these cell types we detected clusters with similar locations of chromosome territory domain boundaries. The only exceptions are clusters 5 and 7, which were not detected in H1 hESC cells. Although similar clusters are found in these cell types, the cluster occupancy varies considerably between cell types (Fig. 9D).

## Discussion

Here we present a method to cluster 3D chromosome structures into prevalent subpopulations to study the role of structural stochasticity in gene function. Given the substantial dynamic variability of genome structures within a cell population [38], clustering of whole chromosome structures into prevalent chromosome morphologies poses a significant challenge. To achieve this goal, we introduced a two-step density-based clustering method that combines a convolutional autoencoder with t-SNE dimension reduction to cluster large ensembles of single cell 3D chromosome structures into prevalent conformational clusters. Our density-based clustering does not coerce all conformations into a defined set of clusters, but rather detects only those conformational subpopulations that are supported by a substantial amount of structures.

We found that up to half of all structures for each chromosome can be clustered in 5 to 12 prevalent chromosome structure subpopulations (i.e., morphologies), which are distinguished in their long-range (> 10 Mb sequence distance) chromatin interaction patterns. It is possible that with larger population sizes, additional clusters with a smaller occupancy can be detected. Notably, each chromosome morphology is characterized by chromosome territory domains, which partition the chromosome into spatial subdivisions that show weak correlations in their spatial positions to each other. Thus, the sequence locations of these boundaries play a pivotal role in establishing long-range structure variations and are found at a few locations in the chromosome. For instance, in chromosome 6, we observe 8 territory domain boundaries. These boundaries are often located at transitions between gene-poor and gene-rich sections as well as boundaries between A2 and B3 Hi-C subcompartments [31]. Interestingly, about half of the detected territory domain boundaries overlay with locations of syntenic breakpoints. These syntenic breakpoints constitute boundaries where genomic rearrangements have occurred during evolution and they separate syntenic blocks, genomic regions with conserved collinearity of genes. At this point, we do not know why some syntenic breakpoints overlay with chromosome domain boundaries. But it is interesting that we observe a link between the evolutionary history of a chromosome and the dynamic variations of its chromosome conformations. Similar territory domain boundaries are found in different cell types. For instance, human lymphoblastoid cells (GM12878), embryonic stem cells (H1-hESC), and fibroblast cells (HFFc6) all share similar cluster morphologies with similar locations of domain boundaries. However, the relative occupancy of each cluster varies between cell types.

A unique aspect of this work is our ability to analyze chromosome morphologies within the context of the nuclear topography. We found that preferences in radial positions, speckle distances, and lamina proximity of chromosomal regions differ between chromosome morphologies. The nuclear microenvironment of a gene can impact its transcription rate [19]. For instance, shorter distances from nuclear speckles can enhance gene expression levels of actively transcribed genes [46–48]. It is therefore plausible that the differences observed between chromosome morphologies also affect transcription levels of specific genes. In chromosome 6, we discovered three specific regions that show relatively large differences in speckle distances and nuclear locations between different morphologies. Through RNA MERFISH data analysis, we found that genes in these regions showed significant differences in their transcription rate when part of a different chromosome morphology. At this point, the causality between structure and function is unknown, for instance if the chromosome morphology influences the gene function or vice versa. However, our work suggests that chromosome conformations potentially contribute to the cell-to-cell heterogeneity of gene transcription observed in single-cell RNA-seq experiments [61, 62]. Such information can be important for unveiling the role of genome structure in the regulatory processes of genome function.

An open question pertains to how the inception of a territory domain boundary is regulated. A territory domain boundary is often associated with an extended chromatin fiber at the boundary region. It is conceivable that reduced occupancy of certain architectural proteins at specific locations could promote such a chromatin fiber decompaction and thus favoring the emergence of a particular domain boundary. It was recently shown that downregulation of cohesin loaders leads to a more compact chromosome conformation in vivo [63]. At this stage, this explanation is speculative, and further research is required to offer further insights. It will be interesting to test in future under which conditions polymer simulations could reproduce the observed territory domains at predicted locations. For instance, it would be interesting if loss of CTCF loop extrusion barriers at specific regions would favor macrodomains at specific sites, or if specific block polymer states could similarly reproduce the observed territory domains.

Finally, our work illustrates how genome structure models can aid the classification of chromosome tracing data obtained from multiplexed FISH imaging. At limited coverage and population size, clustering chromosome structures from tracing experiments is challenging. However, it is feasible to cluster chromosome structures at higher resolutions and coverage from data-driven modeling approaches, such as IGM [17]. These structural clusters can then facilitate the classification of chromosome morphologies from multiplexed FISH data.

## Conclusions

Chromosomes show substantial variability in their structure conformations among individual cells. This dynamic variability poses great challenges for structure–function analysis, necessitating a method to identify prevalent subpopulations of chromosome structures. Here, we proposed a two-step dimension reduction method, which allowed us to identify the existence of conformational subpopulations with distinct long-range chromatin interaction patterns. Our findings indicate that variations in chromosome conformations could contribute to the cell-to-cell heterogeneity of gene transcription. A critical factor for establishing chromosome structure subpopulations is the presence of territory domain boundaries, often located at evolutionary syntenic breakpoints, which could introduce physical properties that could favor their locations at these locations.

## Methods

### Population-based modeling

We used ensemble Hi-C data with the Integrative Genome Modeling (IGM) platform [17] to generate one 10,000 whole genome structure population for HFFc6 (raw Hi-C data from 4DN Data Portal, accession code 4DNES2R6PUEK [24]) and H1-hESC (raw Hi-C data from 4DN Data Portal, accession code 4DNES2M5JIGV [24]) cell lines, and used a previously generated and analyzed 10,000 structure population for GM12878 [19].

IGM simulates a population of structures that is compatible with the available ensemble Hi-C data by optimizing the positions of the chromatin regions. Let ${{\varvec{X}}}_{{s}}=\{{{\varvec{x}}}_{1{s}},\boldsymbol{ }\dots ,\boldsymbol{ }{{\varvec{x}}}_{{N}{s}}\}$ denote a diploid whole genome structure of $N$ regions, ${{\varvec{x}}}_{1{s}}\in {\mathbb{R}}^{3}$ being the Cartesian coordinates of the $i$ th genomic region. A population of structures is defined as a collection of $S$ such structures$\varvec{X}=\{{\varvec{X}}_{1 },\dots , {\varvec{X}}_{S }\}$. Also, let $\varvec{A}={\left({a}_{IJ}\right)}_{H\times H}$ denote the Hi-C contact probability matrix, so that ${0\le a}_{IJ}\le 1$ indicates the probability that two unphased loci $I$ and $J$ ($I, J\in \{1, \dots , H\}$) are in contact. In the following, we will denote with (lowercase) $i$ and $i{\prime}$ the two copies associated with unphased region $I$ (uppercase). Our genome simulation approach numerically approximates the solution to the optimization problem$\widehat{\varvec{X}}=argma{x}_{\varvec{X}}P\left(\varvec{A}|\varvec{X}\right)$, where $P\left(\varvec{A}|\varvec{X}\right)$ is the probability that a population of structures $\varvec{X}$ reproduces the input contact matrix ***A***. However, this poses major difficulties: first of all, it is an extremely highly dimensional maximization problem. Second, the input data $\varvec{A}$ does not provide information on which contacts coexist within the same structure in the population and, since it is unphased, does not specify which alleles in the representation (either $i$ or$i{\prime}$, $j$ or$j{\prime}$) are actually in contact. In order to account for this missing information, we introduce an indicator tensor$\varvec{W}={\left({w}_{ijs}\right)}_{N\times N\times S}, H\le N$, such as ${w}_{ijs}=1(0)$ indicates that loci $i$ and $j$ are (not) in contact in diploid structure s-th. It is then essential to jointly optimize both $\varvec{X}$ and $\varvec{W}$ variables, i.e.

$$\widehat{\varvec{X}}, \widehat{\varvec{W}}=argma{x}_{\varvec{X}, \varvec{W}}P\left(\varvec{A}, \varvec{W}|\varvec{X}\right)$$

We adapted a hard Expectation–Maximization algorithm that uses a series of numeric strategies for efficient and scalable model estimation to tackle such a daunting task. We first initialize the chromatin structures in random territories, and then we start an iterative optimization, where $\varvec{W}$ and $\varvec{X}$ are alternatively optimized. Each iteration consists of one Assignment step (A-step), where a given subset of contacts from the input Hi-C matrix are optimally allocated across the structures ($\varvec{W}$ is optimized), and a Modeling Step (M-step) where the structure coordinates are optimized using Simulated Annealing Molecular Dynamics and Conjugate Gradient ($\varvec{X}$ is optimized). Additional batches of chromatin contacts are gradually added in each iteration, so as to improve and facilitate overall convergence. Upon convergence, a population $\widehat{\varvec{X}}$ of single-cell whole genome structures are available, which are statistically consistent with the input ensemble Hi-C matrix $\varvec{A}$, and also predict a number of orthogonal observables. More details on IGM formulation and implementation can be found in Boninsegna et al. and Tjong et al. [17, 51].

Preliminary raw Hi-C datasets preprocessing into a 200 k base pair resolution contact probability matrix was accomplished by following the protocol detailed in Yildirim et al. [19].

### Genome representation

Chromosomes are represented in our models as homopolymer chains of monomers at 200-kb base-pair resolution, so that the full diploid genome is represented with *N* = 30,332 monomers for GM12878 and *N* = 29,838 for both H1-hESC and HFFc6. Each 200-kb chromatin region is modeled as a sphere of radius around ${R}_{bead}=118 nm$ in all cell lines, so that the ratio of the genome volume to the nuclear volume is 0.4 [19, 51]. The nuclei for GM12878 and H1-hESC are modeled as spheres of radius ${R}_{nuc}=\text{5,000} nm$ [19, 51]. The nucleus for HFFc6 is modeled as an ellipsoid of semiaxes $\left(a,b,c\right)=(\text{7,840} nm, \text{6,470} nm, \text{2,450} nm)$ [17].

### Two-step dimension reduction

The basic aim of this study is selecting representative single-cell structures from our population and studying their significance. Multiple features of a single-cell structure can be extracted and calculated, such as contact matrix and distance matrix, which can be further calculated as a feature vector. Due to the high dimension of the feature vector of our 200-kb model, direct classification of these feature vectors is unrealistic. We here introduce the two-step dimension reduction that preserves both efficiency and accuracy during the dimension reduction.

#### Removal of unrestrained beads

For each single structure, we remove those beads that are not restrained. All beads remaining in the structure belonging to the “domain” category (not centromeres or telomeres) are considered to construct the distance matrix, while beads belonging to “cen” (centromeres or telomeres) are removed.

#### Input distance matrix

The distance matrix ${\varvec{D}}^{(s)}=({d}_{ij}^{(s)})$ of chromosomal structure *s* is calculated by the surface-to-surface distance between bead $i$ and bead $j$:

$${d}_{ij}^{(s)}={\| {{\varvec{x}}}_{is}-{{\varvec{x}}}_{js}\| }_{2}-2{R}_{bead}$$

where ${{\varvec{x}}}_{is}$ and ${{\varvec{x}}}_{js}$ are the 3D coordinates of bead $i$ and bead $j$ in structure *s* and ${R}_{bead}$ is the bead radius in our model. ${d}_{ij}^{(s)}$ is set to be 0 if $i=j$. The matrix is then applied normalization to ensure the maximum entry in the matrix is 1 (dropping the structure superscript *s*):

$${d}_{ij}{\prime}=\frac{{d}_{ij}}{ {max}_{k, l} {d}_{kl}}$$

#### Convolutional autoencoder

Convolutional autoencoders are frequently used in image classification. Here we adopt the elements from a frequently used convolutional neural network (AlexNet) [64] which is made up of several convolutional and max pooling layers. In this study, we regard each input distance matrix as an image, which is then regarded as the input of the input layer. The autoencoder consists of an encoder and a decoder, where the input distance matrix is the input of the encoder while the latent matrix is the output. The latent matrix is then used as the input of the decoder to generate the final output. We perform 15 epochs with batch size 200 to train the autoencoder after shuffling the input dataset. The autoencoder is implemented by the python package keras https://github.com/keras-team/keras.

##### Processing of input distance matrices

Due to the size of the layers in the convolutional autoencoder, the input matrix is resized to multiples of 50 by bilinear interpolation so that the size of the input matrix matches the reconstructed output matrix by the autoencoder. This is required so that the size is divisible by $50=5\times 5\times 2$, because our autoencoder contains downsampling and upsampling layers, two of which are of size $(5, 5)$ and size $(2, 2)$.

##### Convolutional layer

A convolutional layer performs convolution operation to an original input over each window and constructs a new output. We use three convolutional layers in the encoder and four convolutional layers in the decoder. In the encoder, the first layer has 16 filters with a kernel with size $(10, 10)$. The next two layers each have 8 filters with a kernel with size $(10, 10)$ and 4 filters with a kernel with size $(10, 10)$. We use the ReLU activation function to process the output of each convolutional layer:

$$ReLU(x)=max(0, x)$$

In the decoder, the first layer has 4 filters with a kernel with size $(10, 10)$. The next two layers each have 8 filters with a kernel with size $(10, 10)$ and 16 filters with a kernel with size $(10, 10)$. We use the ReLU activation function to process the output of these convolutional layers. To generate the output, the last convolutional layer uses 1 filter with a kernel with size $(10, 10)$, we use the sigmoid activation function to ensure that the values in the final output matrix are located between 0 and 1:

$$Sigmoid(x)=\frac{1}{1+{e}^{-x}}$$

For each convolutional layer, we use the stride size $(1, 1)$ and the same padding size to ensure the output has the same height and width as the input.

##### Max pooling layer

A max pooling layer is used to downsample an original input by calculating the maximum value in each window and generate a new value. We use three max pooling layers in the encoder. The first two layers have a pooling window with size $(5, 5)$. The last layer has a pooling window with size $(2, 2)$. We use the same padding size for each max pooling layer to generate the output. The stride size is the same as the window size for each layer.

##### Upsampling layer

An upsampling layer is used to up sample an original input by filling each window with the corresponding value. We use three upsampling layers in the decoder. The first layer has a sampling window with size $(2, 2)$ and the next two layers have a sampling window with size $(5, 5)$.

##### Latent vector

The latent vector is generated by directly flattening the latent matrix. We then use standard normalization to normalize the whole set of latent vectors to ensure that each dimension ${{\varvec{x}}}_{l}{\prime}=({x}_{l1}{\prime}, {x}_{l2}{\prime}, .., {x}_{lN}{\prime})$ of the set of new vectors has mean 0 and standard deviation 1:

$${x}_{li}{\prime}=\frac{{x}_{li}-\overline{{x }_{l}}}{{\sigma }_{l}}$$

where $\overline{{x }_{l}}$ and ${\sigma }_{l}$ is the mean and the standard deviation of each dimension ${{\varvec{x}}}_{l}=({x}_{l1}, {x}_{l2}, ..., {x}_{lN})$ of the set of original vectors. $N$ is the size of the training set. After the operation of the encoder, the size of the latent vector is about 700 times smaller than the number of entries in the input matrix, which significantly reduces the dimension size. In other words, longer chromosomes result in bigger latent vectors.

##### Mean squared error

We use the mean squared error (MSE) to calculate the loss between the input matrix ${{\varvec{M}}}^{input}$ and the output matrix ${{\varvec{M}}}^{output}$, which is the mean Euclidean distance between the input matrix and the output matrix of the training dataset:

$$MSE=\frac{1}{N}{\sum }_{i=1}^{N}{\| {{\varvec{M}}}_{i}^{input}-{{\varvec{M}}}_{i}^{output}\| }_{2}^{2}$$

where $N$ is the size of the training set.

##### Optimizer

We use an optimizer that applies the Adadelta algorithm [65] to train the autoencoder. In comparison with other gradient descent methods, this method does not require setting of the learning rate parameter and is relatively more robust.

#### T-distributed stochastic neighbor embedding

T-distributed stochastic neighbor embedding (t-SNE) is a robust and nonlinear dimension reduction method [54]. By using proper probability distributions $\varvec{P}=({p}_{ij})$ and $\varvec{Q}=({q}_{ij})$ to measure similarities between data points in both the original space and the lower dimensional space. The method facilitates the embedding by minimizing the Kullback–Leibler divergence [66] between the two distributions by:

$$KL(\varvec{P}||\varvec{Q})=\sum_{i\ne j}{p}_{ij}log\frac{{p}_{ij}}{{q}_{ij}}$$

We set the dimension of the embedded space to be 2, the perplexity to be 200 and the learning rate to be 1000. The parameters are selected according to the package suggestions to preserve robustness.

#### Principal component analysis

Principal component analysis (PCA) is a frequently used dimension reduction method which computes the principal components of the data. The method uses singular value decomposition (SVD) of the covariance matrix to construct principal components which are then used to find embedded data points in the lower dimensional space. The dimension of the embedded space is set to be 2.

#### Multidimensional scaling

Classical multidimensional scaling (MDS), which is also known as Principal Coordinates Analysis (PCoA), is another nonlinear dimension reduction [67]. The classical MDS transforms pairwise distances between data points into dissimilarities and minimizes a cost function. We use Euclidean distances as dissimilarity measurement. The dimension of the embedded space is set to be 2.

#### Locally linear embedding

Unlike PCA which projects data points in a linear way, locally linear embedding (LLE) is a nonlinear dimension reduction technique [68]. The method can be viewed as a collection of local PCA which preserves distances within each local neighborhood graph. The dimension of the embedded space is set to be 2.

#### Isomap

Isomap, which is also a nonlinear dimension reduction method, is an extended version of MDS [69]. Specifically, the method uses geodesic distances of each local neighborhood graph as similarity measurement before performing MDS. The dimension of the embedded space is set to be 2.

#### Spectral embedding

Spectral embedding (SE) is another nonlinear dimension reduction method [70]. The method uses eigenvectors of the Laplactian matrix to construct embedded data points in the lower dimensional space. The dimension of the embedded space is set to be 2. All embedding listed above including t-SNE, PCA, MDS, LLE, Isomap, and SE are performed by the python package sklearn [71].

#### UMAP

Uniform Manifold Approximation and Projection (UMAP) uses knowledge of algebraic topology and simplicial complexes to perform dimension reduction [72]. The method is an increasingly frequently used nonlinear dimension reduction method which is often used to compete with t-SNE. UMAP is performed by the python package umap-learn [72]. We set the dimension of the embedded space to be 2 and the learning rate to be 1.0.

### Peak detection

In the 2D embedded conformational space, every data point represents a single structure. The structures that are closed with each other in 2D distance are more likely to have similar conformations. The next step is to sample part of the data points which are representative from the 2D distribution.

#### Outlier removal

To remove outlier data points, we first calculate a pairwise distance matrix of all data points. We then generate the total distance between each data point and all the other data points by calculating the row sum of each row. The data points that have extreme total distances are removed by the 3-sigma rule. We only select data points whose row sums are within 3-sigma range $(\overline{{{s}_{l}}}-3{\sigma }_{l}, \overline{{{s}_{l}}}+3{\sigma }_{l})$, where $\overline{{s}_{l}}$ and ${\sigma }_{l}$ is the mean and the standard deviation of the row sum vector ${\varvec{s}}_{l}$.

#### Kernel density estimation

Then we use bivariate kernel density estimation to calculate the probability density function of the distribution. Given a 2D independent and identically distributed sample $\varvec{X}=({{\varvec{x}}}_{1}, {{\varvec{x}}}_{2}, {{\varvec{x}}}_{3},..., {{\varvec{x}}}_{N})$, for each point we are able to find a density function $p$ so that this set of data points is sampled directly from a distribution with joint probability density function $p$:

$$p\left({\varvec{x}}\right)=\frac{{|\varvec{H}|}^{-\frac{1}{2}}}{N}{\sum }_{i=1}^{N}K({{\varvec{H}}}^{-\frac{1}{2}}({\varvec{x}}-{{\varvec{x}}}_{i}))$$

where ${{\varvec{x}}}_{i}={({x}_{i1}, {x}_{i2})}^{T}$. We choose $K$ to be the Gaussian kernel. The bandwidth $\varvec{H}$ is estimated by Scott’s Rule [73]. The resulting 2D density measures how data points are distributed in the conformation space. Each local maximum of the 2D density is defined as a peak, which is a representative conformation.

#### Grid approximation

We use a $(1000, 1000)$ grid $\varvec{G}$ to approximate probability density function $p\left({\varvec{x}}\right)$ and generate a 2D density matrix $\varvec{P}$. The grid is constructed with the minimum value and the maximum value of each of the two dimensions ${x}_{i1}$ and ${x}_{i2}$:

$$\varvec{G}={\left\{\left({min}_{i} {x}_{i1}, {max}_{i} {x}_{i1} \right), \left({min}_{i} {x}_{i2}, {max}_{i} {x}_{i2} \right)\right\}}_{1000\times 1000}$$

We then calculate the density value by the probability density function $p\left(\varvec{x}\right)$ at each grid point and construct the density matrix $\varvec{P}=({p}_{ij})$:

$${p}_{ij}=p\left(\varvec{G}(i, j)\right)$$

#### Maximum filter

A local maximum is an entry that is larger than all its 8 neighbors in the 2D density matrix $\varvec{P}$. To avoid selecting multiple local maxima in a small area, however, we compare each entry with a larger range of its neighborhood. A maximum filter with size $(5, 5)$ is applied to matrix $\varvec{P}$ to generate another matrix ${\varvec{P}}{\prime}=({p}_{ij}{\prime})$. We then use exclusive disjunction (XOR) to generate matrix $\varvec{Q}=({q}_{ij})$ by comparing $\varvec{P}$ and ${\varvec{P}}{\prime}$:

$$q_{ij}=I_{{p}_{ij}={p}_{ij}{\prime}}\oplus I_{{p}_{ij}=0}$$

where ${I}_{A}$ is the indicator function which equals 1 when $A$ is true. The entries in matrix $\varvec{Q}$ with value 1 are detected as local maxima or peaks.

### Cluster analysis

#### Boundary estimation by watershed

Considering each local maximum, we use a watershed-like approach to simulate the cluster boundary around it. We first create a density gradient with 100 levels ranging from 0 to the largest density in the 2D density matrix $\varvec{P}$. A set of contour lines which are polygons formed by grid points gradually change (shrink) over the density gradient. The change terminates when there is a contour in the set containing only the target local maximum, which results in our target contour line that contains only the corresponding maximum. All points surrounded by the contour line are then considered as the cluster members of the corresponding maximum. A cluster is not considered if it contains fewer than 100 members.

#### Silhouette analysis

We use the Silhouette Coefficient [55] to measure the clustering performance, which is directly calculated by intra-cluster distances and nearest-cluster distances. For a data point ${{\varvec{x}}}_{i}$ from cluster $A$, we define its intra-cluster distance by:

$$a(i)=\frac{1}{{S}_{A}-1}\sum_{i\ne j, j\in A}||{{{\varvec{x}}}_{i}-{{\varvec{x}}}_{j}||}_{2}$$

where ${S}_{A}$ is the size of cluster $A$. We also define its nearest-cluster distance by:

$$b(i)={min}_{B\ne A}\frac{1}{{S}_{B}}\sum_{j\in B}||{{{\varvec{x}}}_{i}-{{\varvec{x}}}_{j}||}_{2}$$

where ${S}_{B}$ is the size of cluster $B$. Then the silhouette of data points ${x}_{i}$ is written as:

$$s(i)=\frac{b(i)-a(i)}{max(a(i), b(i))}$$

The final Silhouette Coefficient is the mean silhouette of all clustered samples which ranges between − 1 and 1. Higher values indicate better clustering performance, or in other words the data points are more properly separated.

#### Contact frequency matrix construction

By selecting a certain number of neighbors around each peak, we are able to construct a contact frequency matrix for each peak. We estimate a path (polygon) surrounding each peak based on density. We then select all points inside the polygon as a cluster that corresponds to the peak. To calculate the contact frequency matrix ${\varvec{C}\varvec{M}}^{(a)}$ for structure $a$, we say beads $i$ and $j$ are in contact (i.e., ${c{m}_{ij}}^{(a)}=1$) if and only if:

$${\| {\varvec{x}}_{ia }-{\varvec{x}}_{ja}\| }_{2}\le 3{R}_{bead}$$

where ${\varvec{x}}_{ia}$ and ${\varvec{x}}_{ja}$ are the 3D coordinates of bead $i$ or bead $j$. ${R}_{bead}$ is the bead radius in our model. The contact frequency matrix for cluster $A$, ${\varvec{C}\varvec{M}}^{(A)},$ is calculated by the sum of all contact matrices in the cluster:

$${\varvec{C}\varvec{M}}^{(A)}=\sum_{a\epsilon A}{{\varvec{C}}{\varvec{M}}}^{(a)}$$

The contact frequency matrix for all structures that are classified to a cluster is calculated as:

$${\varvec{C}\varvec{M}}^{(Ens)}=\sum_{a\epsilon S}{{\varvec{C}}{\varvec{M}}}^{(a)}$$

where $S$ is the set of clustered structures. To enhance off-diagonal contacts, we visualize all contact frequency matrices from the models by applying transformation ${log}_{2}({cm}_{ij}+1)$. All color bars shown together with contact frequency matrices in the figures show a ratio with regard to the maximum value.

#### Average distance matrix construction

Similarly to the construction of a contact frequency matrix, we can also construct an average distance matrix for each cluster. Each entry ${dm}_{ij}^{(a)}$ of the distance matrix ${\varvec{D}\varvec{M}}^{(a)}$ for structure $a$ is calculated by the Euclidean distance between bead $i$ and bead $j$:

$${dm}_{ij}^{(a)}={\| {{\varvec{x}}}_{ia }-{{\varvec{x}}}_{ja}\| }_{2}$$

where ${{\varvec{x}}}_{ia}$ and ${{\varvec{x}}}_{ja}$ are the 3D coordinates of beads $i$ and $j$. ${dm}_{ij}^{(a)}$ is set to be 0 if the entry is at the diagonal. After min–max normalization of each distance matrix, the average matrix for cluster $A$ (which we will denote as ${\varvec{D}\varvec{M}}^{(A)}$) is calculated by the average of all matrices in the cluster:

$${\varvec{D}\varvec{M}}^{(A)}=\frac{1}{{S}_{A }}\sum_{a\epsilon A}{{\varvec{D}}{\varvec{M}}}^{(a)}$$

where ${S}_{A}$ is the number of structures in cluster $A$. All color bars shown together with average distance matrices in the figures show a ratio with regard to the maximum value.

#### Dissimilarity measurement

##### Euclidean distance dissimilarity

We first construct two flattened distance matrices ${\varvec{R}}^{\left(a\right)}$ and ${\varvec{R}}^{\left(b\right)}$ for structure $a$ and structure $b$. Each matrix contains Euclidean distances between all possible pairs of beads in each structure. The Euclidean distance between these two structures ${s}_{e}^{(ab)}$ is further calculated by:

$${s}_{e}^{(ab)}={\| {\varvec{R}}^{\left(a\right)}-{\varvec{R}}^{\left(b\right)}\| }_{2}$$

Then the final Euclidean distance dissimilarity between cluster $A$ and cluster $B$ is the average value of all possible pairs between these two clusters:

$${s}_{e}^{(AB)}=\frac{1}{M}\sum_{a\epsilon A, b\epsilon B}{s}_{e}^{(ab)}$$

where $$M$$ is the total number of pairs between the two clusters. To compare inter-cluster dissimilarity and intra-cluster dissimilarity, we normalize $${s}_{e}^{(AB)}$$ by the intra-cluster dissimilarity of cluster $$A$$$${s}_{e}^{(AA)}$$:

$$rs_{e}^{(AB)}=log_{2}\frac{S_{e}^{(AB)}}{S_{e}^{(AA)}}$$

##### Gaussian dissimilarity

The calculation of Gaussian dissimilarity is adapted from Eastwood and Wolynes [57] and Cheng et al. [37], which is an alternative way to compare pairwise distances between two structures. After generating ${d}_{ij}^{\left(a\right)}$ and ${d}_{ij}^{\left(b\right)}$ which are the Euclidean distances between bead $i$ and bead $j$ for both structure a and structure b, the Gaussian dissimilarity ${s}_{g}^{(ab)}$ is calculated by:

$${s}_{g}^{(ab)}=1-\frac{1}{N}\sum_{i<j}exp(-\frac{{({d}_{ij}^{\left(a\right)} -{ d}_{ij}^{\left(b\right)})}^{2}}{2{\sigma }^{2}})$$

where the scaling factor $\sigma =8{R}_{bead}$ and ${R}_{bead}$ is the bead radius in our model. $N$ is the total number of pairs of beads in the structure. Similarly, the final Gaussian dissimilarity between cluster $A$ and cluster $B$ is the average value of all possible pairs between these two clusters:

$${s}_{g}^{(AB)}=\frac{1}{M}\sum_{a\epsilon A, b\epsilon B}{s}_{g}^{(ab)}$$

where $$M$$ is the total number of pairs between the two clusters. To compare inter-cluster dissimilarity with intra-cluster dissimilarity, we normalize $${s}_{g}^{(AB)}$$ by the intra-cluster dissimilarity of cluster $$A$$$${s}_{g}^{(AA)}$$:

$${rs}_{g}^{(AB)}={log}_{2}\frac{{s}_{g}^{(AB)}}{{s}_{g}^{(AA)}}$$

Due to computational complexity, we randomly select 200 structures from each cluster to compute the similarities above.

##### Wasserstein distance dissimilarity

We calculate both intra-cluster dissimilarity and inter-cluster dissimilarity by distance measurement to compare low intra-cluster dissimilarity with high inter-cluster dissimilarity. The Wasserstein distance $W\left(u, v\right)$ measures the dissimilarity between two probability distributions $u$ and $v$ by:

$$W\left(u, v\right)={\int }_{-\infty }^{+\infty }\left|U-V\right|ds$$

where $U$ and $V$ are the cumulative probability distributions of $u$ and $v$ [56]. To measure dissimilarity between two clusters of structures, for each pair of bead $i$ and bead $j$, we obtain the 1D probability distributions for the distances between pair $i$ and $j$ in cluster A $d_{ij}^{(A)}$ and the distances between pair $i$ and $j$ in cluster B $d_{ij}^{(B)}$, which are then used to calculate the Wasserstein distance of these two distributions. The final dissimilarity ${s}_{w}^{(AB)}$ is obtained by averaging the Wasserstein distances of all possible pairs:

$${s}_{w}^{(AB)}=\frac{1}{N}\sum_{i<j}W({d}_{ij}^{\left(A\right)}, {d}_{ij}^{\left(B\right)})$$

where $$N$$ is the total number of pairs of beads in the structure. For intra-cluster dissimilarity, we randomly sample a subcluster with size $$\frac{M}{2}$$ and indices $$i$$ which is then used to calculate the final dissimilarity with its reverse subcluster with indices $$(M-i-1)$$, where $$M$$ is the number of structures in cluster $$A$$. For inter-cluster dissimilarity, we directly apply the method above. To compare inter-cluster dissimilarity with intra-cluster dissimilarity, we normalize $${s}_{w}^{(AB)}$$ by the intra-cluster dissimilarity of cluster $$A$$$${s}_{w}^{(AA)}$$:

$${rs}_{w}^{(AB)}={log}_{2}\frac{{s}_{w}^{(AB)}}{{s}_{w}^{(AA)}}$$

#### Proximity frequency map

The calculation of a proximity frequency map is similar to the calculation of a contact frequency matrix. We select a larger range to visualize inter-chromosomal contact patterns. To calculate the proximity frequency map ${\varvec{P}\varvec{M}}^{(a)}$ for structure $a$, we define the $i$ th bead and the $j$ th bead in structure $a$ forms a contact (i.e., ${p{m}_{ij}}^{(a)}=1$) if and only if:

$${\| {{\varvec{x}}}_{ia}-{{\varvec{x}}}_{ja}\| }_{2}\le {R}_{soft}$$

where $${{\varvec{x}}}_{ia}$$ or $${{\varvec{x}}}_{ja}$$ are the 3D coordinates of bead $$i$$ or bead $$j$$. We set $${R}_{soft}=\text{2,000} nm$$. The proximity frequency map for cluster $$A$$$${\varvec{P}\varvec{M}}^{(A)}$$ is calculated as:

$${\varvec{P}\varvec{M}}^{(A)}=\frac{1}{{S}_{A }}\sum_{a\epsilon A}{{\varvec{P}}{\varvec{M}}}^{(a)}$$

where ${S}_{A}$ is the number of structures in cluster $A$. The inter-chromosomal parts of each map are shown as the average of both homologous copies, while the intra-chromosomal part is calculated from the target chromosome copy only.

### Structural features prediction

#### Insulation score

##### Contact-based approach

We use sliding windows to slide along the diagonal of contact frequency matrix ${\varvec{C}\varvec{M}}^{(A)}$ to calculate contact differences as the insulation score profile for cluster $A$. For chromatin region $i$, we use upper triangular matrix ${\varvec{L}\varvec{M}}_{i}^{(A)}$ representing the binary submatrix of the left region with length $l$, upper triangular matrix ${\varvec{R}\varvec{M}}_{i}^{(A)}$ representing the binary submatrix of the right region with length $l$ (including region $i$) and matrix ${\varvec{M}}_{i}^{(A)}$ representing the binary submatrix between the left region with length $l$ and the right region with length $l$ (including region $i$) to calculate the score:

$${is}_{i}^{(A)}={\langle \varvec{E}, {\varvec{L}\varvec{M}}_{i}^{(A)}\rangle }_{F}+{\langle \varvec{E}, {\varvec{R}\varvec{M}}_{i}^{(A)}\rangle }_{F}-{\langle \varvec{E}, {\varvec{M}}_{i}^{(A)}\rangle }_{F}$$

where $l$ is the sliding window size. ${\langle \varvec{X}, \varvec{Y}\rangle }_{F}$ is the Frobenius inner product between matrix $\varvec{X}$ and matrix $\varvec{Y}$. $\varvec{E}$ is a matrix of ones. The largest peaks of the insulation score profile are used as candidates for domain boundaries.

##### Distance matrix approach

The insulation score profile can also be determined using the distance matrix ${\varvec{D}\varvec{M}}^{(A)}$. To calculate the score, we also use the upper triangular matrix ${\varvec{L}\varvec{M}}_{i}^{(A)}$, upper triangular matrix ${\varvec{R}\varvec{M}}_{i}^{(A)}$, and matrix ${\varvec{M}}_{i}^{(A)}$ in the same way as described above except we use the average distance matrix for each cluster. We first transform the distance matrix into a binary matrix and set the entry to be 1 if its value is larger than the threshold (0.4 to 0.5). Then the score for chromatin region $i$ in a cluster is defined by:

$${is}_{i}^{(A)}={{\langle \varvec{E}, {\varvec{M}}_{i}^{(A)}\rangle }_{F}-\langle \varvec{E}, {\varvec{L}\varvec{M}}_{i}^{(A)}\rangle }_{F}-{\langle \varvec{E}, {\varvec{R}\varvec{M}}_{i}^{(A)}\rangle }_{F}$$

To accurately detect the peak locations, we first use a larger window size (40 MB) to detect approximate locations of domain boundaries in each cluster, then we use a smaller window size (6 Mb) to detect their specific locations. When detecting peaks in the profile, we use detecta https://github.com/demotu/detecta to calculate the locations of local maximas.

#### Radial position (RAD)

The radial position of a chromatin region $i$ in structure $s$ in a spherical nucleus (as GM12878) is calculated as:

$${r}_{i}^{(s)}=\frac{{\| {{\varvec{x}}}_{is}\| }_{2}}{{R}_{nuc}}$$

where ${{\varvec{x}}}_{is}$ is the the 3D coordinates of bead $i$ in structure s, and ${R}_{nuc}$ is the nucleus radius which is 5 μm. ${r}_{i}^{(s)}=0$ means the region $i$ is at the nuclear center while ${r}_{i}^{(s)}=1$ means it is located at the nuclear surface. The average radial position (RAD) of cluster $A$ is the average of radial positions of all structures in this cluster:

$${r}_{i}^{(A)}=\frac{1}{{S}_{A}}\sum_{a\in A}{r}_{i}^{(a)}$$

where ${S}_{A}$ is the number of structures in cluster $A$. To compare against the ensemble profile, we use a log ratio comparison to show the difference. The log ratio of cluster $A$ radial position against the ensemble one (RadRatio) is calculated as:

$${rr}_{i}^{(A)}={log}_{2}\frac{{r}_{i}^{(A)}}{{r}_{i}^{(Ens)}}$$

where the ensemble radial position is calculated in the same way, but for all structures that are classified to any cluster. Similarly, we can calculate all following structural features with the $(Ens)$ superscript.

#### Radius of gyration (RG) (i.e., local chromatin fiber decompaction)

The local compaction of the chromatin fiber at the location of a given locus is estimated by the radius of gyration for a 1-Mb region centered at the locus. To estimate the values along an entire chromosome, we use a sliding window approach over all chromatin regions in a chromosome. The radius of gyration for a 1-Mb region centered at locus $i$ in structure $a$, is calculated as:

$${rg}_{i}^{(a)}=\sqrt{\frac{1}{5}{\sum }_{j=1}^{5}{d}_{j}^{2}}$$

where ${d}_{j}$ is the distance between the chromatin region $j$ to the center of mass of the 1-Mb region. The average radius of gyration (RG) of cluster $A$ is the average of radial positions of all structures in this cluster:

$${rg}_{i}^{(A)}=\frac{1}{{S}_{A}}\sum_{a\in A}{rg}_{i}^{(a)}$$

where ${S}_{A}$ is the number of structures in cluster $A$. Similarly, the log ratio of cluster $A$ radius of gyration against the ensemble one (RgRatio) is calculated as:

$${rrg}_{i}^{(A)}={log}_{2}\frac{{rg}_{i}^{(A)}}{{rg}_{i}^{(Ens)}}$$

For the overall compactness of the conformation, we use all the beads to calculate the radius of gyration for structure $a$:

$${rg}^{(a)}=\sqrt{\frac{1}{N}{\sum }_{j=1}^{N}{d}_{j}^{2}}$$

where $N$ is the total number of beads in the structure, ${d}_{j}$ is the distance between the chromatin region $j$ to the center of mass of the whole structure.

#### Structural variability (δRAD)

The structural variability (δRAD) of region $i$ in cluster $A$ is calculated as:

$${sv}_{i}^{(A)}={log}_{2}\frac{{\sigma }_{i}^{(A)}}{\overline{{\sigma }^{(A)}}}$$

where ${\sigma }_{i}^{(A)}$ is the standard deviation of the population of radial positions of region $i$ in cluster $A$ and $\overline{{\sigma }^{(A)}}$ is the mean standard deviation calculated from all regions within the same chromosome of the target region. Positive values (${sv}_{i}^{(A)}>0$) result from high cell-to-cell variability of radial position, whereas negative values (${sv}_{i}^{(A)}<0$) indicate low variability. The log ratio of cluster $A$ structural variability against the ensemble one (δRadRatio) is calculated as:

$${rsv}_{i}^{(A)}={log}_{2}\frac{{sv}_{i}^{(A)}}{{sv}_{i}^{(Ens)}}$$

#### Prediction of speckle locations in single cells

We follow the approach by Yildirim et al. [19] and Boninsegna et al. [17] to calculate the locations of nuclear speckles in single cells of GM12878. For each single cell, a chromatin interaction network is calculated for chromatin regions that are part of the A1 subcompartment as defined in Rao et al. [31], where each vertex represents a 200-kb chromatin region and edge is created between two vertices $i$, $j$ if the corresponding chromatin regions has a spatial distance ${d}_{ij}\le 4{R}_{bead}$, where ${R}_{bead}$ is the bead radius in our model.

We then apply the Markov Clustering Algorithm (MCL) [74] to detect highly connected subgraphs within a network using the MCL tool in the MCL-edge software [74]. Speckle locations are identified as the geometric center of each detected A1 subgraph in each cell. In each structure, A1 subgraphs are only considered with sizes larger than 3 nodes. This procedure predicts locations of speckles, which reproduce with high correlation experimental SON TSA-seq data [19].

#### Speckle distance (SpD)

The speckle distance (SpD) for region $i$ is calculated by measuring the distance between the surface of each chromatin region $i$ to the nearest speckle:

$${sd}_{i}^{(A)}=\frac{1}{{S}_{A}}\sum_{a\epsilon A}{d}_{il}^{(a)}$$

where ${S}_{A}$ is the number of structures in cluster $A$, ${d}_{il}^{(a)}$ is the distance between the region $i$ and the predicted nearest nuclear body location $l$. The log ratio of cluster $A$ speckle distance against the ensemble one (SpdRatio) is calculated as:

$${rsd}_{i}^{(A)}={log}_{2}\frac{{sd}_{i}^{(A)}}{{sd}_{i}^{(Ens)}}$$

#### Speckle TSA-seq (SON TSA-seq)

Speckle TSA-seq can be viewed as an average over distances to all speckles. To predict TSA-seq signals for speckle from our models, we use the following equation:

$${sg}_{i}^{(A)}=\frac{1}{{S}_{A}}\sum_{a\epsilon A}{\sum }_{l=1}^{L}{e}^{-k{d}_{il}^{(a)}}$$

where ${S}_{A}$ is the number of structures in cluster $A$, $L$ is the number of predicted speckle locations in structure $a$, ${d}_{il}^{(a)}$ is the distance between the region $i$ and the predicted nuclear body location $l$, and $k$ is the estimated decay constant in the TSA-seq experiment [49] which is set to 4 in our calculations. The normalized TSA-seq signal for region $i$ then becomes:

$${ts}_{i}^{(A)}={log}_{2}\frac{{sg}_{i}^{(A)}}{\overline{{sg }^{(A)}}}$$

where $\overline{{sg }^{(A)}}$ is the mean signal calculated from all regions in the genome. The predicted speckles are used for distance calculations. The log ratio of cluster $A$ speckle TSA-seq against the ensemble one (SON TSA-seq Ratio) is calculated as:

$${rts}_{i}^{(A)}={log}_{2}\frac{{ts}_{i}^{(A)}}{{ts}_{i}^{(Ens)}}$$

#### Speckle association frequency (SAF)

For a given 200-kb region, the association frequency to the speckle (SAF) is calculated as:

$${saf}_{i}^{(A)}=\frac{{n}_{{d}_{i}<{d}_{t}}}{{S}_{A}}$$

where ${S}_{A}$ is the number of structures in cluster $A$, ${n}_{{d}_{i}<{d}_{t}}$ is the number of structures, in which region $i$ have a distance to the speckle smaller than the association threshold ${d}_{t}$. We set ${d}_{t}$ to be 1000 nm for the model and 500 nm for the DNA-MERFISH dataset [6]. For SAF calculation, we use the predicted speckle to calculate distances (see Identifying spatial partitions by Markov clustering), where we calculate distances from the surface of the region to the center-of-mass of the partition. The log ratio of cluster $A$ SAF against the ensemble one (SafRatio) is calculated as:

$${rsaf}_{i}^{(A)}={log}_{2}\frac{{saf}_{i}^{(A)}}{{saf}_{i}^{(Ens)}}$$

#### Inter-chromosomal proximity profile (IPP)

The calculation of inter-chromosomal proximity profile (IPP) is based on the proximity frequency map. For a given 200-kb region, the process is similar to the calculation of speckle association frequency, but we replace the distance to the smallest speckle by the contact with any inter-chromosomal regions which can be chromosome-wide or genome-wide:

$${ipp}_{i}^{(A)}=\frac{{n}_{{d}_{i}\le {R}_{soft}}}{{S}_{A}}$$

where ${S}_{A}$ is the number of structures in cluster $A$, ${n}_{{d}_{i}\le {R}_{soft}}$ is the total number of contacts, in which region $i$ is within contact range ${R}_{soft}=\text{2,000} nm$ with any target inter-chromosomal regions from the same genome structure. Every IPP is shown as the average of both homologous copies. The log ratio of cluster $A$ IPP against the ensemble one (IppRatio) is calculated as:

$${ripp}_{i}^{(A)}={log}_{2}\frac{{ipp}_{i}^{(A)}}{{ipp}_{i}^{(Ens)}}$$

When calculating the average IppRatio of a chromosome, we calculate the mean of chromosome-wide IppRatios of the chromosome.

#### Histone modification signals, reference genes, and synteny blocks

We collected histone modification signals including H3K27ac, H3K4me1, H3K4me3, H3K9me3, H3K27me3, and H4K20me1 for GM12878 and H3K9me2 for GM23338 from the ENCODE [75]. The reference genes file for hg38 was downloaded from the UCSC Genome Browser [76]. The genes are mapped to the 200-kb bins according to their transcription midpoints. All related signals and genes together with other structural features are shown by the Integrative Genomics Viewer (IGV) [77]. The subcompartments for GM12878 are obtained from Rao et al. [31]. The genetic information and illustration of synteny blocks for both human and mouse chromosomes are generated by Synteny Portal [78].

### Cluster assessment with experimental single-cell data

#### Single cell Hi-C assessment

##### Sci-HiC dataset

We collected multiple sci-HiC datasets of GM12878 from the 4DN data portal (4DNESUE2NSGS) [13]. Each dataset consists of single-cell sequencing data of thousands of cells and we collected more than 11,000 single cells in total. A systematic way of massively demultiplexing single cell Hi-C is discussed in Ramani et al. [13] which applies combinatorial cellular indexing to chromosome conformation capture. We use the provided pipeline to process all collected sci-HiC datasets. Due to the large number of missing contacts, it is necessary for us to preprocess the datasets to reconstruct missing information. We adapt the preprocessing method from Zhou et al. [58]. Given a raw single-cell contact matrix ${{\varvec{M}}}^{raw}={({m}_{ij}^{raw})}_{n\times n}$, we construct a new matrix ${\varvec{M}}^{conv}={({m}_{ij}^{conv})}_{n\times n}$ by applying convolution with filter ${\varvec{F}=({f}_{ij})}_{(2w+1)\times (2w+1)}$:

$${m}_{ij}^{conv}=\sum_{k, l}{f}_{kl}{m}_{kl}^{raw}$$

For a 200-kb matrix, we set $w=5$. In this step, we integrate the interaction information from the genomic neighbors to impute the interaction at each position. Random walk with restart is then performed to estimate contact probability between every two beads. In order to perform a random walk, a transition matrix ${\varvec{M}}^{trans}={({m}_{ij}^{trans})}_{n\times n}$ is calculated based on the contact matrix after convolution. Every entry in the original matrix is normalized by its corresponding row sum:

$${m}_{ij}^{trans}=\frac{{m}_{ij}^{conv}}{{\sum }_{j{\prime}}{m}_{ij{\prime}}^{conv}}$$

We initialize the random walk by an identity matrix ${\varvec{R}}_{0}$ so that the contact probability between every two beads is set to be 0. By applying the following recurrence formula, we are able to obtain a resulting matrix after the values converge:

$${\varvec{R}}_{0}=\varvec{I}$$

$${\varvec{R}}_{t}=\left(1-p\right){\varvec{R}}_{t-1}{{\varvec{M}}}^{trans}+p\varvec{I}$$

where $\varvec{I}$ is the identity matrix and *p* is the restart probability with 0.5. We define there is a convergence when ${\| {\varvec{R}}_{t}-{\varvec{R}}_{t-1}\| }_{2}\le {10}^{-6}$. Each element in the resulting matrix ${\varvec{R}}_{t}$ after convergence indicates the probability of the random walk to reach the *j*th node when starting from the *i*th node. All contacts with probability larger than the 75th percentile of all probabilities in each row are chosen to convert ${\varvec{R}}_{t}$ into a binary matrix ${\varvec{M}}^{rw}$.

##### Sci-HiC assessment

For comparison of clusters, a direct way is to compare their contact frequency matrices. We define the difference matrix of cluster $A$ to be:

$${d}_{ij}^{(A)}={log}_{2}\frac{{S}_{A}{m}_{ij}^{(Pop)}}{{S}_{Pop}{m}_{ij}^{(A)} }$$

where ${\varvec{D}}^{(A)}=({d}_{ij}^{(A)})$ is the resulting difference matrix. ${\varvec{M}}^{(Pop)}=({m}_{ij}^{(Pop)})$ is the contact frequency matrix for the whole population calculated by contact range 2 (contact when two beads are within the range of twice the bead radius), while ${\varvec{M}}^{(A)}=({m}_{ij}^{(A)})$ is the contact frequency matrix for the cluster. ${S}_{A}$ is the cluster size while ${S}_{Pop}$ is the population size. Due to the sparsity of single-cell Hi-C, we preprocess each raw contact matrix by the preprocessing method above to construct a processed contact matrix. The next step is to assign each contact matrix to the clusters defined by our model. For cluster $A$, a superiority mask ${\varvec{M}}_{sup}^{(A)}=({ms}_{ij}^{(A)})$ and an inferiority mask ${\varvec{M}}_{inf}^{(A)}=({mi}_{ij}^{(A)})$ are calculated by its difference matrix ${\varvec{D}}^{(A)}$:

$${ms}_{ij}^{(A)}={I}_{{d}_{ij}^{(A)}\ge 5}$$

$${mi}_{ij}^{(A)}={I}_{{d}_{ij}^{(A)}\le -1}$$

where ${I}_{A}$ is the indicator function which equals 1 when $A$ is true. For each preprocessed matrix ${\varvec{M}}^{rw}$ from the sci-HiC population, we define the assessment score as:

$${s}^{(A)}=exp(\frac{{\langle {\varvec{M}}^{rw}, { \varvec{M}}_{inf}^{(A)}\rangle }_{F}}{{\langle \varvec{E}, {\varvec{M}}_{inf}^{(A)}\rangle }_{F}}-\frac{{\langle {\varvec{M}}^{rw}, {\varvec{M}}_{sup}^{(A)}\rangle }_{F}}{{\langle \varvec{E}, {\varvec{M}}_{sup}^{(A)}\rangle }_{F}})$$

where ${\langle \varvec{X}, \varvec{Y}\rangle }_{F}$ is the Frobenius inner product between matrix $\varvec{X}$ and matrix $\varvec{Y}$. $\varvec{E}$ is a matrix of ones. For each contact matrix, we choose the pair of masks that has the largest assessment score with the matrix and assign the matrix to the corresponding cluster. To filter matrices that are different from all clusters, we only classify matrices to a cluster ${A}_{1}$ when ${s}^{\left({A}_{1}\right)}-{s}^{\left({A}_{2}\right)}\ge 0.01$, where ${A}_{1}$ is the cluster with the largest matching score and ${A}_{2}$ is the second largest one. The final matching probability is defined as:

$${p}^{({A}_{1})}=\frac{{s}^{({A}_{1})}}{{\sum }_{k=1}^{K}{s}^{({A}_{k})}}$$

where $K$ is the total number of clusters. Similarly, a contact frequency matrix can be generated using all inferred sci-HiC matrices classified to each cluster. To preserve symmetry, we symmetrize each contact frequency matrix by selecting the minimum number of contacts between pairs $(i, j)$ and $(j, i)$.

##### Sci-HiC control dataset

A control dataset is generated to ensure our assessment procedure is not classifying artifacts and false signals. For each sci-HiC contact matrix, we randomly rearrange all the entries while maintaining its diagonality and the total number of contacts to construct a sudo single-cell contact matrix. We apply this process for every sci-HiC matrix and construct a control dataset in the end. The same assessment procedure is then applied to the control dataset.

#### Imaging assessment

##### DNA-MERFISH dataset

We process the DNA-MERFISH datasets from Su et al. [6] which includes high-resolution coordinates of chromosome 2 from 3029 copies and low-resolution coordinates of chromosome 6 from 7336 copies. For chromosome 6, we also process distances of imaged genomic regions to the nearest detected speckle and total numbers of transcription “on” of all associated genes for each locus. All datasets are preprocessed by linear interpolation to remove missing values if applicable. We remove copies without valid values in coordinates and in speckle distances.

##### DNA-MERFISH assessment

The preprocessed DNA-MERFISH coordinates can be then used for assessment. Each single structure is used to calculate a distance matrix $\varvec{D}\varvec{M}$ in the same way stated above. To compare $\varvec{D}\varvec{M}$ with the average distance matrix ${\varvec{D}\varvec{M}}^{(A)}$ for cluster $A$, we first downsample ${\varvec{D}\varvec{M}}^{(A)}$ by selecting the beads that are mapped by the DNA-MERFISH coordinates. Then we flatten both matrices by extracting the upper triangular parts and normalizing them by min–max normalization to generate two distance vectors $\varvec{R}$ and ${\varvec{R}}^{(A)}$. We define the assessment score as:

$${s}^{(A)}=exp(r(\varvec{R}, {\varvec{R}}^{\left(A\right)}))$$

where $r(\varvec{x}, \varvec{y})$ measures the Pearson’s correlation coefficient between vector $\varvec{x}$ and vector $\varvec{y}$. For each distance matrix, we choose the average distance matrix that has the largest assessment score with the matrix and assign the matrix to the corresponding cluster if they are sufficiently similar. To filter matrices that are different from all clusters, we only classify matrices to a cluster ${A}_{1}$ when ${s}^{({A}_{1})}-{s}^{({A}_{2})}\ge 0.05$, where ${A}_{1}$ is the cluster with the largest matching score and ${A}_{2}$ is the cluster the second largest one. The final matching probability is defined as:

$${p}^{({A}_{1})}=\frac{{s}^{({A}_{1})}}{{\sum }_{k=1}^{K}{s}^{({A}_{k})}}$$

where $K$ is the total number of clusters. Similarly, an average distance matrix can be generated using all DNA-MERFISH distance matrices classified to each cluster.

### Data visualization

All chromosome structures are visualized by Chimera [79].

## Supplementary Information

Supplementary Material 1.

Supplementary Material 2.

## Data Availability

The Hi-C datasets for H1-hESC and HFFc6 are obtained from 4DN Data Portal with accession code 4DNES2R6PUEK [80] and accession code 4DNES2M5JIGV [81]. The sci-HiC dataset is obtained from 4DN Data Portal with accession code 4DNESUE2NSGS [82]. The DNA-MERFISH dataset is available at https://doi.org/10.5281/zenodo.3928890 [83]. The genome population of cell line GM12878 obtained from Yildirim et al. [19] is available at https://doi.org/10.5281/zenodo.7352276 [84]. The genome populations of cell lines H1-hESC and HFFc6 are available at https://doi.org/10.5281/zenodo.7563402 [85]. The Integrative Genome Modeling (IGM) software used to generate the genome populations is available at https://github.com/alberlab/igm [86]. The scripts used to perform embedding and clustering analysis are available at https://github.com/alberlab/Conformation_Analysis [87] together with the Zenodo repository https://doi.org/10.5281/zenodo.14538117 [88]. All codes are open source under the GNU General Public License v3.0 (GPL-3.0).

## References

[1] Nguyen HQ, Chattoraj S, Castillo D, Nguyen SC, Nir G, Lioutas A, et al. 3D mapping and accelerated super-resolution imaging of the human genome using in situ sequencing. Nat Methods. 2020;17:822–32. 32719531 10.1038/s41592-020-0890-0 PMC7537785

[2] Bintu B, Mateo LJ, Su J-H, Sinnott-Armstrong NA, Parker M, Kinrot S, et al. Super-resolution chromatin tracing reveals domains and cooperative interactions in single cells. Science. 2018;362: eaau1783. 30361340 10.1126/science.aau1783 PMC6535145

[3] Mateo LJ, Murphy SE, Hafner A, Cinquini IS, Walker CA, Boettiger AN. Visualizing DNA folding and RNA in embryos at single-cell resolution. Nature. 2019;568:49–54. 30886393 10.1038/s41586-019-1035-4 PMC6556380

[4] Cardozo Gizzi AM, Cattoni DI, Fiche J-B, Espinola SM, Gurgo J, Messina O, et al. Microscopy-based chromosome conformation capture enables simultaneous visualization of genome organization and transcription in intact organisms. Mol Cell. 2019;74:212-222.e5. 30795893 10.1016/j.molcel.2019.01.011

[5] Liu M, Lu Y, Yang B, Chen Y, Radda JSD, Hu M, et al. Multiplexed imaging of nucleome architectures in single cells of mammalian tissue. Nat Commun. 2020;11:2907. 32518300 10.1038/s41467-020-16732-5 PMC7283333

[6] Su J-H, Zheng P, Kinrot SS, Bintu B, Zhuang X. Genome-scale imaging of the 3D organization and transcriptional activity of chromatin. Cell. 2020;182:1641-1659.e26. 32822575 10.1016/j.cell.2020.07.032 PMC7851072

[7] Eng C-HL, Lawson M, Zhu Q, Dries R, Koulena N, Takei Y, et al. Transcriptome-scale super-resolved imaging in tissues by RNA seqFISH. Nature. 2019;568:235–9. 30911168 10.1038/s41586-019-1049-y PMC6544023

[8] Payne AC, Chiang ZD, Reginato PL, Mangiameli SM, Murray EM, Yao C-C, et al. In situ genome sequencing resolves DNA sequence and structure in intact biological samples. Science. 2021;371: eaay3446. 33384301 10.1126/science.aay3446 PMC7962746

[9] Takei Y, Yun J, Zheng S, Ollikainen N, Pierson N, White J, et al. Integrated spatial genomics reveals global architecture of single nuclei. Nature. 2021;590:344–50. 33505024 10.1038/s41586-020-03126-2 PMC7878433

[10] Takei Y, Zheng S, Yun J, Shah S, Pierson N, White J, et al. Single-cell nuclear architecture across cell types in the mouse brain. Science. 2021;374:586–94. 34591592 10.1126/science.abj1966

[11] Takei Y, Yang Y, White J, Yun J, Prasad M, Ombelets LJ, et al. High-resolution spatial multi-omics reveals cell-type specific nuclear compartments. Genomics; 2023. Available from: http://biorxiv.org/lookup/doi/10.1101/2023.05.07.539762. 10.1038/s41586-025-08838-x 40205045

[12] Nagano T, Lubling Y, Stevens TJ, Schoenfelder S, Yaffe E, Dean W, et al. Single-cell Hi-C reveals cell-to-cell variability in chromosome structure. Nature. 2013;502:59–64. 24067610 10.1038/nature12593 PMC3869051

[13] Ramani V, Deng X, Qiu R, Gunderson KL, Steemers FJ, Disteche CM, et al. Massively multiplex single-cell Hi-C. Nat Methods. 2017;14:263–6. 28135255 10.1038/nmeth.4155 PMC5330809

[14] Tan L, Xing D, Chang C-H, Li H, Xie XS. Three-dimensional genome structures of single diploid human cells. Science. 2018;361:924–8. 30166492 10.1126/science.aat5641 PMC6360088

[15] Li G, Liu Y, Zhang Y, Kubo N, Yu M, Fang R, et al. Joint profiling of DNA methylation and chromatin architecture in single cells. Nat Methods. 2019;16:991–3. 31384045 10.1038/s41592-019-0502-z PMC6765429

[16] Lee D-S, Luo C, Zhou J, Chandran S, Rivkin A, Bartlett A, et al. Simultaneous profiling of 3D genome structure and DNA methylation in single human cells. Nat Methods. 2019;16:999–1006. 31501549 10.1038/s41592-019-0547-z PMC6765423

[17] Boninsegna L, Yildirim A, Polles G, Zhan Y, Quinodoz SA, Finn EH, et al. Integrative genome modeling platform reveals essentiality of rare contact events in 3D genome organizations. Nat Methods. 2022;19:938–49. 35817938 10.1038/s41592-022-01527-x PMC9349046

[18] Boninsegna L, Yildirim A, Zhan Y, Alber F. Integrative approaches in genome structure analysis. Structure. 2022;30:24–36. 34963059 10.1016/j.str.2021.12.003 PMC8959402

[19] Yildirim A, Hua N, Boninsegna L, Zhan Y, Polles G, Gong K, et al. Evaluating the role of the nuclear microenvironment in gene function by population-based modeling. Nat Struct Mol Biol. 2023;30:1193–206. 37580627 10.1038/s41594-023-01036-1 PMC10442234

[20] Yildirim A, Boninsegna L, Zhan Y, Alber F. Uncovering the principles of genome folding by 3D chromatin modeling. Cold Spring Harb Perspect Biol. 2021;14:a039693. 10.1101/cshperspect.a039693 PMC9248826 34400556

[21] Di Pierro M, Zhang B, Aiden EL, Wolynes PG, Onuchic JN. Transferable model for chromosome architecture. Proc Natl Acad Sci USA. 2016;113:12168–73. 27688758 10.1073/pnas.1613607113 PMC5087044

[22] Qi Y, Zhang B. Polymer modeling of whole-nucleus diploid genome organization. Biophys J. 2020;118:550a–550a.

[23] Mendieta-Esteban J, Di Stefano M, Castillo D, Farabella I, Marti-Renom MA. 3D reconstruction of genomic regions from sparse interaction data. NAR Genom Bioinform. 2021;3:lqab017. 33778492 10.1093/nargab/lqab017 PMC7985034

[24] Krietenstein N, Abraham S, Venev SV, Abdennur N, Gibcus J, Hsieh THS, et al. Ultrastructural details of mammalian chromosome architecture. Mol Cell. 2020;78:554-565.e7. 32213324 10.1016/j.molcel.2020.03.003 PMC7222625

[25] Conte M, Fiorillo L, Bianco S, Chiariello AM, Esposito A, Nicodemi M. Polymer physics indicates chromatin folding variability across single-cells results from state degeneracy in phase separation. Nat Commun. 2020;11:3289. 32620890 10.1038/s41467-020-17141-4 PMC7335158

[26] Paulsen J, Gramstad O, Collas P. Manifold based optimization for single-cell 3D Genome Reconstruction. PLoS Comput Biol. 2015;11: e1004396. 26262780 10.1371/journal.pcbi.1004396 PMC4532452

[27] Carstens S, Nilges M, Habeck M. Inferential structure determination of chromosomes from single-cell Hi-C data. PLoS Comput Biol. 2016;12: e1005292. 28027298 10.1371/journal.pcbi.1005292 PMC5226817

[28] Giorgetti L, Galupa R, Nora EP, Piolot T, Lam F, Dekker J, et al. Predictive polymer modeling reveals coupled fluctuations in chromosome conformation and transcription. Cell. 2014;157:950–63. 24813616 10.1016/j.cell.2014.03.025 PMC4427251

[29] Misteli T. The self-organizing genome: principles of genome architecture and function. Cell. 2020;183:28–45. 32976797 10.1016/j.cell.2020.09.014 PMC7541718

[30] Kempfer R, Pombo A. Methods for mapping 3D chromosome architecture. Nat Rev Genet. 2020;21:207–26. 31848476 10.1038/s41576-019-0195-2

[31] Rao SSP, Huntley MH, Durand NC, Stamenova EK, Bochkov ID, Robinson JT, et al. A 3D map of the human genome at kilobase resolution reveals principles of chromatin looping. Cell. 2014;159:1665–80. 25497547 10.1016/j.cell.2014.11.021 PMC5635824

[32] Akgol Oksuz B, Yang L, Abraham S, Venev SV, Krietenstein N, Parsi KM, et al. Systematic evaluation of chromosome conformation capture assays. Nat Methods. 2021;18:1046–55. 34480151 10.1038/s41592-021-01248-7 PMC8446342

[33] Zhang R, Zhou T, Ma J. Multiscale and integrative single-cell Hi-C analysis with Higashi. Nat Biotechnol. 2022;40:254–61. 34635838 10.1038/s41587-021-01034-y PMC8843812

[34] Gabriele M, Brandão HB, Grosse-Holz S, Jha A, Dailey GM, Cattoglio C, et al. Dynamics of CTCF- and cohesin-mediated chromatin looping revealed by live-cell imaging. Science. 2022;376:496–501. 35420890 10.1126/science.abn6583 PMC9069445

[35] Fudenberg G, Imakaev M, Lu C, Goloborodko A, Abdennur N, Mirny LA. Formation of chromosomal domains by loop extrusion. Cell Rep. 2016;15:2038–49. 27210764 10.1016/j.celrep.2016.04.085 PMC4889513

[36] Sanborn AL, Rao SSP, Huang S-C, Durand NC, Huntley MH, Jewett AI, et al. Chromatin extrusion explains key features of loop and domain formation in wild-type and engineered genomes. Proc Natl Acad Sci U S A. 2015;112:E6456-6465. 26499245 10.1073/pnas.1518552112 PMC4664323

[37] Cheng RR, Contessoto VG, Lieberman Aiden E, Wolynes PG, Di Pierro M, Onuchic JN. Exploring chromosomal structural heterogeneity across multiple cell lines. Elife. 2020;9: e60312. 33047670 10.7554/eLife.60312 PMC7593087

[38] Finn EH, Misteli T. Molecular basis and biological function of variability in spatial genome organization. Science. 2019;365:eaaw9498. 31488662 10.1126/science.aaw9498 PMC7421438

[39] Finn EH, Pegoraro G, Brandão HB, Valton A-L, Oomen ME, Dekker J, et al. Extensive heterogeneity and intrinsic variation in spatial genome organization. Cell. 2019;176:1502-1515.e10. 30799036 10.1016/j.cell.2019.01.020 PMC6408223

[40] Sawh AN, Shafer MER, Su JH, Zhuang X, Wang S, Mango SE. Lamina-dependent stretching and unconventional chromosome compartments in early C. elegans Embryos. Mol Cell. 2020;78:96-111.e6. 32105612 10.1016/j.molcel.2020.02.006 PMC7263362

[41] Götz M, Messina O, Espinola S, Fiche J-B, Nollmann M. Multiple parameters shape the 3D chromatin structure of single nuclei at the doc locus in Drosophila. Nat Commun. 2022;13:5375. 36104317 10.1038/s41467-022-32973-y PMC9474875

[42] Dekker J, Belmont AS, Guttman M, Leshyk VO, Lis JT, Lomvardas S, et al. The 4D nucleome project. Nature. 2017;549:219–26. 28905911 10.1038/nature23884 PMC5617335

[43] Reiff SB, Schroeder AJ, Kırlı K, Cosolo A, Bakker C, Lee S, et al. The 4D nucleome data portal as a resource for searching and visualizing curated nucleomics data. Nat Commun. 2022;13:2365. 35501320 10.1038/s41467-022-29697-4 PMC9061818

[44] Barbieri M, Chotalia M, Fraser J, Lavitas L-M, Dostie J, Pombo A, et al. Complexity of chromatin folding is captured by the strings and binders switch model. Proc Natl Acad Sci USA. 2012;109:16173–8. 22988072 10.1073/pnas.1204799109 PMC3479593

[45] van Steensel B, Belmont AS. Lamina-associated domains: links with chromosome architecture, heterochromatin, and gene repression. Cell. 2017;169:780–91. 28525751 10.1016/j.cell.2017.04.022 PMC5532494

[46] Kim J, Han KY, Khanna N, Ha T, Belmont AS. Nuclear speckle fusion via long-range directional motion regulates speckle morphology after transcriptional inhibition. J Cell Sci. 2019;132:jcs226563. 30858197 10.1242/jcs.226563 PMC6503955

[47] Kim J, Venkata NC, Hernandez Gonzalez GA, Khanna N, Belmont AS. Gene expression amplification by nuclear speckle association. J Cell Biol. 2020;219: e201904046. 31757787 10.1083/jcb.201904046 PMC7039209

[48] Alexander KA, Coté A, Nguyen SC, Zhang L, Gholamalamdari O, Agudelo-Garcia P, et al. p53 mediates target gene association with nuclear speckles for amplified RNA expression. Mol Cell. 2021;81:1666-1681.e6. 33823140 10.1016/j.molcel.2021.03.006 PMC8830378

[49] Chen Y, Zhang Y, Wang Y, Zhang L, Brinkman EK, Adam SA, et al. Mapping 3D genome organization relative to nuclear compartments using TSA-Seq as a cytological ruler. J Cell Biol. 2018;217:4025–48. 30154186 10.1083/jcb.201807108 PMC6219710

[50] Hua N, Tjong H, Shin H, Gong K, Zhou XJ, Alber F. Producing genome structure populations with the dynamic and automated PGS software. Nat Protoc. 2018;13:915–26. 29622804 10.1038/nprot.2018.008 PMC6043163

[51] Tjong H, Li W, Kalhor R, Dai C, Hao S, Gong K, et al. Population-based 3D genome structure analysis reveals driving forces in spatial genome organization. Proc Natl Acad Sci U S A. 2016;113:E1663-1672. 26951677 10.1073/pnas.1512577113 PMC4812752

[52] Girelli G, Custodio J, Kallas T, Agostini F, Wernersson E, Spanjaard B, et al. GPSeq reveals the radial organization of chromatin in the cell nucleus. Nat Biotechnol. 2020;38:1184–93. 32451505 10.1038/s41587-020-0519-y PMC7610410

[53] van Schaik T, Vos M, Peric-Hupkes D, Hn Celie P, van Steensel B. Cell cycle dynamics of lamina-associated DNA. EMBO Rep. 2020;21: e50636. 32893442 10.15252/embr.202050636 PMC7645246

[54] van der Maaten L, Hinton G. Visualizing High-dimensional data using t-SNE. J Mach Learn Res. 2008;9:2579–605.

[55] Rousseeuw PJ. Silhouettes: a graphical aid to the interpretation and validation of cluster analysis. J Comput Appl Math. 1987;20:53–65.

[56] Ramdas A, Garcia N, Cuturi M. On wasserstein two sample testing and related families of nonparametric tests. arXiv; 2015. Available from: http://arxiv.org/abs/1509.02237. Cited 2022 Jul 5.

[57] Eastwood MP, Wolynes PG. Role of explicitly cooperative interactions in protein folding funnels: a simulation study. J Chem Phys. 2001;114:4702.

[58] Zhou J, Ma J, Chen Y, Cheng C, Bao B, Peng J, et al. Robust single-cell Hi-C clustering by convolution- and random-walk-based imputation. Proc Natl Acad Sci U S A. 2019;116:14011–8. 31235599 10.1073/pnas.1901423116 PMC6628819

[59] Welch BL. The generalization of ‘student’s’ problem when several different population varlances are involved. Biometrika. 1947;34:28–35. 20287819 10.1093/biomet/34.1-2.28

[60] Liu S, Zheng P, Wang CY, Jia BB, Zemke NR, Ren B, et al. Cell-type-specific 3D-genome organization and transcription regulation in the brain. bioRxiv. 2023;2023.12.04.570024. 10.1126/sciadv.adv2067 PMC11864200 40009678

[61] Osorio D, Yu X, Yu P, Serpedin E, Cai JJ. Single-cell RNA sequencing of a European and an African lymphoblastoid cell line. Sci Data. 2019;6:112. 31273215 10.1038/s41597-019-0116-4 PMC6609777

[62] SoRelle ED, Dai J, Bonglack EN, Heckenberg EM, Zhou JY, Giamberardino SN, et al. Single-cell RNA-seq reveals transcriptomic heterogeneity mediated by host-pathogen dynamics in lymphoblastoid cell lines. Elife. 2021;10: e62586. 33501914 10.7554/eLife.62586 PMC7867410

[63] Patta I, Zand M, Lee L, Mishra S, Bortnick A, Lu H, et al. Nuclear morphology is shaped by loop-extrusion programs. Nature. 2024;627:196–203. 38355805 10.1038/s41586-024-07086-9 PMC11052650

[64] Krizhevsky A, Sutskever I, Hinton GE. ImageNet classification with deep convolutional neural networks. Commun ACM. 2017;60:84–90.

[65] Zeiler MD. ADADELTA: an adaptive learning rate method. arXiv; 2012. Available from: http://arxiv.org/abs/1212.5701. Cited 2022 Jul 5.

[66] Kullback S, Leibler RA. On information and sufficiency. Ann Math Statist. 1951;22:79–86.

[67] Kruskal JB. Nonmetric multidimensional scaling: a numerical method. Psychometrika. 1964;29:115–29.

[68] Roweis ST, Saul LK. Nonlinear dimensionality reduction by locally linear embedding. Science. 2000;290:2323–6. 11125150 10.1126/science.290.5500.2323

[69] Tenenbaum JB, de Silva V, Langford JC. A global geometric framework for nonlinear dimensionality reduction. Science. 2000;290:2319–23. 11125149 10.1126/science.290.5500.2319

[70] von Luxburg U. A tutorial on spectral clustering. Stat Comput. 2007;17:395–416.

[71] Pedregosa F, Varoquaux G, Gramfort A, Michel V, Thirion B, Grisel O, et al. Scikit-learn: machine learning in python. J Mach Learn Res. 2011;12:2825–30.

[72] McInnes L, Healy J, Melville J. UMAP: uniform manifold approximation and projection for dimension reduction. arXiv; 2020. Available from: http://arxiv.org/abs/1802.03426. Cited 2022 Jul 5.

[73] Scott DW. Multivariate density estimation: theory, practice, and visualization. 2nd ed. Hoboken: Wiley; 2014.

[74] Enright AJ, Van Dongen S, Ouzounis CA. An efficient algorithm for large-scale detection of protein families. Nucleic Acids Res. 2002;30:1575–84. 11917018 10.1093/nar/30.7.1575 PMC101833

[75] Zhang J, Lee D, Dhiman V, Jiang P, Xu J, McGillivray P, et al. An integrative ENCODE resource for cancer genomics. Nat Commun. 2020;11:3696. 32728046 10.1038/s41467-020-14743-w PMC7391744

[76] Kent WJ, Sugnet CW, Furey TS, Roskin KM, Pringle TH, Zahler AM, et al. The human genome browser at UCSC. Genome Res. 2002;12:996–1006. 12045153 10.1101/gr.229102 PMC186604

[77] Robinson JT, Thorvaldsdóttir H, Winckler W, Guttman M, Lander ES, Getz G, et al. Integrative genomics viewer. Nat Biotechnol. 2011;29:24–6. 21221095 10.1038/nbt.1754 PMC3346182

[78] Lee J, Hong W, Cho M, Sim M, Lee D, Ko Y, et al. Synteny portal: a web-based application portal for synteny block analysis. Nucleic Acids Res. 2016;44:W35-40. 27154270 10.1093/nar/gkw310 PMC4987893

[79] Pettersen EF, Goddard TD, Huang CC, Couch GS, Greenblatt DM, Meng EC, et al. UCSF chimera–a visualization system for exploratory research and analysis. J Comput Chem. 2004;25:1605–12. 15264254 10.1002/jcc.20084

[80] Krietenstein N, Abraham S, Venev SV, Abdennur N, Gibcus J, Hsieh THS, et al. Ultrastructural details of mammalian chromosome architecture. Datasets. 4DN Data Portal; 2017. https://data.4dnucleome.org/experiment-set-replicates/4DNES2R6PUEK/. 10.1016/j.molcel.2020.03.003 PMC7222625 32213324

[81] Krietenstein N, Abraham S, Venev SV, Abdennur N, Gibcus J, Hsieh THS, et al. Ultrastructural details of mammalian chromosome architecture. Datasets. 4DN Data Portal; 2017. https://data.4dnucleome.org/experiment-set-replicates/4DNES2M5JIGV/. 10.1016/j.molcel.2020.03.003 PMC7222625 32213324

[82] Ramani V, Deng X, Qiu R, Gunderson KL, Steemers FJ, Disteche CM, et al. Massively multiplex single-cell Hi-C. Datasets. 4DN Data Portal; 2018. https://data.4dnucleome.org/experiment-set-replicates/4DNESUE2NSGS/. 10.1038/nmeth.4155 PMC5330809 28135255

[83] Su JH, Zheng P, Kinrot S, Bintu B, Zhuang X. Genome-scale imaging of the 3D organization and transcriptional activity of chromatin. Zenodo; 2020. https://zenodo.org/record/3928890. 10.1016/j.cell.2020.07.032 PMC7851072 32822575

[84] Yildirim A, Hua N, Boninsegna L, Polles G, Gong K, Hao S, et al. Evaluating the role of the nuclear microenvironment in gene function by population-based modeling. Zenodo; 2022. https://zenodo.org/record/7352276. 10.1038/s41594-023-01036-1 PMC10442234 37580627

[85] Zhan Y, Yildirim A, Boninsegna L, Alber F. Unveiling the role of chromosome structure morphology on gene function through chromosome conformation analysis. Zenodo; 2024. https://zenodo.org/doi/10.5281/zenodo.7563402. 10.1186/s13059-024-03472-8 PMC11827233 39948644

[86] Boninsegna L, Yildirim A, Polles G, Zhan Y, et al. Integrative genome modeling platform reveals essentiality of rare contact events in 3D genome organizations. Github; 2022. https://github.com/alberlab/igm. 10.1038/s41592-022-01527-x PMC9349046 35817938

[87] Zhan Y, Yildirim A, Boninsegna L, Alber F. Unveiling the role of chromosome structure morphology on gene function through chromosome conformation analysis. Github; 2024. https://github.com/alberlab/Conformation_Analysis. 10.1186/s13059-024-03472-8 PMC11827233 39948644

[88] Zhan Y, Yildirim A, Boninsegna L, Alber F. Unveiling the role of chromosome structure morphology on gene function through chromosome conformation analysis. Zenodo; 2024. https://zenodo.org/doi/10.5281/zenodo.14538117. 10.1186/s13059-024-03472-8 PMC11827233 39948644

