# Supplementary material for: Unveiling the role of chromosome structure morphology on gene function through chromosome conformation analysis
Source: Genome Biol. 2025 Feb 13;26:30. doi: 10.1186/s13059-024-03472-8 (PMC11827233; doi:10.1186/s13059-024-03472-8)
Supplement: Supplementary file 1 — Supplementary Material 1. [file 13059_2024_3472_MOESM1_ESM.pdf]

SUPPLEMENTARY INFORMATION

Unveiling the significance of chromosome structure morphology on gene function through chromosome conformation analysis

Yuxiang Zhan[1,2,3], Asli Yildirim[1,2], Lorenzo Boninsegna[1,2], Frank Alber[1,2,3*]

[1]Department of Microbiology, Immunology, and Molecular Genetics, University of California Los Angeles, 520 Boyer Hall, Los Angeles, CA 90095

[2]Institute of Quantitative and Computational Biosciences, University of California Los Angeles, 520 Boyer Hall, Los Angeles, CA 90095

[3]Department of Quantitative and Computational Biology, University of Southern California, 1050 Childs Way, Los Angeles, CA 90089, USA

*Correspondence should be addressed to F.A. falber@g.ucla.edu

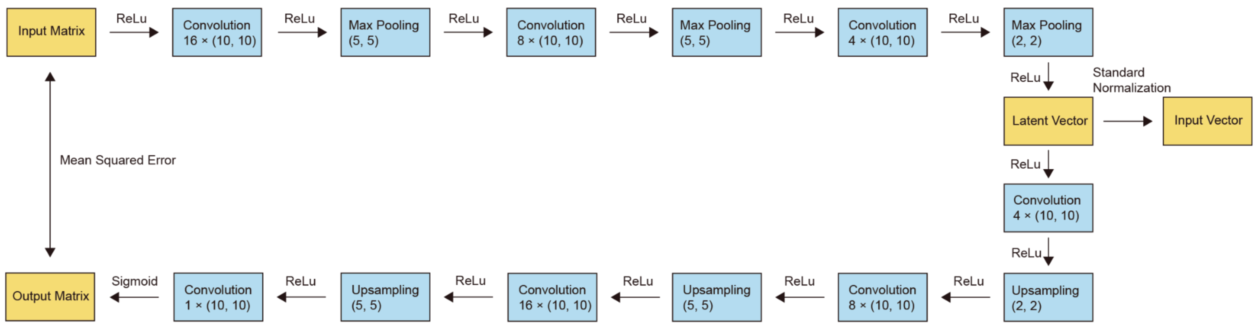

Figure S1: Architecture of the autoencoder The architecture of the autoencoder consists of an encoder and a decoder. The encoder consists of three convolution layers and three max pooling layers. The decoder consists of four convolution layers and three upsampling layers. The loss between the input and the output is measured by the mean squared error.

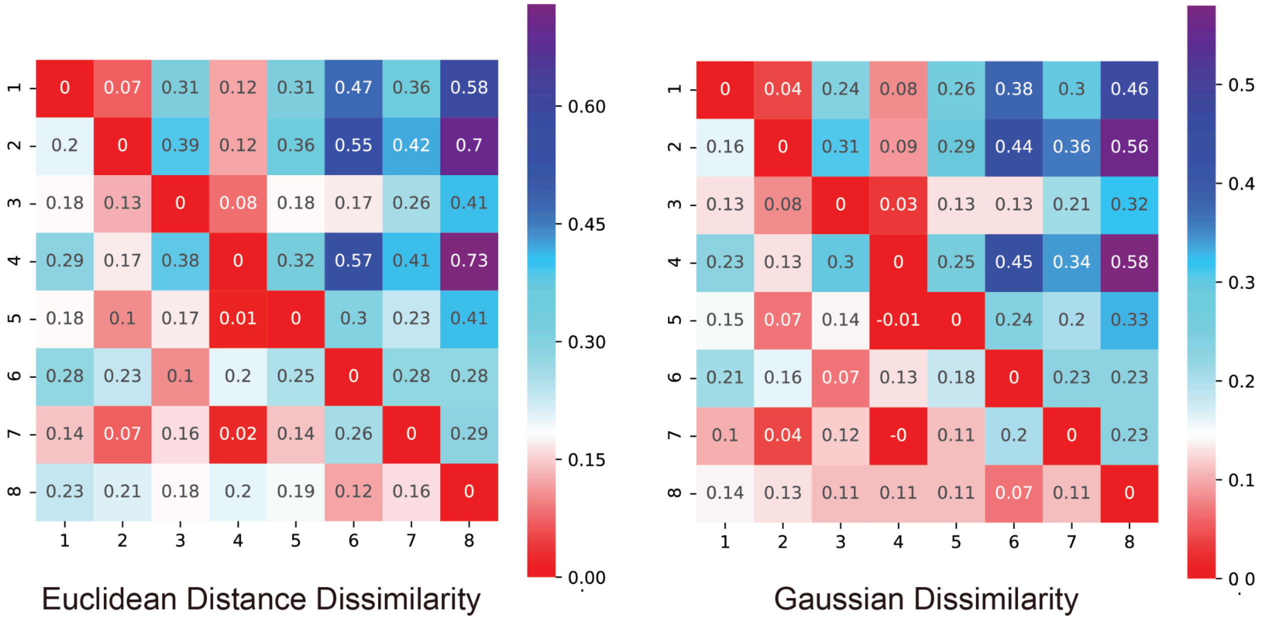

Figure S2: Comparison of different pairwise dissimilarity measurements on GM12878 Chr6 Pairwise dissimilarity between the 8 clusters. The dissimilarity matrices are calculated by measurements of Euclidean distance dissimilarity and Gaussian dissimilarity [1,2]. Each entry represents the log fold ratio between the inter-cluster dissimilarity and the intra-cluster dissimilarity, where positive values indicate the inter-cluster dissimilarity is larger than the intra-cluster dissimilarity.

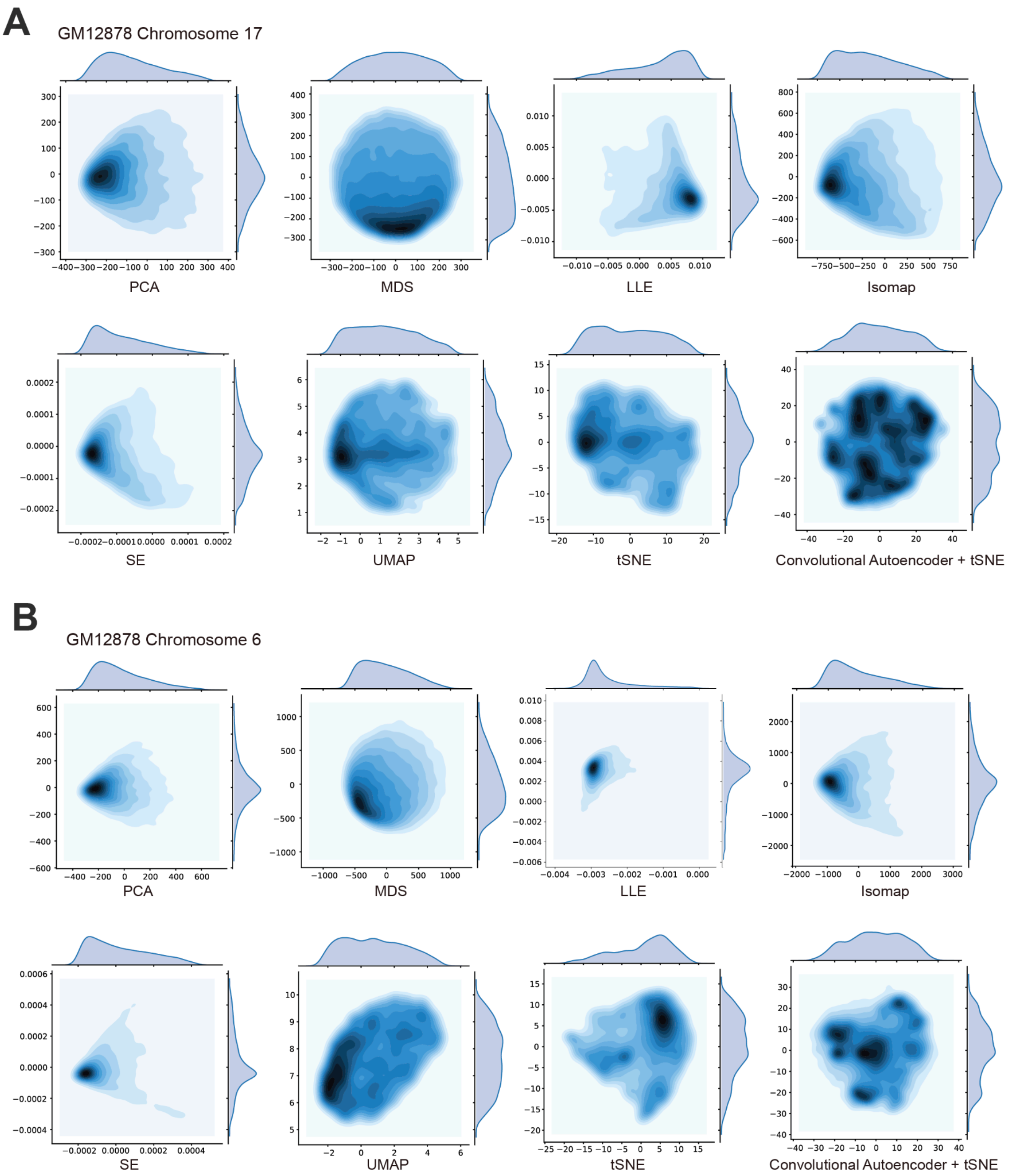

Figure S3: Comparison of different dimension reduction methods on GM12878 Chr17 and Chr6 A, Visualization of different dimension reduction methods: PCA, MDS [3], LLE [4], Isomap [5], SE [6], UMAP [7], tSNE [8]. Each method uses the same input data (the distance vectors derived from the distance matrices of GM12878 chromosome 17). After the embedded data points are obtained, we visualize the distribution by bivariate kernel

density estimation with outlier samples removed. In addition we also plot the two-step dimension reduction (Convolutional Autoencoder + tSNE) proposed in this study. Note that only the two-step dimension reduction is able to generate balanced clusters. PCA, MDS, LLE, Isomap and SE methods are unable to determine distinct clusters (with chromosomes of similar conformational morphology), while UMAP and tSNE (applied directly to distance matrices alone) produced unbalanced clusters, in which the majority of structures were part of only a single cluster. **B,** Similar analysis for GM12878 chromosome 6 with outlier samples removed.

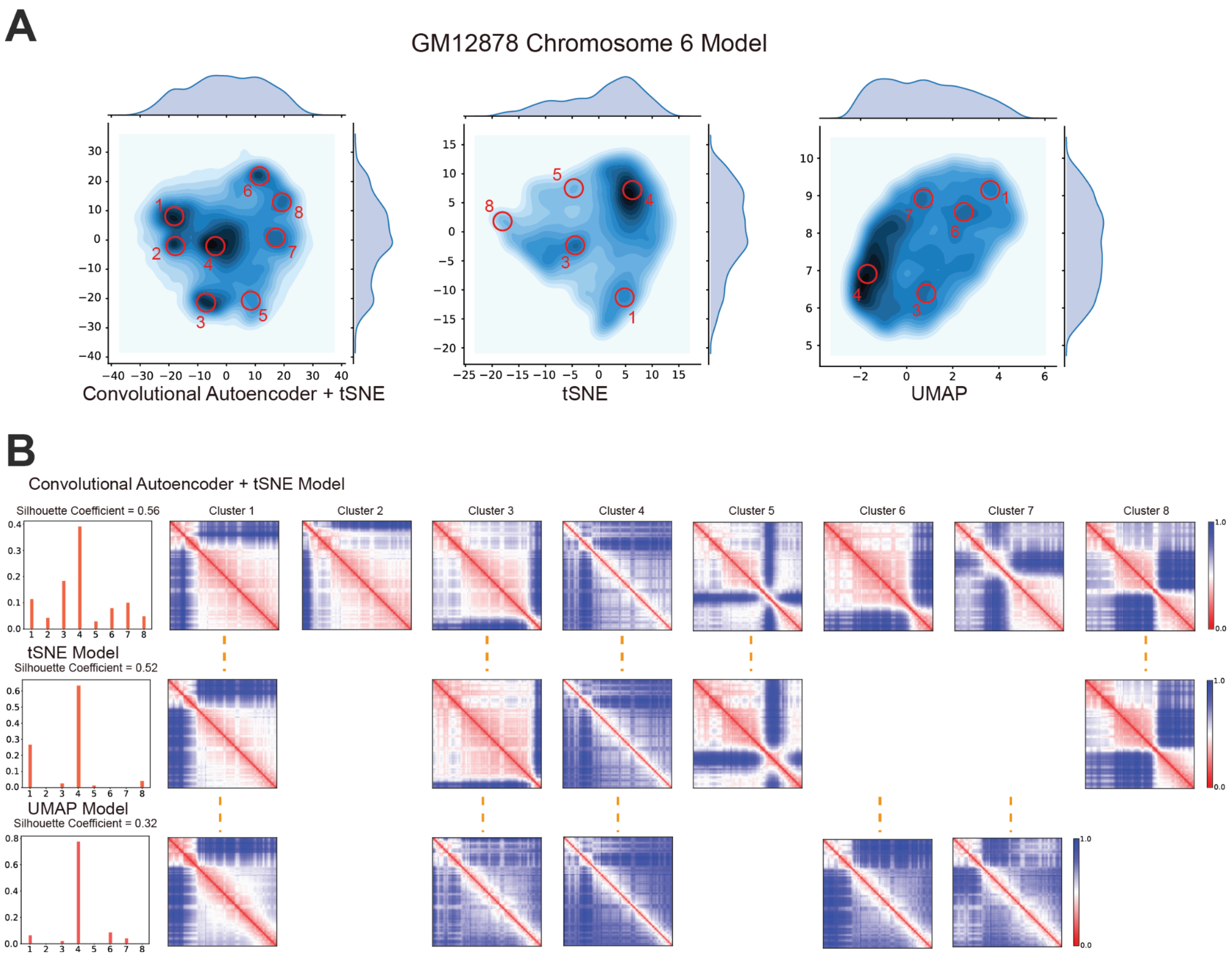

Figure S4: Comparison of different dimension reduction methods on GM12878 Chr6 with clusters A, The 2D density distributions and detected local maxima generated by the two-step dimension reduction, tSNE [8] and UMAP [7] approach on chromosome 6 of the model. **B,** Predicted clusters by the two-step dimension reduction, tSNE and UMAP approach on chromosome 6 of the model. Among these clusters, the ones produced by UMAP are not different in conformation, while the ones predicted by tSNE are almost identical with but fewer than the clusters from the two-step approach.

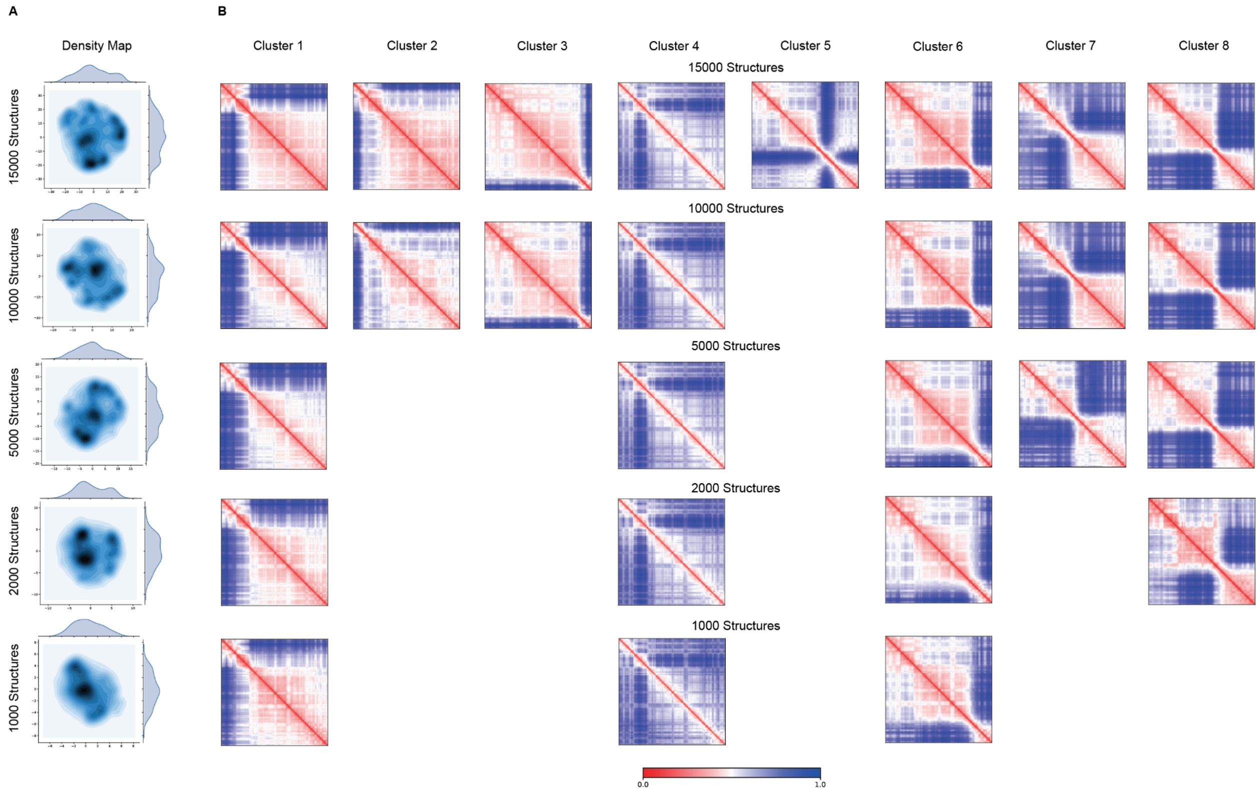

Figure S5: Cluster analysis for results of different numbers of input A, Density distribution of the whole population showed in the conformation space for each number of input. **B,** Distance matrices of the clusters identified for each number of inputs. We observe that fewer clusters are identified when reducing the input size, but clusters are still among those found in the regular results. Clusters are labeled with the same cluster indices from the original analysis.

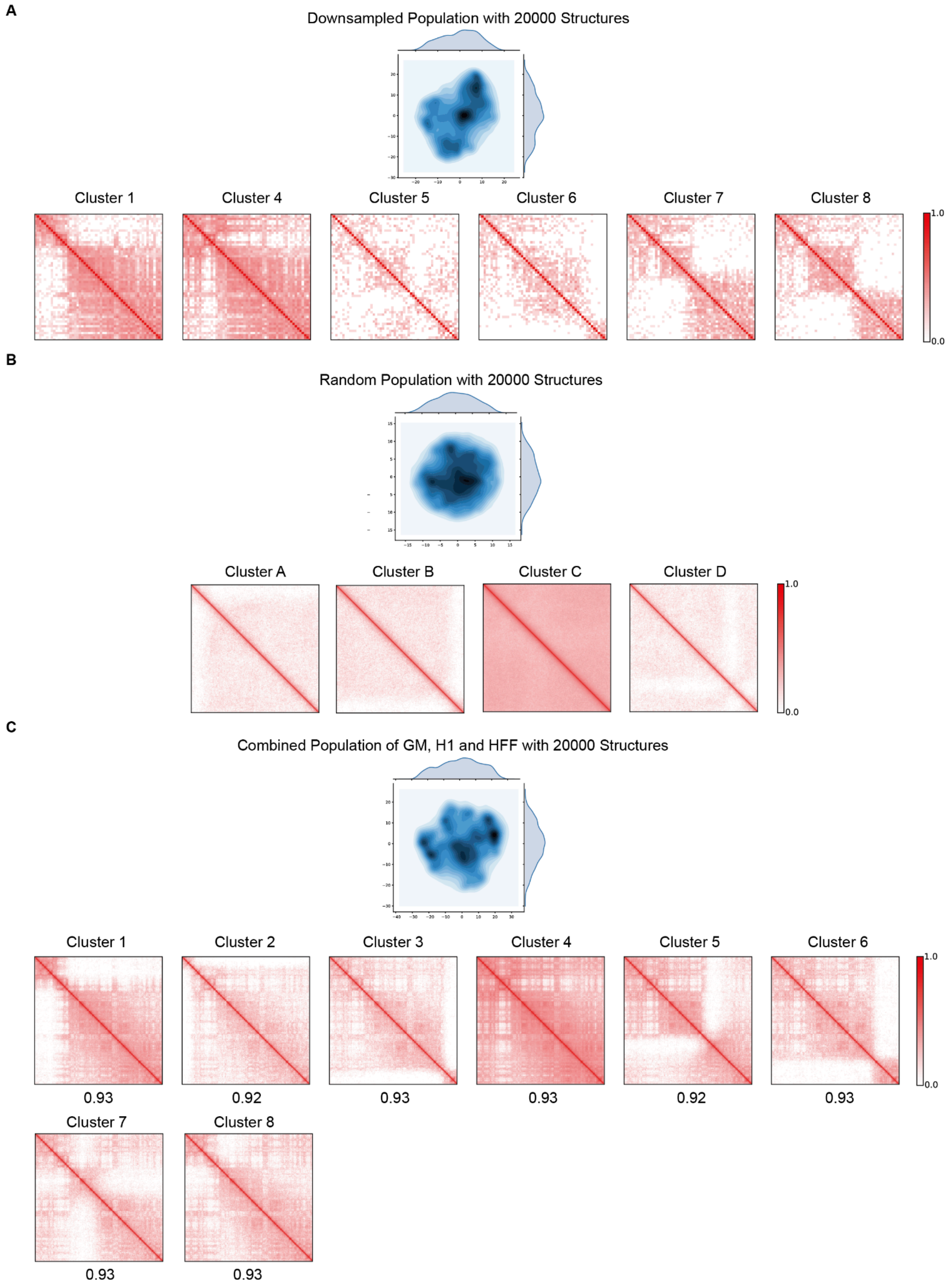

Fig. S6: Cluster analysis for different negative control populations with 20,000 inputs A, (Top row) Density distribution of the downsampled population of structures at 3Mb. Clusters are labeled with the same cluster indices

from the original analysis. (Second row) Contact matrices of clusters identified from the downsampled population at 3Mb. **B,** (Top row) Density distribution of the random population generated without Hi-C restraints. (Second row) Contact matrices of clusters identified from the random population generated without Hi-C restraints. **C,** (Top row) Density distribution of the combined population of GM, H1 and HFF cell lines. (Second row) Contact matrices of clusters identified from the combined population of GM, H1 and HFF cell lines. Clusters are labeled with the same cluster indices from the original analysis together with their Pearson's correlations.

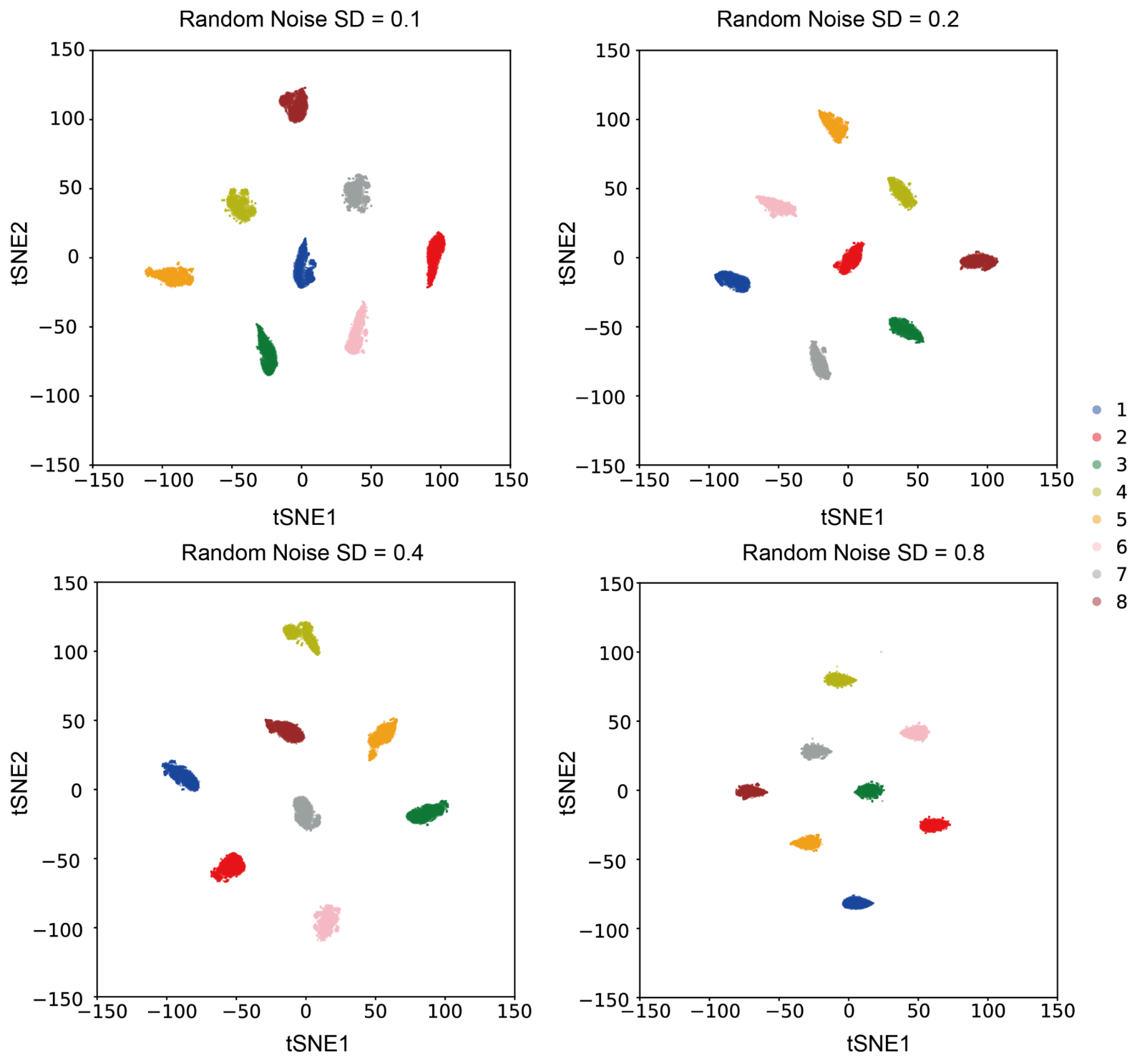

Fig. S7: Dimension reduction analysis for the positive control with 20,000 inputs Different sets of positive control are generated by adding random noise with specific standard deviation from 0.1 to 0.8 to average distance matrices for all clusters as inputs to the two-step dimension reduction approach. We find that our method clearly separates data points generated from different clusters which have clear differences.

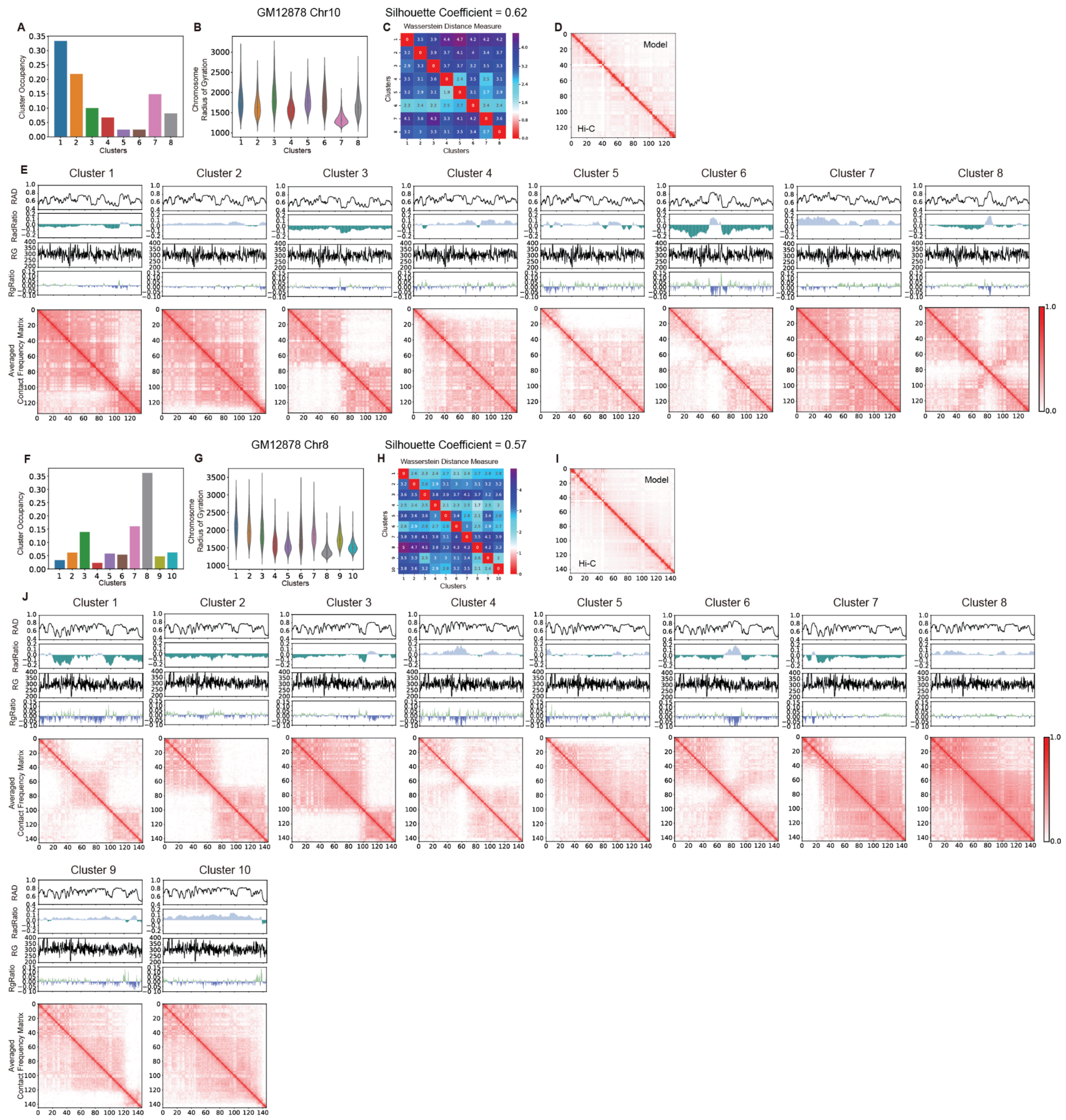
GM12878 Chr10
Silhouette Coefficient = 0.62
Wasserstein Distance Measure
Model
Hi-C
Cluster Occupancy
Chromosome Radius of Gyration
Clusters
Cluster 1
Cluster 2
Cluster 3
Cluster 4
Cluster 5
Cluster 6
Cluster 7
Cluster 8
RAD
RadRatio
RG
RgRatio
Averaged Contact Frequency Matrix
GM12878 Chr8
Silhouette Coefficient = 0.57
Cluster 9
Cluster 10

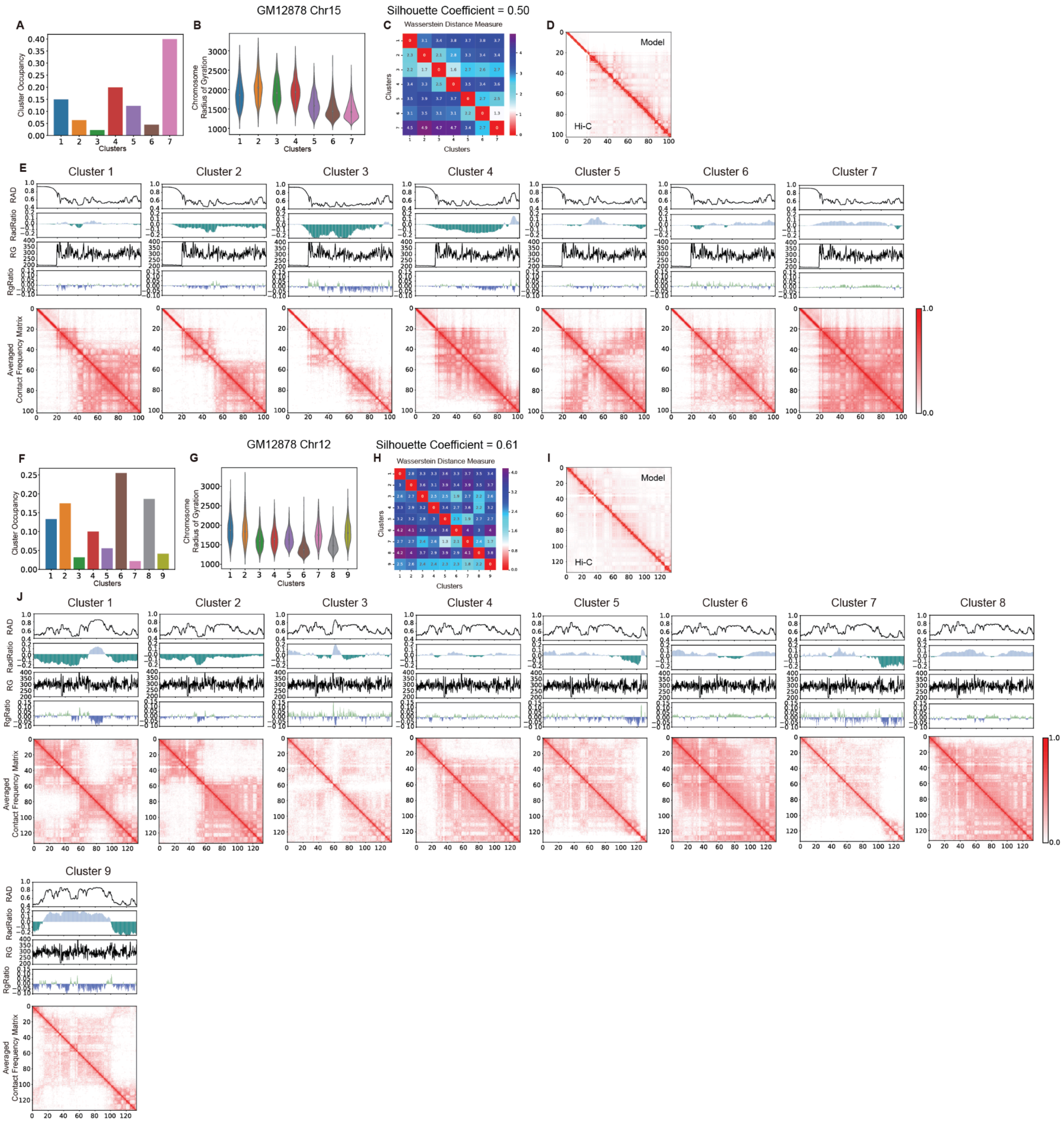
GM12878 Chr15
Silhouette Coefficient = 0.50
A
Cluster Occupancy
Clusters
B
Chromosome Radius of Gyration
Clusters
C
Wasserstein Distance Measure
Clusters
Clusters
D
Model
Hi-C
E
Cluster 1
Cluster 2
Cluster 3
Cluster 4
Cluster 5
Cluster 6
Cluster 7
RAD
RadRatio
RG
RgRatio
Averaged Contact Frequency Matrix
GM12878 Chr12
Silhouette Coefficient = 0.61
F
Cluster Occupancy
Clusters
G
Chromosome Radius of Gyration
Clusters
H
Wasserstein Distance Measure
Clusters
Clusters
I
Model
Hi-C
J
Cluster 1
Cluster 2
Cluster 3
Cluster 4
Cluster 5
Cluster 6
Cluster 7
Cluster 8
Cluster 9
RAD
RadRatio
RG
RgRatio
Averaged Contact Frequency Matrix

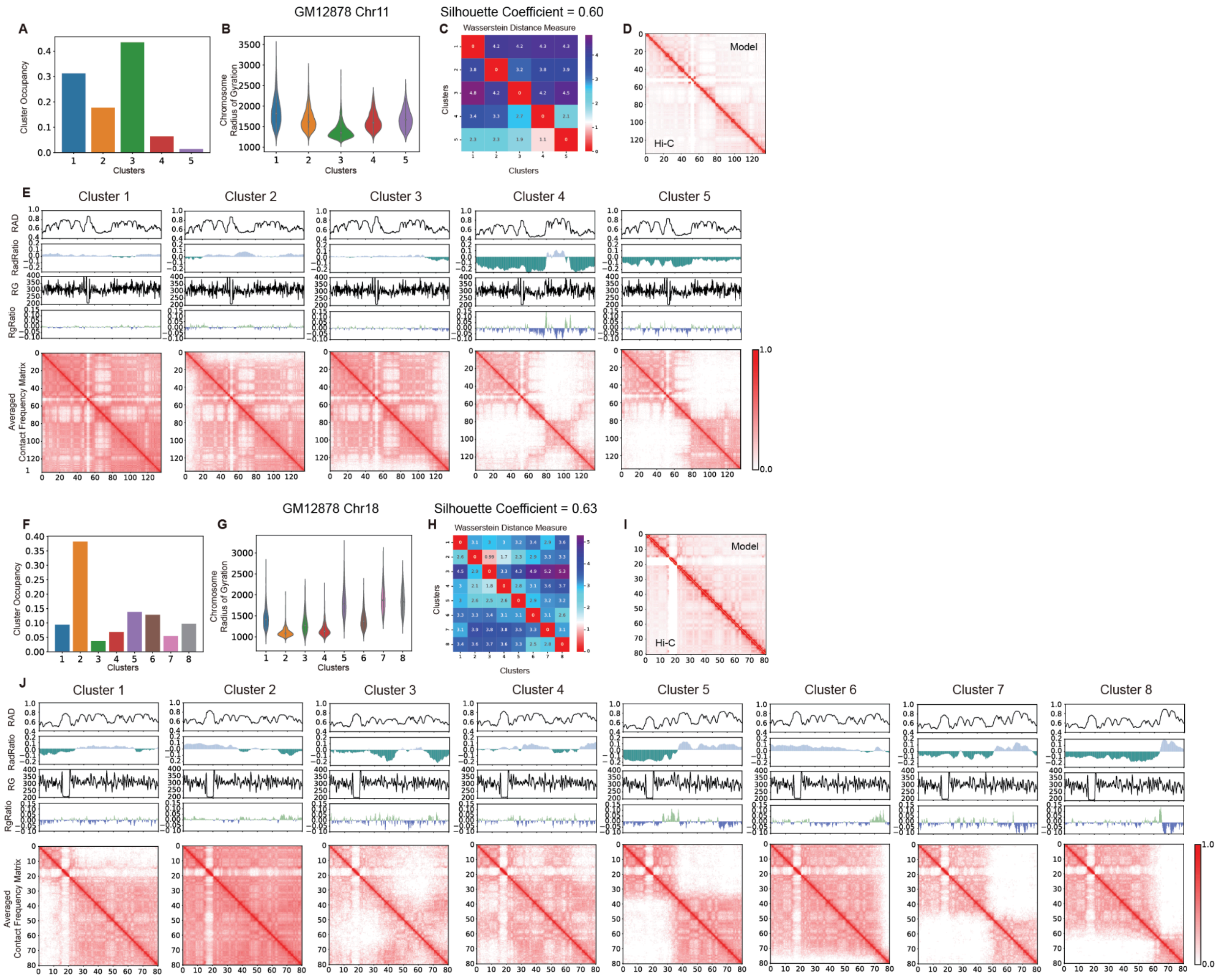
GM12878 Chr11
Silhouette Coefficient = 0.60
A
B
C
D
Cluster Occupancy
Clusters
Chromosome Radius of Gyration
Wasserstein Distance Measure
Model
Hi-C
E
Cluster 1
Cluster 2
Cluster 3
Cluster 4
Cluster 5
RAD
RadRatio
RG
RgRatio
Averaged Contact Frequency Matrix
1.0
0.0
GM12878 Chr18
Silhouette Coefficient = 0.63
F
G
H
I
J
Cluster 6
Cluster 7
Cluster 8

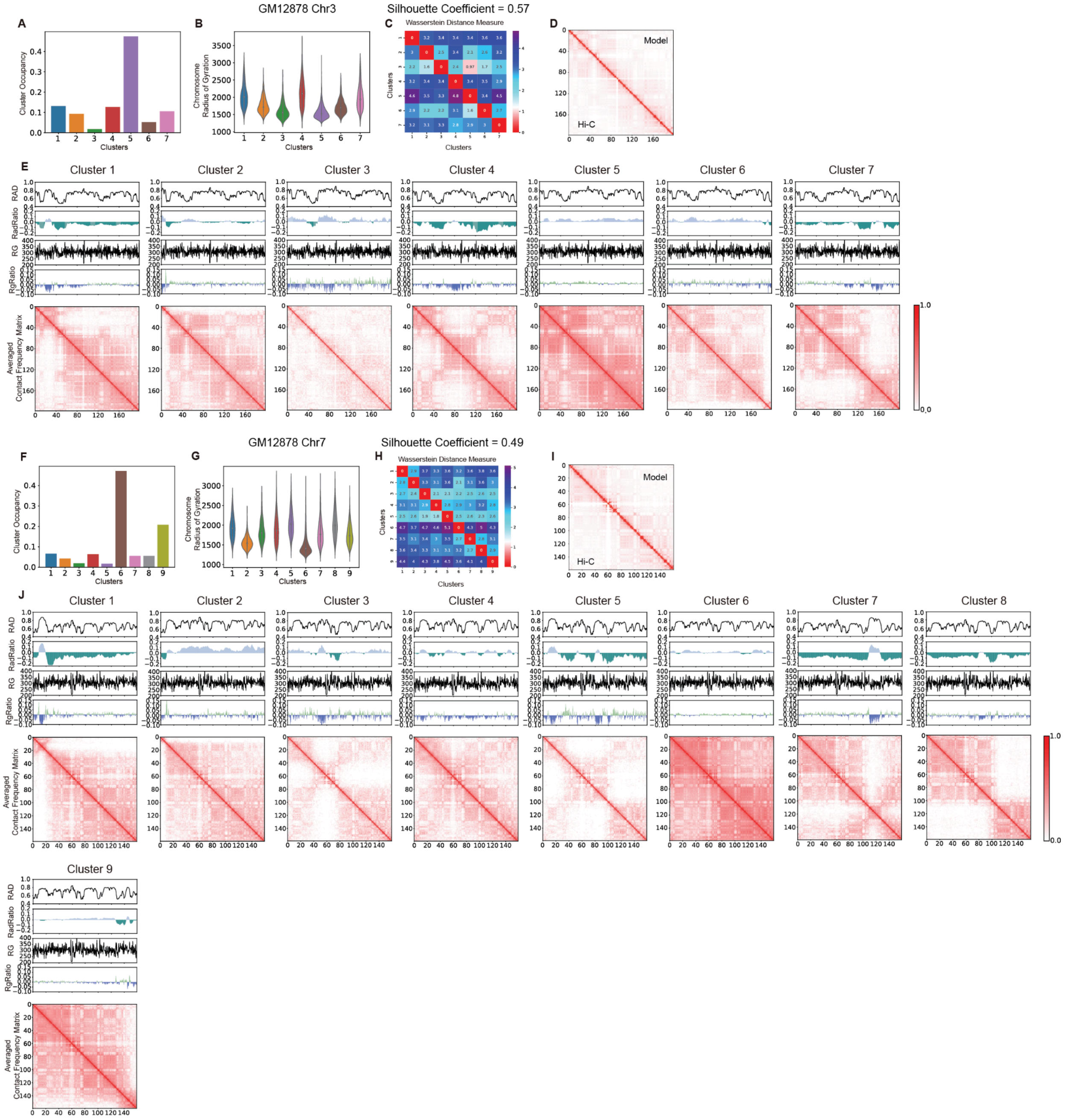
A
B
GM12878 Chr3
Silhouette Coefficient = 0.57
C
Wasserstein Distance Measure
D
Model
Hi-C
Cluster Occupancy
Clusters
Chromosome Radius of Gyration
E
Cluster 1
Cluster 2
Cluster 3
Cluster 4
Cluster 5
Cluster 6
Cluster 7
RAD
RadRatio
RG
RgRatio
Averaged Contact Frequency Matrix
F
G
GM12878 Chr7
Silhouette Coefficient = 0.49
H
Wasserstein Distance Measure
I
Model
Hi-C
J
Cluster 1
Cluster 2
Cluster 3
Cluster 4
Cluster 5
Cluster 6
Cluster 7
Cluster 8
Cluster 9

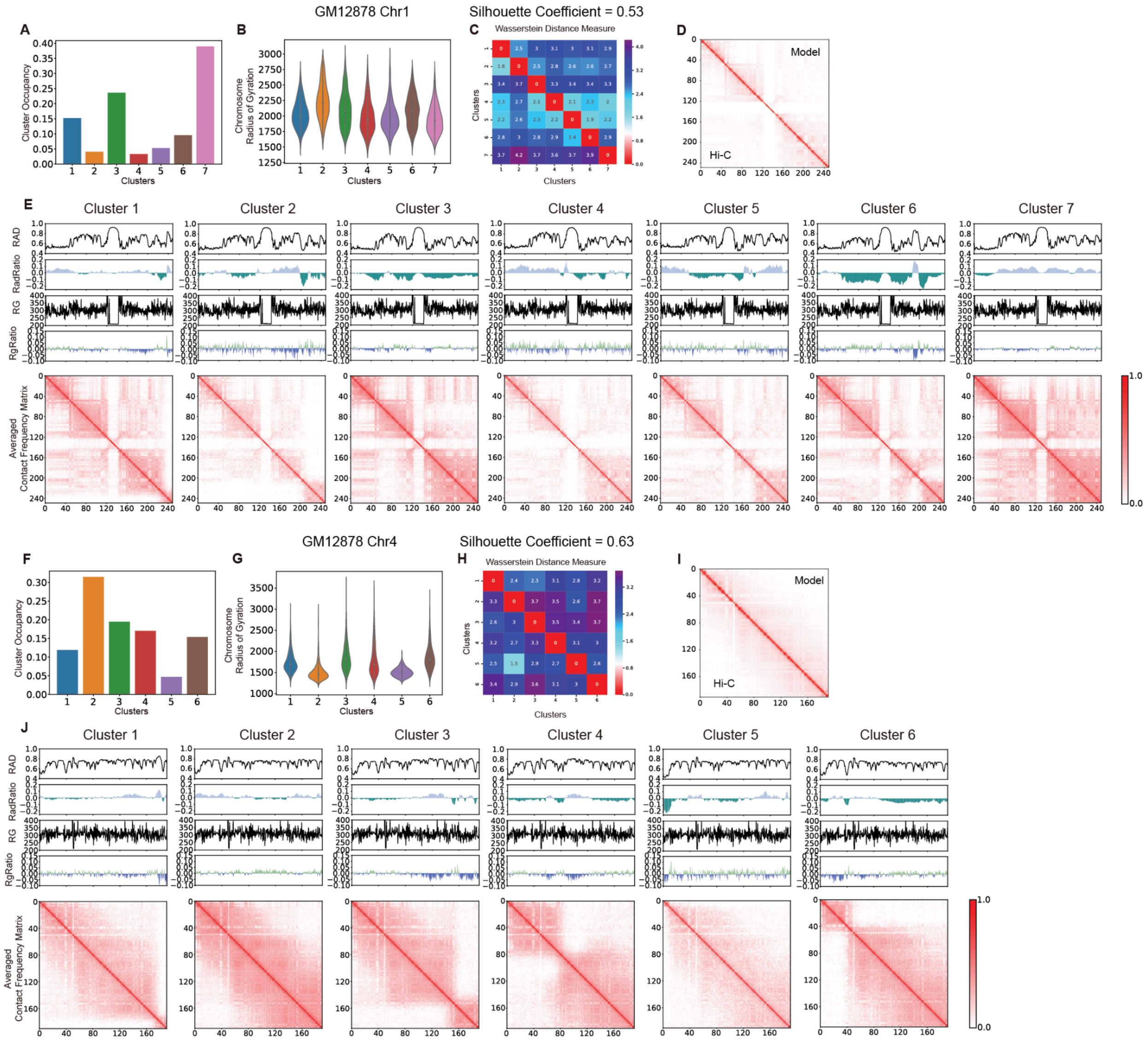

Figure S8: Evaluation of the method's performance on different chromosomes (In each subfigure) A, The cluster occupancy of the predicted clusters of the first chromosome. **B,** The distributions of chromosome radius of gyration for the predicted clusters of the first chromosome. **C,** Pairwise dissimilarity between the clusters of the first chromosome. The dissimilarity matrix is calculated by the measurement of Wasserstein distance [9]. Each entry represents the log fold ratio between the inter-cluster dissimilarity and the intra-cluster dissimilarity, where positive values indicate the inter-cluster dissimilarity is larger than the intra-cluster dissimilarity. **D,** Comparison between the input Hi-C contact frequency matrix and the output reconstructed contact frequency matrix of the first chromosome. **E,** The RAD, RadRatio, RG, RgRatio and contact frequency matrix from each of the clusters of the first chromosome predicted by the two-step dimension reduction method. **F,** The cluster occupancy of the predicted clusters of the second chromosome. **G,** The distributions of chromosome radius of gyration for the predicted clusters of the second chromosome. **H,** Pairwise dissimilarity between the clusters of the second chromosome. The dissimilarity matrix is calculated by the measurement of Wasserstein distance. Each entry represents the log fold ratio between the inter-cluster dissimilarity and the intra-cluster dissimilarity, where positive values indicate the inter-cluster dissimilarity is larger than the intra-cluster dissimilarity. **I,** Comparison between the input Hi-C contact frequency matrix and the output reconstructed contact frequency matrix of the second chromosome. **J,** The RAD, RadRatio, RG, RgRatio and contact frequency matrix from each of the clusters of the second chromosome predicted by the two-step dimension reduction method.

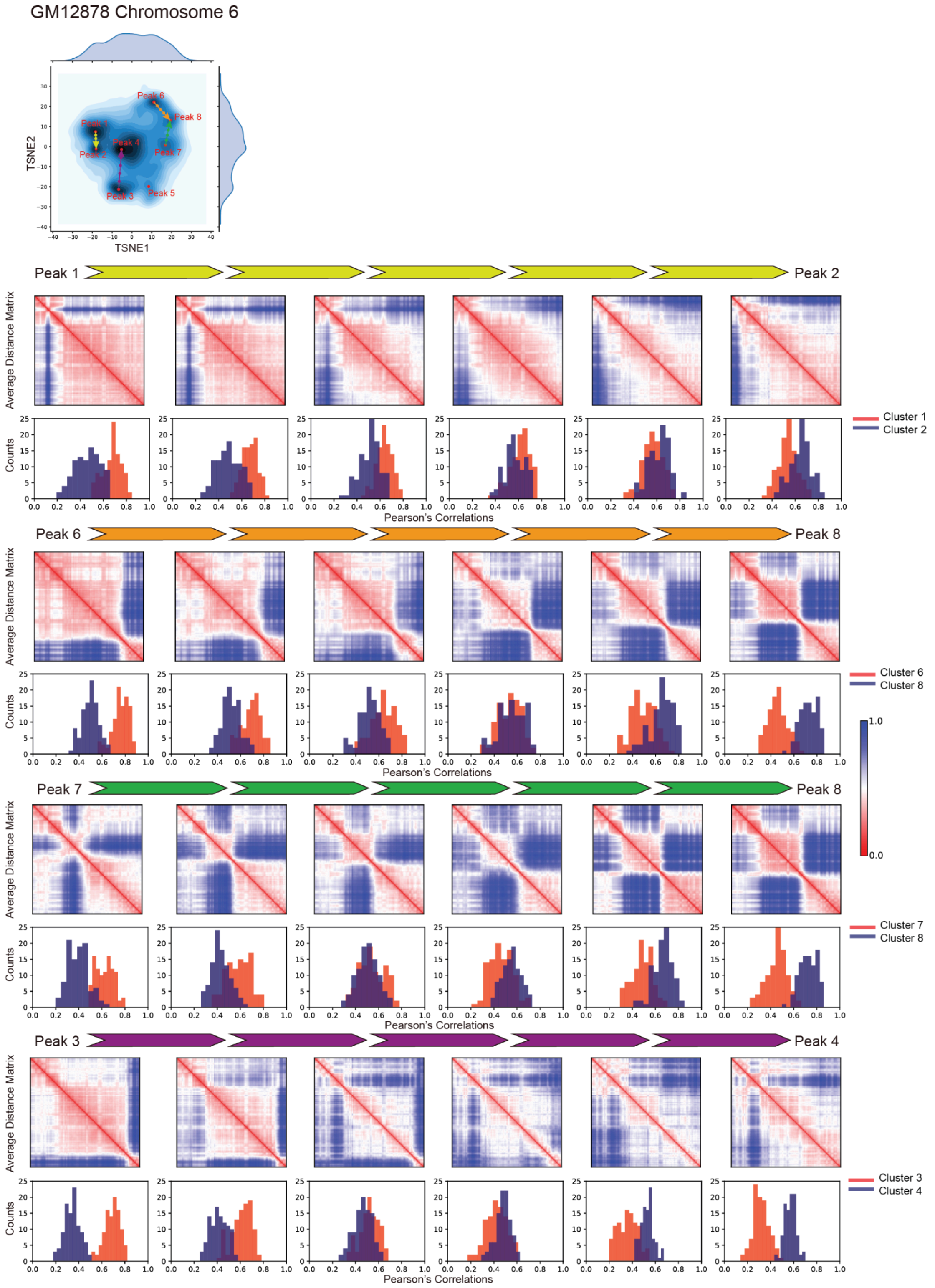

Figure S9: Intermediate conformation analysis on GM12878 Chr6 Various paths in different colors are chosen to select intermediate conformations from one peak (local maximum) to another peak (local maximum). Between every

two peaks (red dots), four locations (dots in certain color) are selected equidistantly along the path (arrow in certain color) for conformation sampling. Each distance matrix is calculated by average of the 100 nearest neighbors of the corresponding location. Also shown below the matrix is the histogram of Pearson's correlations between single-cell distance matrices of the 100 structures and both cluster average distance matrices to which the two peaks belong.

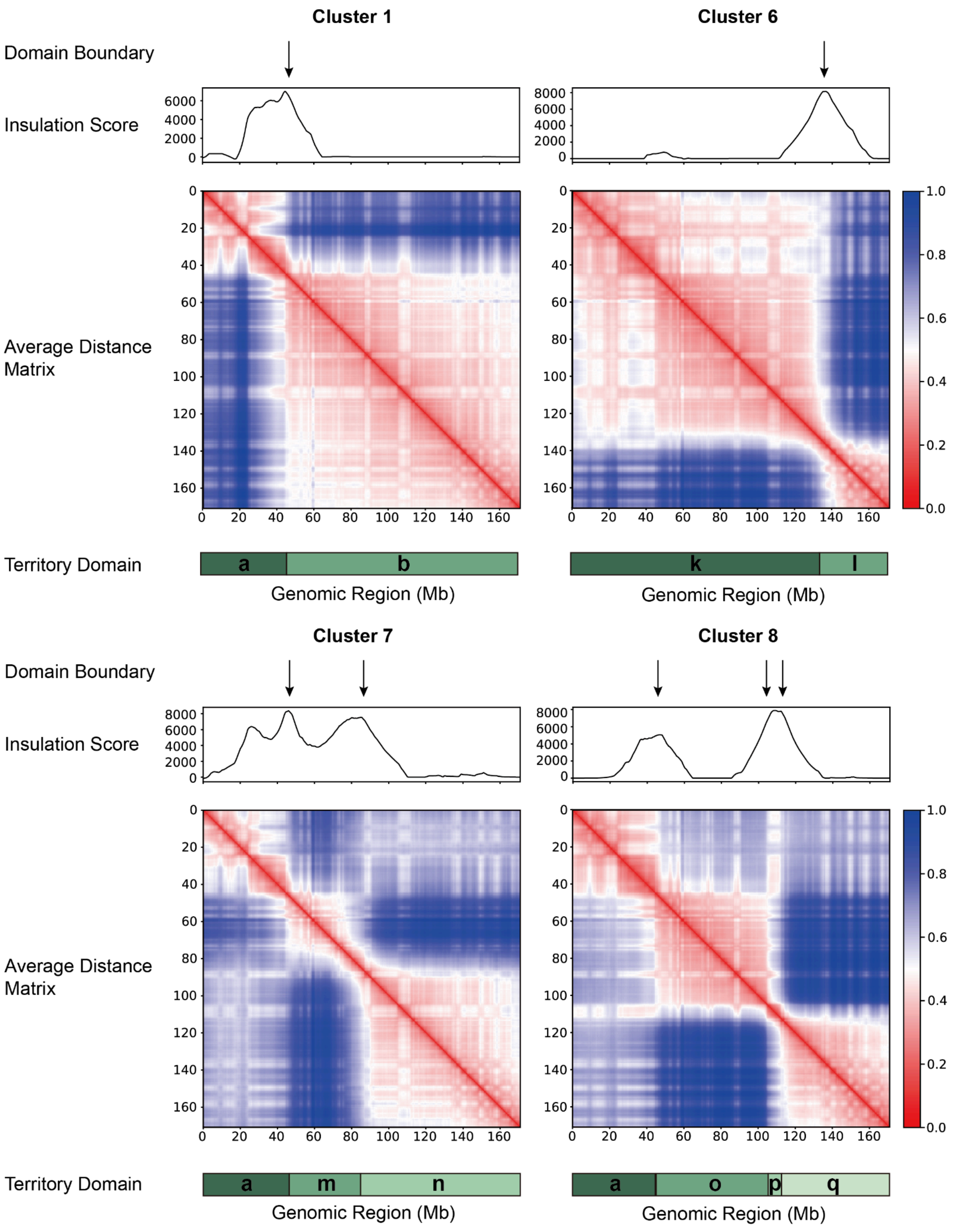

Fig. S10: Domain boundary analysis for selected clusters from GM12878 Chr6 (From top to the bottom) The first row shows the domain boundaries identified. The second row shows the insulation score calculated from the average distance matrix showed in the third row. The third row indicates the territory domains separated by the domain boundaries. We find that the domain boundaries are actually among the major peaks found in the insulation score profile.

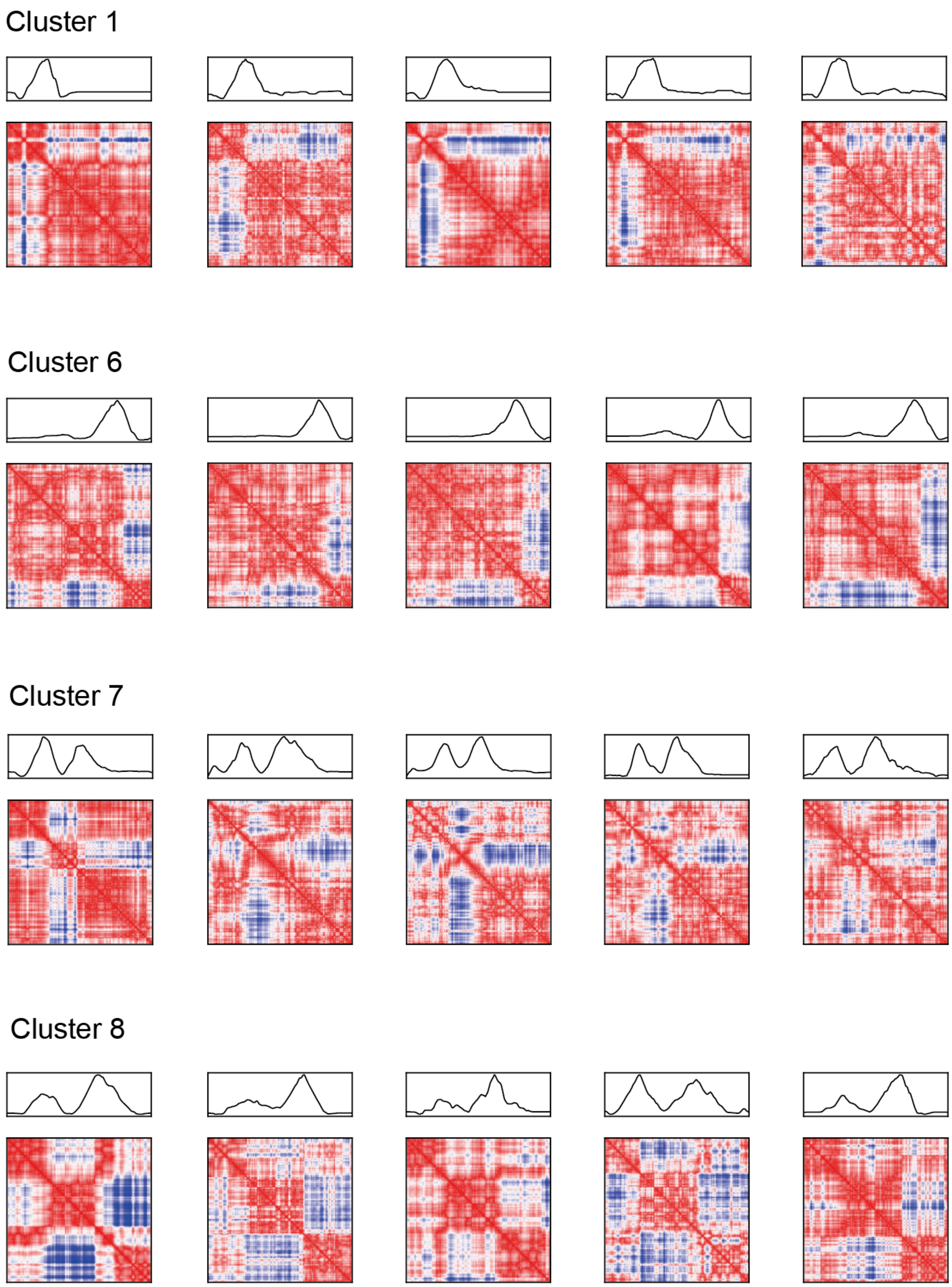

Fig. S11: Selected single-cell examples for each cluster found in GM12878 Chr6 We find that single-cell distance matrices in each cluster show high similarity in large-scale conformation and territory domains which can be detected by the insulation score profile (shown on top of each distance matrix), although variability and flexibly distributed conformations are observed at small scale.

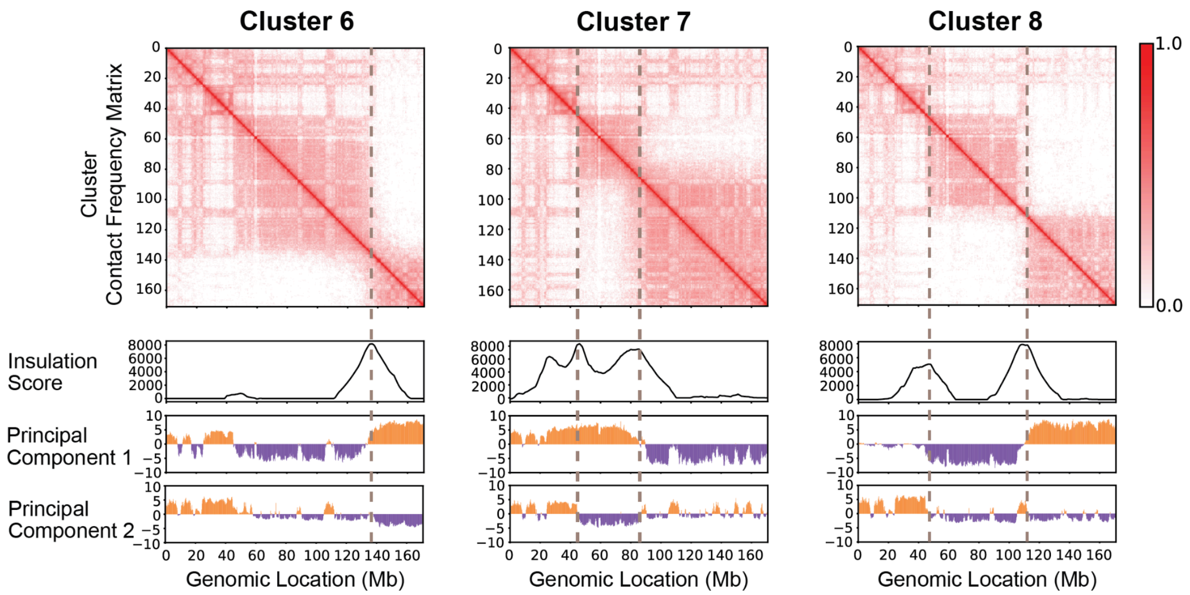

Fig. S12: Comparisons between structural features and compartments on territory domains The principal component 1 (PC1) and the principal component 2 (PC2) showed below cluster contact frequency matrix for cluster 6, cluster 7 and cluster 8 from GM12878 Chr6. We find that PC1 basically correlates with the territory domains, while PC2 are related more with ensemble compartments.

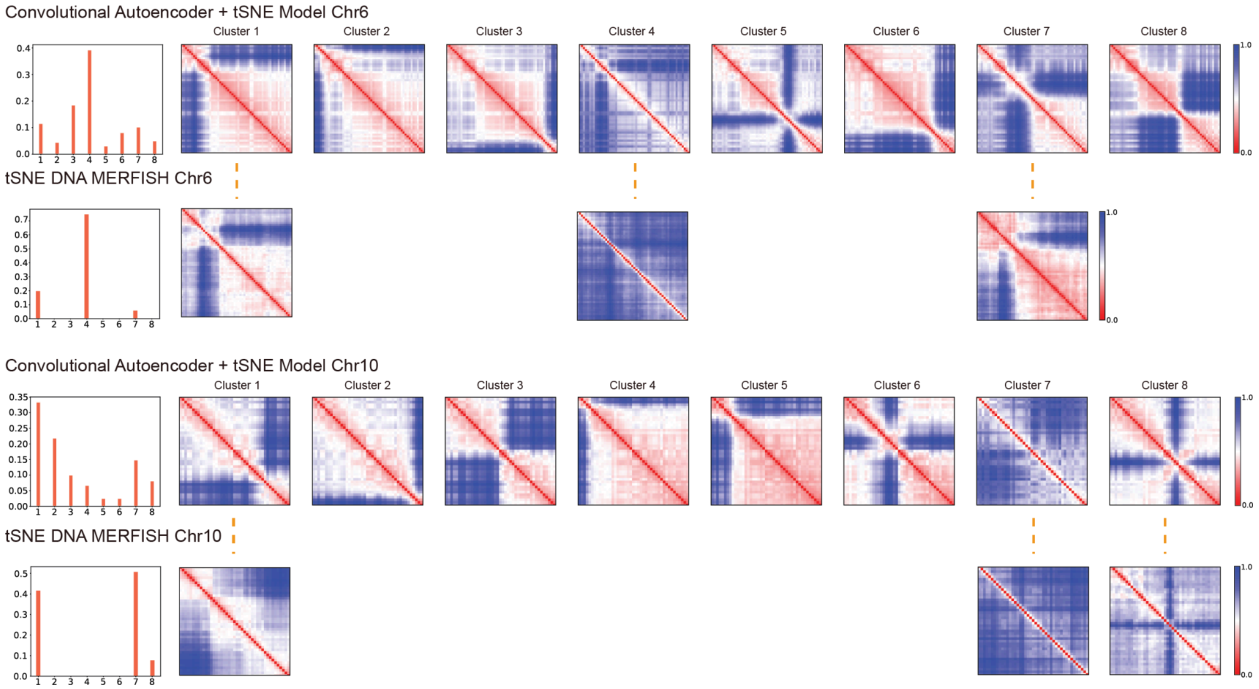

Figure S13: Comparison of clusters generated by model and DNA MERFISH on Chr6 and Chr10 Distance matrices and contact matrices of predicted clusters by tSNE on DNA-MERFISH chromosome 6 and chromosome 10 [10] in comparison with downsampled distance matrices of predicted clusters by the two-step dimension reduction.

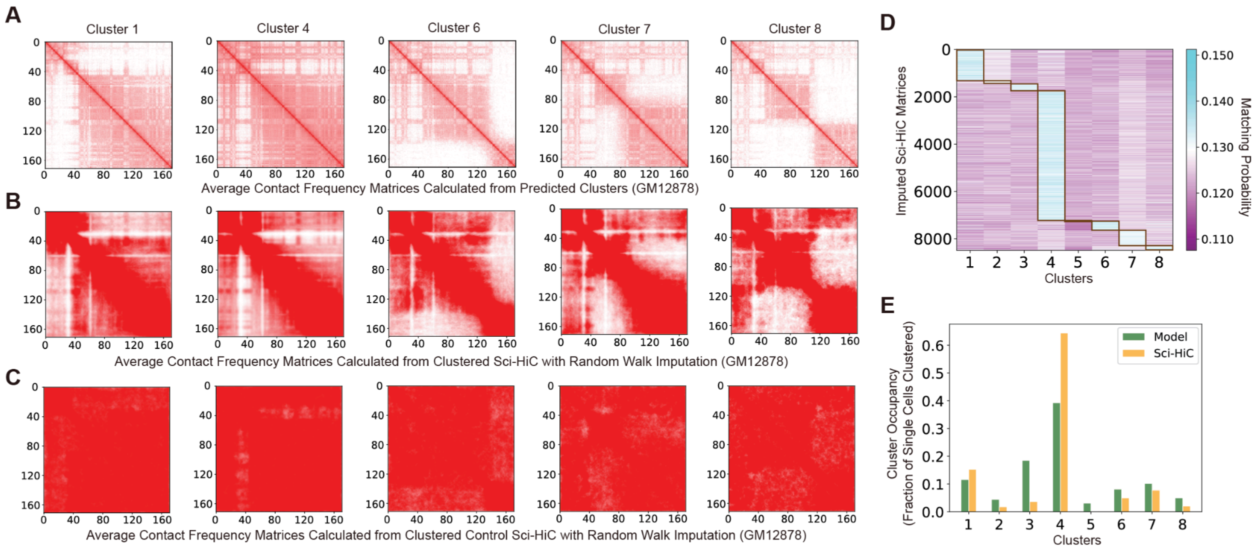

Figure S14: Assessment of predicted clusters by single-cell Hi-C data for GM12878 Chr6 A, The average contact frequency matrices of clusters 1, 4, 6, 7 and 8 calculated for predicted clusters. **B,** Average contact frequency maps calculated from clustered sci-HiC contact maps [11] imputed by convolution and random walk with restart [12]. **C,** Results of the imputed control sci-HiC assessment for different clusters. The contact frequency matrices are constructed by control sci-HiC contact matrices imputed by convolution and random walk with restart [12]. **D,** Matching probabilities indicating the similarities of all classified sci-HiC contact matrices against all modeled clusters. **E,** Comparison of the cluster occupancy for clusters observed in our models and imputed sci-HiC data.

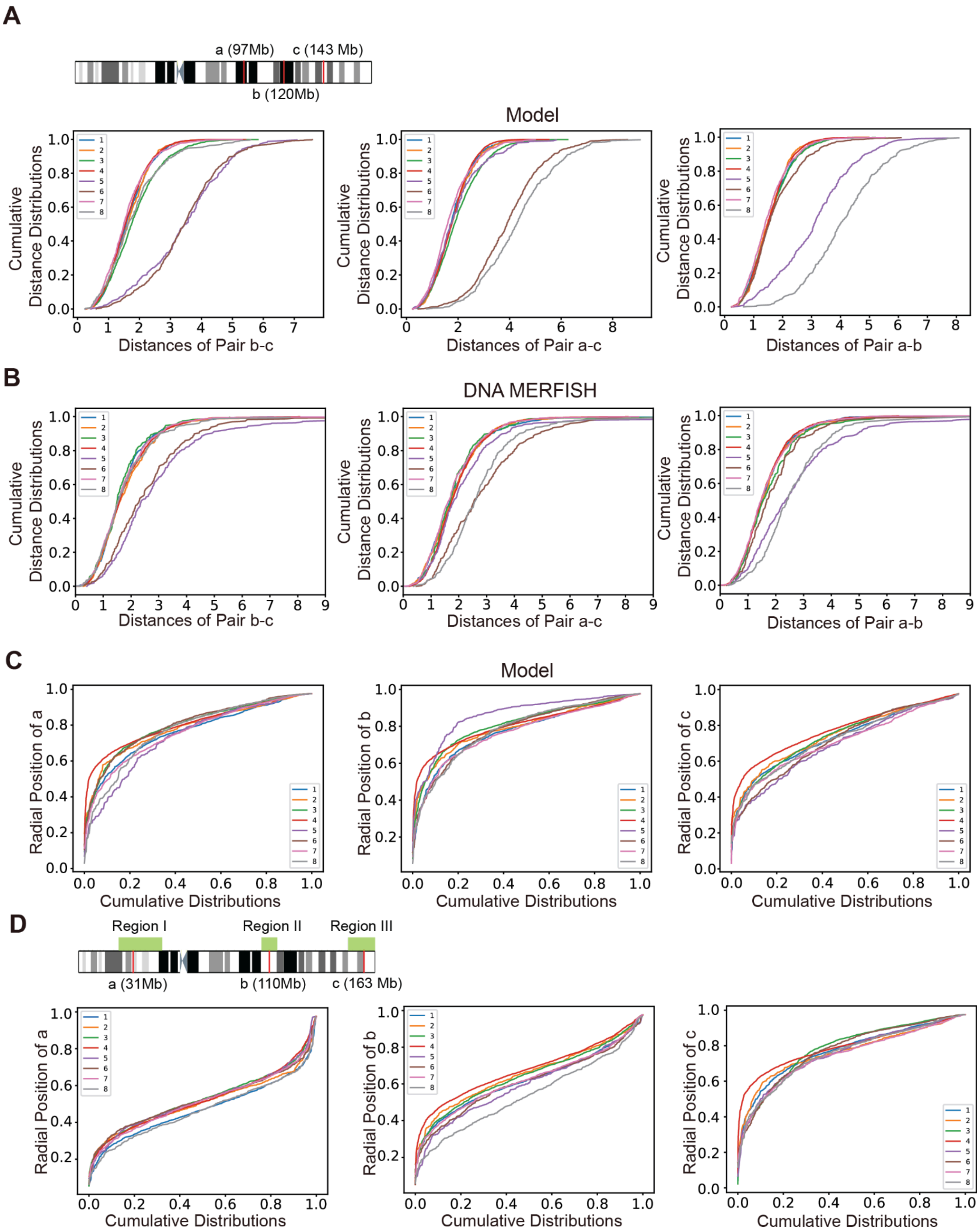
A
a (97Mb)
c (143 Mb)
b (120Mb)
Model
Cumulative Distance Distributions
Distances of Pair b-c
Distances of Pair a-c
Distances of Pair a-b
B
DNA MERFISH
Cumulative Distance Distributions
Distances of Pair b-c
Distances of Pair a-c
Distances of Pair a-b
C
Model
Radial Position of a
Radial Position of b
Radial Position of c
Cumulative Distributions
D
Region I
Region II
Region III
a (31Mb)
b (110Mb)
c (163 Mb)
Radial Position of a
Radial Position of b
Radial Position of c
Cumulative Distributions

Figure S15: Cumulative distance analysis on GM12878 Chr6 A, Cumulative distance distributions of three pairs of selected locations at locus a (97 Mb), locus b (120 Mb) and locus c (143 Mb) for all clusters of modeled structures. **B,** Cumulative distance distributions of three pairs of selected locations at locus a (97 Mb), locus b (120 Mb) and locus c (143 Mb) for all clusters of matched DNA-MERFISH structures [10]. **C,** Cumulative radial position distributions of three selected locations at locus a (97 Mb), locus b (120 Mb) and locus c (143 Mb) for all clusters of modeled structures. **D,** Cumulative radial position distributions of three selected locations at locus a (31 Mb), locus b (110 Mb) and locus c (163 Mb) for all clusters of modeled structures.

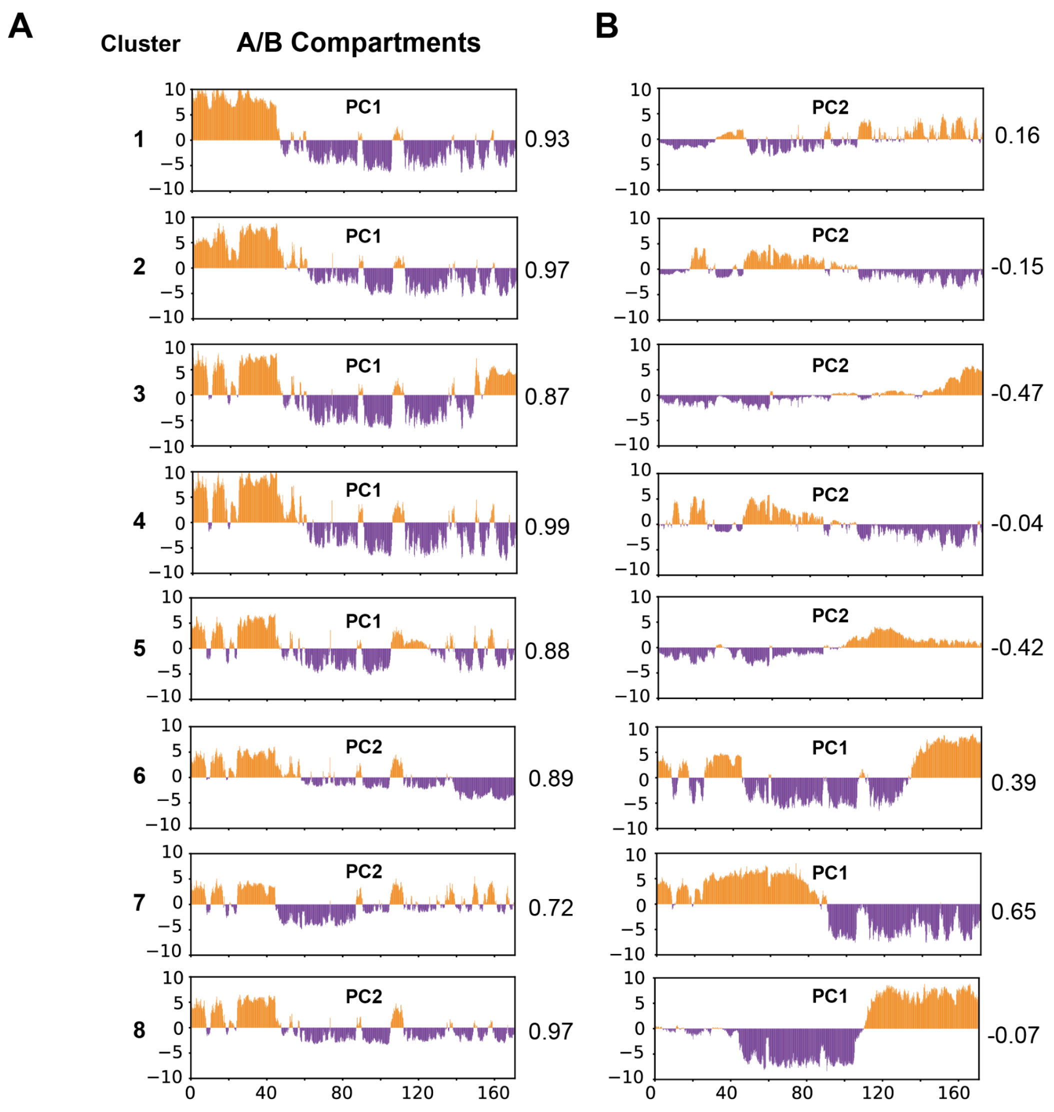

Fig. S16: A/B compartments by principal component analysis on cluster contact frequency matrices of chromosome 6. A, A/B compartment profiles for chromosomes in each cluster is shown together with the Pearson's correlation with the A/B compartment profile calculated from the full ensemble. For clusters 1-5 the compartment profile corresponds to the first eigenvalue (PC1) of the PCA analysis. For clusters 6-8 the compartment profile is represented by the second eigenvalue (PC2). For these clusters the first eigenvalue corresponds to the territory domain segmentation. **B,** The second largest eigenvalue (PC2) profile for clusters 1-5 and the largest eigenvalue (PC1) profiles for clusters 6-8 together with the lower Pearson's correlation of each profile with the ensemble PC1 profile.

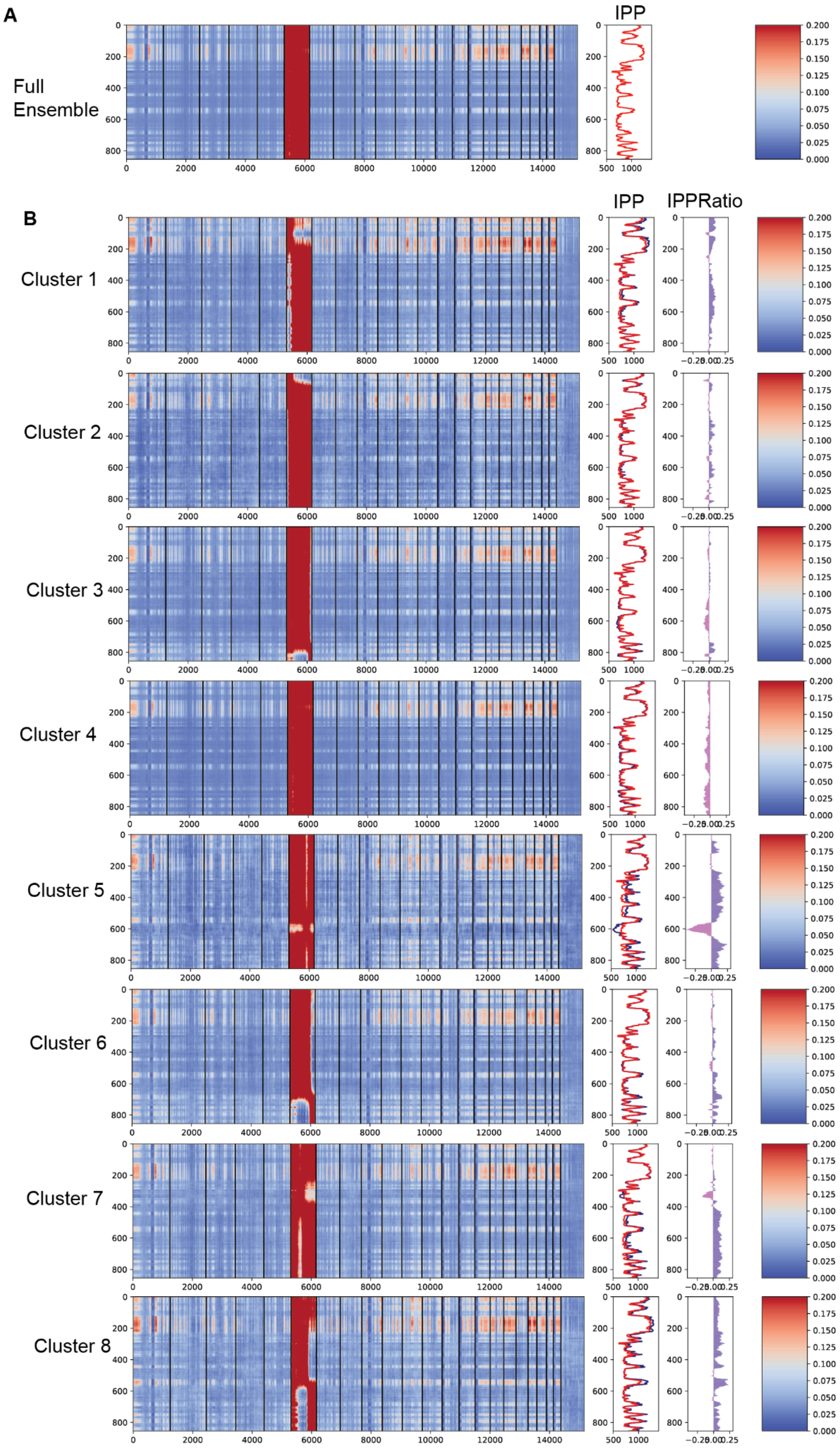
A
Full Ensemble
IPP
B
IPP
IPPRatio
Cluster 1
Cluster 2
Cluster 3
Cluster 4
Cluster 5
Cluster 6
Cluster 7
Cluster 8

Figure S17: Comparison of inter-chromosomal proximity frequency map and associated features for GM12878 Chr6 in different clusters A, (left panel) The average proximity frequency matrix between structures of chromosome 6 and structure of all other chromosomes in the genome for ensemble (Methods), (right panel) Inter-chromosomal proximity profile (IPP), defined as the total number of inter-chromosomal contact proximities of a genomic region with any other chromosomal region of any chromosome divided by the total number of genome structures in a cluster (Methods). The red line shows the genome-wide IPP profile calculated from the whole ensemble of structures, while the blue line shows the IPP profiles calculated from the structures in each cluster. **B,** (left panels) The average proximity frequency matrix between structures of chromosome 6 and structure of all other chromosomes in the genome for different clusters (Methods), (middle panels) Inter-chromosomal proximity profile (IPP). The red line shows the genome-wide IPP profile calculated from the whole ensemble of structures, while the blue line shows the IPP profiles calculated from the structures in each cluster. (right panels) IppRatio, defined as the log ratio of IPP values in a cluster over the IPP value calculated from the ensemble of all clustered structures. Each row of panels shows these properties calculated from different clusters.

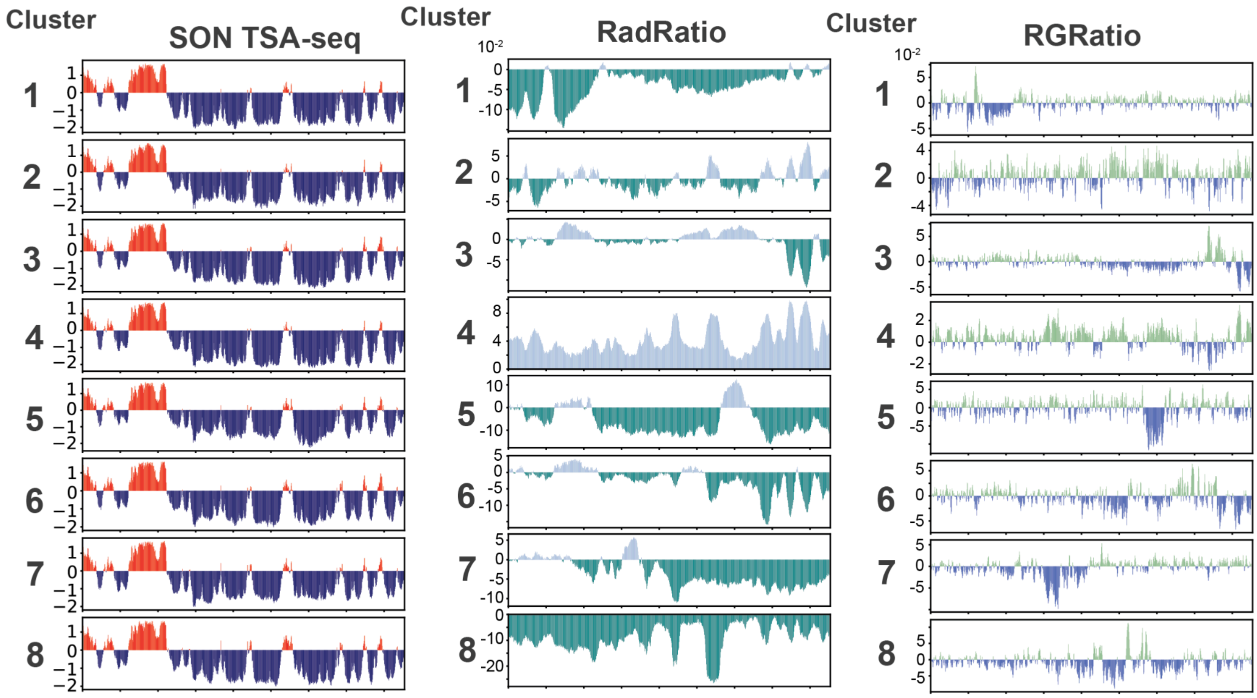

Figure S18: Structural features for all predicted clusters on GM12878 Chr6 (Left panel) Predicted SON TSA-seq of 8 predicted clusters of Chr6. (Middle panel) RadRatio of 8 predicted clusters of Chr6. (Right panel) RgRatio of 8 predicted clusters of Chr6.

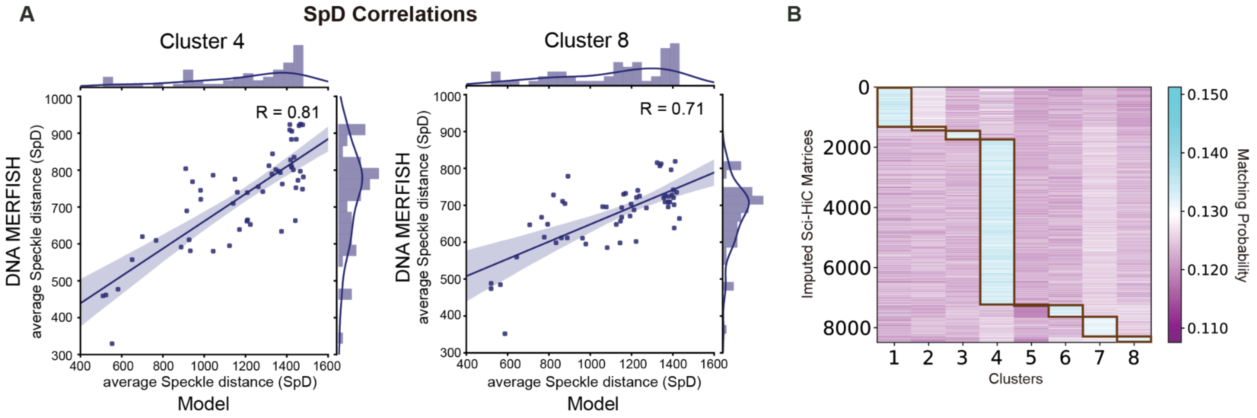

Figure S19: Correlations of speckle distance and speckle association frequency between model and DNA MERFISH on Chr6 A, The scatter plot of speckle distance (SpD) correlation between model and DNA MERFISH [10] with Pearson's correlation coefficient for cluster 4 and cluster 8. **B,** Matching probabilities indicating the similarities between distance matrices of all classified single cell DNA-MERFISH chromosome structures against all modeled clusters. Note that around 60% of the single cell structures are successfully classified and assigned to one of the modeled clusters.

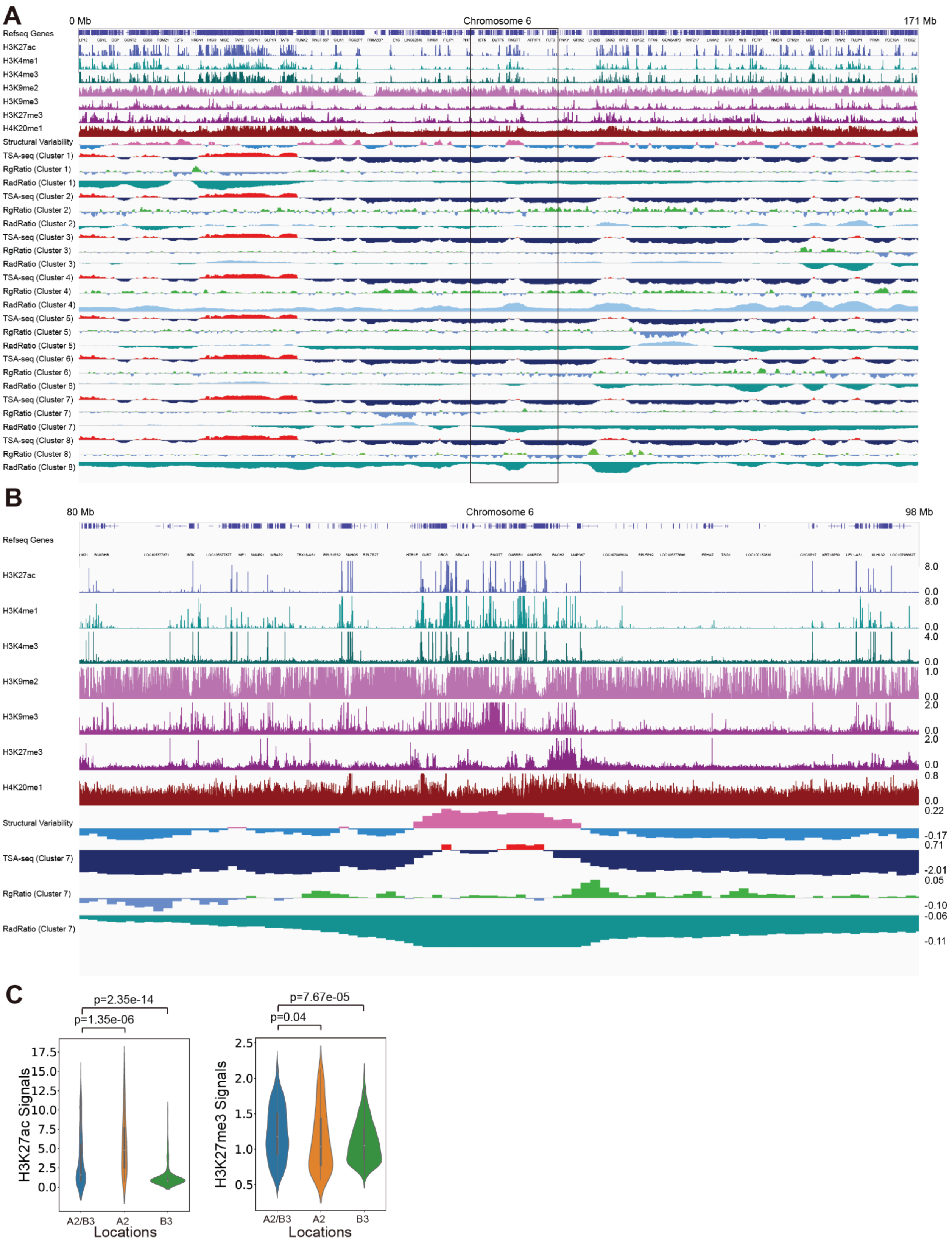
A
0 Mb
Chromosome 6
171 Mb
Refseq Genes
H3K27ac
H3K4me1
H3K4me3
H3K9me2
H3K9me3
H3K27me3
H4K20me1
Structural Variability
TSA-seq (Cluster 1)
RgRatio (Cluster 1)
RadRatio (Cluster 1)
TSA-seq (Cluster 2)
RgRatio (Cluster 2)
RadRatio (Cluster 2)
TSA-seq (Cluster 3)
RgRatio (Cluster 3)
RadRatio (Cluster 3)
TSA-seq (Cluster 4)
RgRatio (Cluster 4)
RadRatio (Cluster 4)
TSA-seq (Cluster 5)
RgRatio (Cluster 5)
RadRatio (Cluster 5)
TSA-seq (Cluster 6)
RgRatio (Cluster 6)
RadRatio (Cluster 6)
TSA-seq (Cluster 7)
RgRatio (Cluster 7)
RadRatio (Cluster 7)
TSA-seq (Cluster 8)
RgRatio (Cluster 8)
RadRatio (Cluster 8)
B
80 Mb
Chromosome 6
98 Mb
Refseq Genes
H3K27ac
8.0
0.0
H3K4me1
8.0
0.0
H3K4me3
4.0
0.0
H3K9me2
1.0
0.0
H3K9me3
2.0
0.0
H3K27me3
2.0
0.0
H4K20me1
0.8
0.0
Structural Variability
0.22
-0.17
TSA-seq (Cluster 7)
0.71
-2.01
RgRatio (Cluster 7)
0.05
-0.10
RadRatio (Cluster 7)
-0.06
-0.11
C
p=2.35e-14
p=1.35e-06
H3K27ac Signals
17.5
15.0
12.5
10.0
7.5
5.0
2.5
0.0
A2/B3
A2
B3
Locations
p=7.67e-05
p=0.04
H3K27me3 Signals
2.5
2.0
1.5
1.0
0.5
A2/B3
A2
B3
Locations

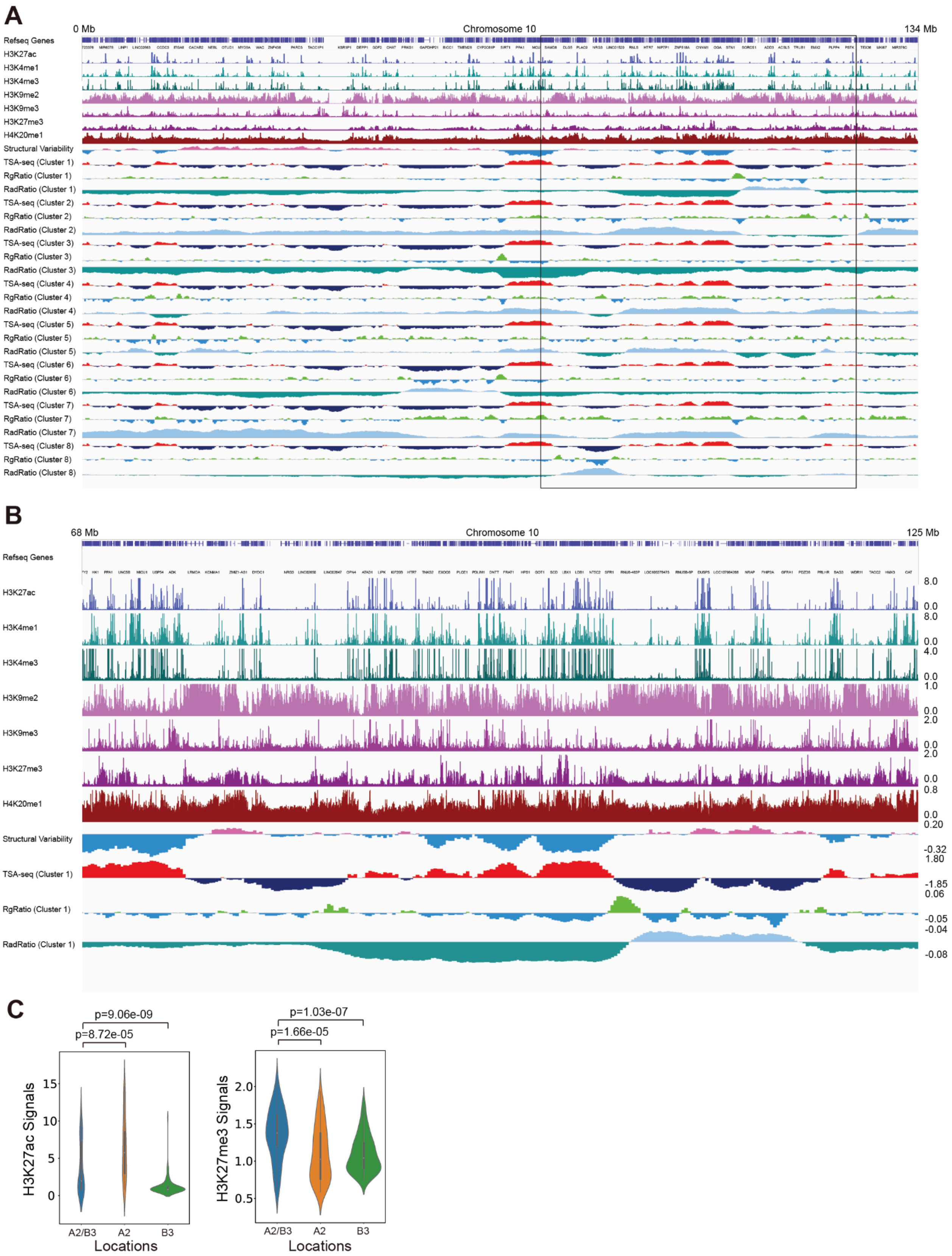
A
0 Mb
Chromosome 10
134 Mb
Refseq Genes
H3K27ac
H3K4me1
H3K4me3
H3K9me2
H3K9me3
H3K27me3
H4K20me1
Structural Variability
TSA-seq (Cluster 1)
RgRatio (Cluster 1)
RadRatio (Cluster 1)
TSA-seq (Cluster 2)
RgRatio (Cluster 2)
RadRatio (Cluster 2)
TSA-seq (Cluster 3)
RgRatio (Cluster 3)
RadRatio (Cluster 3)
TSA-seq (Cluster 4)
RgRatio (Cluster 4)
RadRatio (Cluster 4)
TSA-seq (Cluster 5)
RgRatio (Cluster 5)
RadRatio (Cluster 5)
TSA-seq (Cluster 6)
RgRatio (Cluster 6)
RadRatio (Cluster 6)
TSA-seq (Cluster 7)
RgRatio (Cluster 7)
RadRatio (Cluster 7)
TSA-seq (Cluster 8)
RgRatio (Cluster 8)
RadRatio (Cluster 8)
B
68 Mb
Chromosome 10
125 Mb
Refseq Genes
H3K27ac
H3K4me1
H3K4me3
H3K9me2
H3K9me3
H3K27me3
H4K20me1
Structural Variability
TSA-seq (Cluster 1)
RgRatio (Cluster 1)
RadRatio (Cluster 1)
8.0
0.0
8.0
0.0
4.0
0.0
1.0
0.0
2.0
0.0
2.0
0.0
0.8
0.0
0.20
-0.32
1.80
-1.85
0.06
-0.05
-0.04
-0.08
C
p=9.06e-09
p=8.72e-05
H3K27ac Signals
15
10
5
0
A2/B3
A2
B3
Locations
p=1.03e-07
p=1.66e-05
H3K27me3 Signals
2.0
1.5
1.0
0.5
A2/B3
A2
B3
Locations

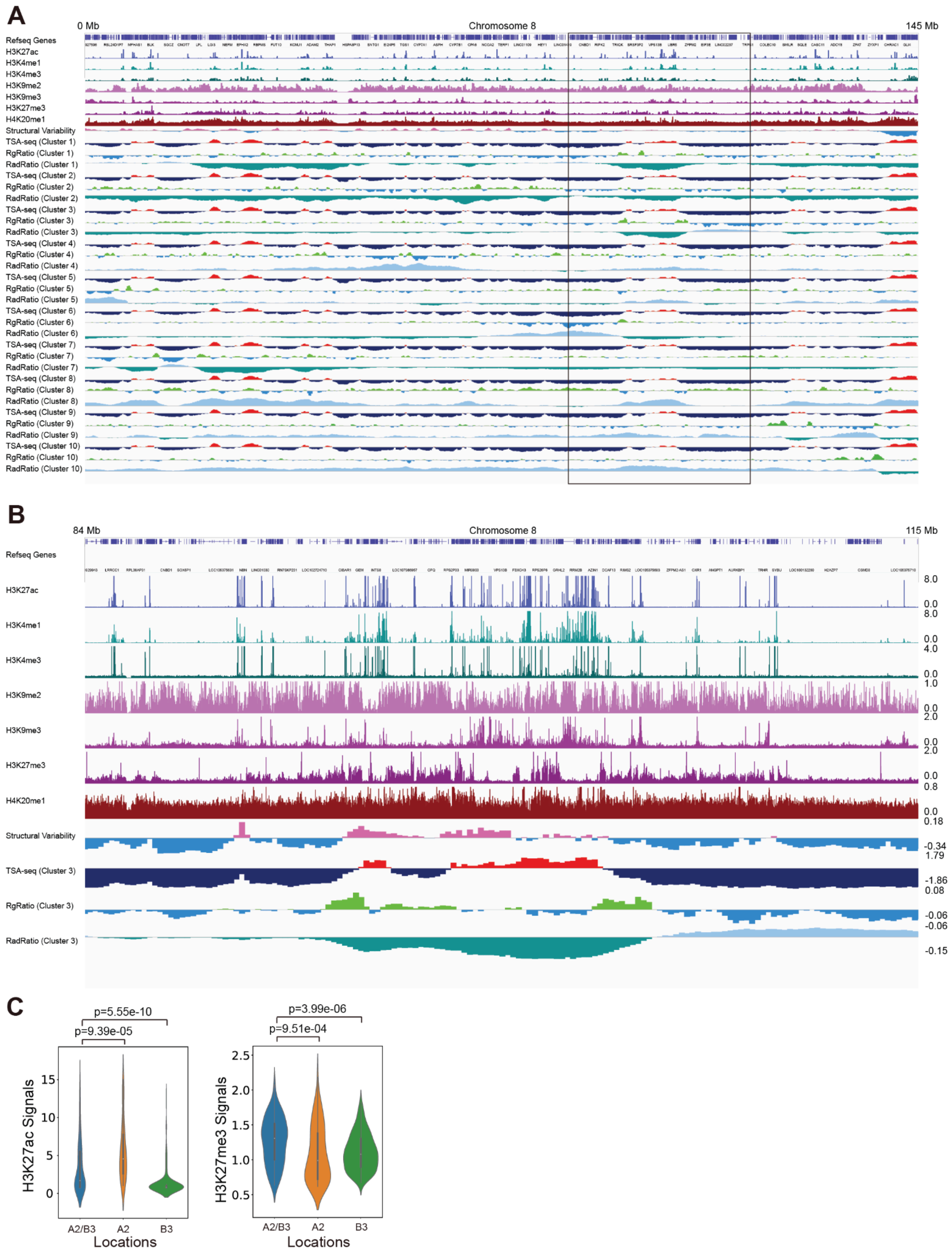
A
0 Mb
Chromosome 8
145 Mb
Refseq Genes
H3K27ac
H3K4me1
H3K4me3
H3K9me2
H3K9me3
H3K27me3
H4K20me1
Structural Variability
TSA-seq (Cluster 1)
RgRatio (Cluster 1)
RadRatio (Cluster 1)
TSA-seq (Cluster 2)
RgRatio (Cluster 2)
RadRatio (Cluster 2)
TSA-seq (Cluster 3)
RgRatio (Cluster 3)
RadRatio (Cluster 3)
TSA-seq (Cluster 4)
RgRatio (Cluster 4)
RadRatio (Cluster 4)
TSA-seq (Cluster 5)
RgRatio (Cluster 5)
RadRatio (Cluster 5)
TSA-seq (Cluster 6)
RgRatio (Cluster 6)
RadRatio (Cluster 6)
TSA-seq (Cluster 7)
RgRatio (Cluster 7)
RadRatio (Cluster 7)
TSA-seq (Cluster 8)
RgRatio (Cluster 8)
RadRatio (Cluster 8)
TSA-seq (Cluster 9)
RgRatio (Cluster 9)
RadRatio (Cluster 9)
TSA-seq (Cluster 10)
RgRatio (Cluster 10)
RadRatio (Cluster 10)
B
84 Mb
Chromosome 8
115 Mb
Refseq Genes
H3K27ac
H3K4me1
H3K4me3
H3K9me2
H3K9me3
H3K27me3
H4K20me1
Structural Variability
TSA-seq (Cluster 3)
RgRatio (Cluster 3)
RadRatio (Cluster 3)
8.0
0.0
8.0
0.0
4.0
0.0
1.0
0.0
2.0
0.0
2.0
0.0
0.8
0.0
0.18
-0.34
1.79
-1.86
0.08
-0.06
-0.06
-0.15
C
p=5.55e-10
p=9.39e-05
H3K27ac Signals
15
10
5
0
A2/B3
A2
B3
Locations
p=3.99e-06
p=9.51e-04
H3K27me3 Signals
2.5
2.0
1.5
1.0
0.5
A2/B3
A2
B3
Locations

Figure S20: Territory domains showed together with different regulation marks on different chromosomes (In each subfigure) A, From the top to the bottom, the displayed features are refseq genes, H3K27ac, H3K4me1, H3K4me3, H3K9me2, H3K9me3, H3K27me3, H4K20me1, the ensemble structural variability, TSA-seq, RgRatio and RadRatio for all clusters. **B,** From the top to the bottom, the displayed features are refseq genes, H3K27ac, H3K4me1, H3K4me3, H3K9me2, H3K9me3, H3K27me3, H4K20me1, the ensemble structural variability, TSA-seq, RgRatio and RadRatio for a selected cluster. **C,** Distributions of H3K27ac and H3K27me3 signals at different locations as well as the p-values of Welch's t-test between distributions. We find significantly higher signals at A2 locations, fewer signals at B3 locations in comparison with A2/B3 boundary locations. For H3K27me3, we observe more signals at A2/B3 boundary locations than A2 and B3 locations.

Chromosomes	Chromosome size (Number of 200kb Regions)	Number of Clusters	Number of Domain Boundaries	Silhouette Coefficient	Number of Clustered Structures
1	1245	7	5	0.53	5251
2	1211	6	5	0.67	4668
3	992	7	4	0.57	7236
4	952	6	5	0.63	4108
5	908	11	9	0.61	7317
6	855	8	8	0.56	7802
7	797	9	6	0.49	9413
8	726	10	6	0.57	5790
9	692	5	4	0.72	3905
10	669	8	6	0.62	7205
11	676	5	3	0.60	7348
12	667	9	6	0.61	4931
13	572	8	6	0.49	9460
14	536	7	5	0.58	5252
15	510	7	4	0.50	7091
16	452	9	4	0.46	6381
17	417	10	5	0.54	8016
18	402	8	5	0.63	4680
19	294	12	5	0.65	5188
20	323	7	3	0.50	8078
21	234	12	3	0.50	7284
22	255	11	6	0.47	9233

Table S1: Chromosome size defined by the number of 200kb windows; number of detected morphology clusters, number of domain boundaries, the corresponding cluster Silhouette coefficient [13] and number of clustered structures for each chromosome of GM12878.

**Clusters**	**P-value**<br><br>**Average Radial Position**
**2**	2.71e-05
**3**	4.06e-12
**4**	8.09e-18
**5**	1.02e-03
**6**	6.17e-09
**7**	9.71e-05
**8**	2.14e-02

Table S2: P-values of the two-sample t-test (Welch's t-test) [14] between cluster 1 and the other clusters of Chr6 for the average radial position of region I (24-48 Mb)

Clusters	P-value<br><br>Average Radial Position	P-value<br><br>Average Speckle Distance
**1**	9.67e-11	4.21e-09
**2**	4.95e-17	4.43e-09
**3**	6.95e-22	3.02e-15
**4**	1.69e-34	7.18e-26
**5**	5.09e-05	5.37e-03
**6**	1.04e-09	2.36e-06
**7**	2.33e-10	1.88e-05

Table S3: P-values of the two-sample t-test (Welch's t-test) [14] between cluster 8 and the other clusters of Chr6 on average radial position and average speckle distance of region II (105-114 Mb)

Clusters	P-value<br><br>Average Radial Position
**1**	1.56e-11
**2**	9.28e-07
**3**	1.10e-03
**4**	1.41e-04
**6**	3.16e-08
**7**	1.39e-13
**8**	3.37e-08

Table S4: P-values of the two-sample t-test (Welch's t-test) [14] between cluster 5 and the other clusters of Chr6 on average radial position of region IV (114-127 Mb)

References

1. Eastwood MP, Wolynes PG. Role of explicitly cooperative interactions in protein folding funnels: A simulation study. J Chem Phys. 2001;114:4702.
2. Cheng RR, Contessoto VG, Lieberman Aiden E, Wolynes PG, Di Pierro M, Onuchic JN. Exploring chromosomal structural heterogeneity across multiple cell lines. Elife. 2020;9:e60312.
3. Kruskal JB. Nonmetric multidimensional scaling: A numerical method. Psychometrika. 1964;29:115–29.
4. Roweis ST, Saul LK. Nonlinear Dimensionality Reduction by Locally Linear Embedding. Science. 2000;290:2323–6.
5. Tenenbaum JB, Silva V de, Langford JC. A Global Geometric Framework for Nonlinear Dimensionality Reduction. Science. 2000;290:2319–23.
6. von Luxburg U. A tutorial on spectral clustering. Stat Comput. 2007;17:395–416.
7. McInnes L, Healy J, Melville J. UMAP: Uniform Manifold Approximation and Projection for Dimension Reduction [Internet]. arXiv; 2020 [cited 2022 Jul 5]. Available from: http://arxiv.org/abs/1802.03426
8. van der Maaten L, Hinton G. Visualizing High-Dimensional Data Using t-SNE. Journal of Machine Learning Research. 2008;9:2579–605.
9. Ramdas A, Garcia N, Cuturi M. On Wasserstein Two Sample Testing and Related Families of Nonparametric Tests [Internet]. arXiv; 2015 [cited 2022 Jul 5]. Available from: http://arxiv.org/abs/1509.02237
10. Su J-H, Zheng P, Kinrot SS, Bintu B, Zhuang X. Genome-Scale Imaging of the 3D Organization and Transcriptional Activity of Chromatin. Cell. 2020;182:1641-1659.e26.
11. Ramani V, Deng X, Qiu R, Gunderson KL, Steemers FJ, Disteche CM, et al. Massively multiplex single-cell Hi-C. Nat Methods. 2017;14:263–6.
12. Zhou J, Ma J, Chen Y, Cheng C, Bao B, Peng J, et al. Robust single-cell Hi-C clustering by convolution- and random-walk-based imputation. Proc Natl Acad Sci U S A. 2019;116:14011–8.
13. Rousseeuw PJ. Silhouettes: A graphical aid to the interpretation and validation of cluster analysis. Journal of Computational and Applied Mathematics. 1987;20:53–65.
14. Welch BL. THE GENERALIZATION OF ‘STUDENT’S’ PROBLEM WHEN SEVERAL DIFFERENT POPULATION VARLANCES ARE INVOLVED. Biometrika. 1947;34:28–35.
