# Supplementary material for: Unveiling the role of chromosome structure morphology on gene function through chromosome conformation analysis
Source: Genome Biol. 2025 Feb 13;26:30. doi: 10.1186/s13059-024-03472-8 (PMC11827233; doi:10.1186/s13059-024-03472-8)
Supplement: Supplementary file 2 — Supplementary Material 2. [file 13059_2024_3472_MOESM2_ESM.docx]

**1^st^ reound**

**Reviewer 1**

The authors explore a method for examining variations in chromosome conformations among cells and investigate the connections between chromosome conformations and gene function. I find this to be a timely and valuable subject, and the content of the manuscript very interesting.

Single-cell genomics and multiplexed imaging have unveiled significant stochastic variabilities in genome structures among individual cells. This stochasticity hampers our ability to establish a causal relationship between genome structures and gene function, presenting a notable challenge in classifying entire chromosome structures. The authors address this challenge by introducing a two-step dimension reduction method. They determine that almost half of all single-cell chromosome structures can be categorized into 5 to 10 prevalent chromosome morphologies, each characterized by distinct interaction patterns and chromosome territory domains. About half of the boundaries are located at syntenic breakpoints. Notably, the authors discover that a majority of these boundaries are conserved across different cell types. They validate their findings using data from RNA- and DNA-MERFISH imaging, along with single-cell sci-HiC data.

I think that the results of the manuscript are sound and valuable. I have only minor comments.

I would suggest a more comprehensive discussion of how this work relates to the outcomes of principled polymer models of chromatin domain formation, such as the loop extrusion and String&Binders Switch models.

While I believe the authors' approach is robust to changes in the parameters of their dimension reduction method, I would recommend addressing this aspect in the manuscript.

**Reviewer 2**

Major concerns:
1. The first question is, how robust is the method? The authors started with 20,000 chromosome structures and ended with 25%-50% of these structures can be clustered into 5-12 subpopulations. What happened when the authors started with variable numbers of chromosome structures (e.g., 10, 000, 15,000, 25,000, or 30, 000, etc)? Also, when run the experiment multiple times, are the results reproducible?
2. Why only 25%-50% of the chromosome structures can be clustered into a few subpopulations? Does it because, in nature, there is no common features/characteristics among the rest of the chromosome structures? The authors said that the main difference between the 5-12 chromosome structure subpopulations lies in their long-range (>10 Mb sequence distance) chromatin interaction patterns, does it mean that all these subpopulations have similar short-range (<= 10 Mb) interaction patterns? Or because the resolution of the method is not sufficient to distinguish the differences in the short-range (<= 10 Mb) interaction patterns?
3. With respect to the interplay between the different levels of the chromosome morphological features, nuclear compartments, and gene transcription, it's hard to conclude as causal relationship without biological validation.
4. The details about territory domain boundaries need to be further established/clarified. What is the size of these boundaries? Do they exist in every chromosome structure? Or can they only be identified in each of the subpopulations? Are their genomic location subpopulation specific? Etc.

Minor concerns:
1. In this sentence (page 3 line 60-62), "and TAD domain boundaries are only rarely observed at the ensemble average position but are rather stochastically distributed, because of dynamic loop extrusion processes [19,34,36,37].", "rarely observed" is not appropriate, because TAD is one of the 3D features that can be regularly observed in bulk Hi-C assays.

**Reviewer 3**

Zhan et al propose a method to cluster single-cell chromosome conformations into 'structural' clusters. Applying this method to structures inferred from population Hi-C map using a data-driven - previously published - approach (IGM), authors show that the clusters are characterized by different contact patterns and domains, and are compatible with single-cell experimental data (MERFISH and sc Hi-C). Then they analyze the characteristics of the clusters and show that they are associated with differential radial positioning, inter-chromosomal contacts, gene expression, etc. Finally, they show some degree of conservations of the domains inferred from different cell types.

Overall, the work is interesting and properly done. In particular, the proposed clustering method seems promising even if its direct application to single-cell experimental data remains to be demonstrated. However, I found the rest of the paper very descriptive and the novelties in terms of new generic concepts on chromosome 3D organization or of specific biological contexts are unclear. In particular, the overall relation between 3D structure, condensation, radial positioning, gene expression at the single cell level was already characterized by the same authors in previous publications (and other authors also) (without the notion of clusters - which is new here - but the main conclusions seem very similar). The possibly interesting discovery of some genes being differentially expressed depending on clusters should be confirmed experimentally and characterized more deeply. The more original parts on syntenic blocks and comparisons between cell types is very sparse. I would suggest the authors - in addition to other concerns (see below) - to clarify the novelties of their results and develop more the last two parts of their work.

Other major:
- On the approach/method:
o Authors convincingly analyzed their approach and its consistency. However, I think it is missing a positive and a negative control where we know a priori the outcome to fully validate it. As a negative control, applications of the method to structures sampled from a simple homopolymer model (simple self-avoiding walk for example) may be used. As a positive control, a mixture of structures with clear differences (eg, presence or absence of loops) may be used.
o Fig.S2 (investigation of the role of the number of input structures): when fewer clusters are inferred with lower numbers of input structures, do these clusters "encompass" the 'new' clusters emerging with high number of inputs?
o How downsampling (or more generally the resolution) is affecting the clustering?
o The majority clusters (eg, cluster 4 for chrom 6) for each chromosome seem always the ones with more compact structures, can the authors comment?
o It is unclear how domains are determined and how boundaries are defined for each cluster in the main text. Can the domain boundaries be inferred directly from the original population Hi-C matrix ?
o Why not applying the method directly to experimental data and check the direct consistency with data-driven structures at the same resolution and with same number of inputs ?
- It misses discussions on the limits of the method and of the approach. For example, fair discussions about the comparisons with experimental (MERFISH, scHIC) are missing. Comparisons with papers by other authors doing similar things would be useful to highlight the originality of the present work.


Minor:
- The term 'nuclear topography' is interesting but unusual in the field. It might be clearly defined when introduced for the first time.
- L95-96 "This is because certain …": unclear, please reformulate.
- The end of the introduction is a very long, very detailed summary of the paper which is redundant with the abstract and the conclusion part. I would suggest to just enumerate what authors will describe in the rest of the paper without detailing the results.
- It would be interesting for visual comparison to show the experimental Hi-C map at 200kbp resolution for the different chromosomes and cell types investigated in the paper.
- It should be stated in the figure caption and in the text that the cell type mostly investigated is GM12878.
- In Table S1 may be nice to have the proportion of the structures that are clustered.
- Fig.2D: in the caption, it is unclear what are the specific genomic regions I, II ,II below the RadRation & RGRatio profiles.
- Fig.2F: it seems that the decay of the P(s) curve is slower than the expected exponent ( ~ -1), why ?
- The term 'chromatin fiber compaction' could be confusing as it may refer to the local compaction of the fiber (nucleosome positioning, density) or to the larger-scale condensation of the fiber (which is the meaning used in the manuscript). It may be better to use another term or to clearly define it.
- L242-244 "Overall, the relative …": unclear, please reformulate.
- Fig.S9: the caption should be more detailed to guide the reader. Maybe I misunderstood, but it seems that the 'null', randomized model is obtained by fully randomizing all the contacts, a better null model would be to randomize contact per sub-diagonal to conserve the overall polymeric structure.
- L298-342, there are many 'squares' in the text.
- Fig.5: in absolute values, the Radratio (also the Rgratio) are very small and may correspond to relative changes of 10-20 % compared to the average. Can the authors comment? Do we expect an anticorrelation between Radratio and Rgratio ?
- Fig. 5D: regarding the previous point, the scheme seems a little bit exaggerated, moreover in Fig.5, the speckle aspects are not mentioned.
- Fig.6A: there is no blue line (while it is present in panel C)
- Fig.7C: how speckle distances are inferred from MERFISH?
- L452-453 "These observations …": this is an overstatement: authors show correlation but not a causal relation.
- Fig.8C: what are 'left 2' or 'left 4' loci ?
- Tables in the main text may be relocated in the Supplementary Material.
- Both the conclusion and discussion parts contain very similar summaries of the work, this is very repetitive, one is enough (see also remark on the end of the introduction).
- L859: refs [17,51] are repeated twice.
- L893: "multiples of 50 by bilinear interpolation": unclear.
- About the different layers used for the auto-encoder: is it standard? if yes, please add references, if not what is the rationale behind these choices?
- L1009: "can be we are able to find": typo
- L1061: "is structure": typo
- L1156: "The peaks of the insulation…": what is the algorithm use for peak detection?
- L1200-1214: the CIN, SAF and Markov clustering are never mentioned in the main text. It is unclear why there are materials and methods subsections for that.
- L1303: "contact range 2": unclear.
- L1308-1311: the rationale behind the specific choice is unclear (eg, theshholds 5 and -1)

**Authors’ response**

Reviewer#1:

The authors explore a method for examining variations in chromosome conformations among cells and investigate the connections between chromosome conformations and gene function. I find this to be a timely and valuable subject, and the content of the manuscript very interesting. Single-cell genomics and multiplexed imaging have unveiled significant stochastic variabilities in genome structures among individual cells. This stochasticity hampers our ability to establish a causal relationship between genome structures and gene function, presenting a notable challenge in classifying entire chromosome structures. The authors address this challenge by introducing a two-step dimension reduction method. They determine that almost half of all single-cell chromosome structures can be categorized into 5 to 10 prevalent chromosome morphologies, each characterized by distinct interaction patterns and chromosome territory domains. About half of the boundaries are located at syntenic breakpoints. Notably, the authors discover that a majority of these boundaries are conserved across different cell types. They validate their findings using data from RNA- and DNA-MERFISH imaging, along with single-cell sci-HiC data. I think that the results of the manuscript are sound and valuable. I have only minor comments.

We thank the reviewer for his/her supportive and encouraging comments.

Minor concerns:

1. I would suggest a more comprehensive discussion of how this work relates to the outcomes of principled polymer models of chromatin domain formation, such as the loop extrusion and String&Binders Switch models.

Following the reviewer’s suggestion we now extended the introduction to better relate to the outcomes of polymer models and added three references. We want to stress that the paper is not about simulation methods, but clustering of structures that may be generated from deconvolution-based modeling, from polymer modeling or from sequential or multiplexed FISH imaging. (Pages 3 to 4, lines 59 to 62.)

“Moreover, polymer simulations have observed transitions between open and more globular conformations of chromosomal regions, as for instance a dumbbell-like conformation for a 2Mb segment of human chromosome 2.” [1,2]. We also extended the discussion session to relate our outcomes to polymer simulations. (Pages 24 to 25, lines 577 to 583.):

“It was recently shown that downregulation of cohesin loaders leads to a more compact chromosome conformation in vivo [3]. It will be interesting to test in future under which conditions polymer simulations could reproduce the observed territory domains at predicted locations. For instance, it would be interesting if loss of CTCF loop extrusion barriers at specific regions would favor macrodomains at specific sites, or if specific block polymer states could similarly reproduce the observed territory domains.”

2. While I believe the authors' approach is robust to changes in the parameters of their dimension reduction method, I would recommend addressing this aspect in the manuscript. We agree with the reviewer that the hyper-parameters play an important part in the dimension reduction method, which are selected based on previously related references. The involved hyper-parameters include numbers of neural network layers, kernel sizes and channels of each layer. These parameters basically affect the dimension of the latent space. We test the algorithm with different choices of hyper-parameters and the detected clusters are very similar. For example, for Chr6, the results are reconstructed well when the latent dimension ranges from 512 to 2048. However, using too high a latent dimension can lead to the curse of dimensionality, making it difficult to embed by t-SNE, while a too small latent dimension may result in latent vectors that fail to fully capture the input information, potentially leading to missing clusters. We now provide an extended discussion also on the robustness of our results when varying the population size of chromosome structures, the resolution of the input chromosome structures, as well as changes in the parameters used in the clustering algorithm (see also reviewer #2.1).

To better assess the robustness of our results with a reduced number of chromosome structures, we expanded **Additional file 1: Fig. S5** in the revised manuscript, here presented as **Figure R1A**. The updated figure now includes projected conformational spaces (**Fig. R1A**), average cluster distance matrices (**Fig. R1B**), in addition to the number of detected clusters. We show that we can observe almost identical clusters when the number of input structures is at least 15,000. The number of detected clusters decreases with decreasing number of input structures. Low-occupancy clusters go undetected because identifying local density maxima through kernel density estimation requires an adequate number of structures. However, clusters with higher occupancy are detected with almost identical average distance matrices, showing the robustness of our method even at lower chromosome structure numbers. For instance, for 10,000 input structures the method still predicts correctly 7 out of 8 clusters, while at 5000 input structures, the method detects 5 out of 8 clusters (**Fig. R1B**) Furthermore, we now tested the robustness of our method with respect to the resolution of chromosome structures. To do this, we down sampled all chromosome structures to 3Mb resolution, meaning chromatin regions were sampled at intervals of only 3Mb. Despite the substantially lower structural resolution, we can still identify 6 out of 8 clusters detected in our original 200kb resolution chromosome structures, with very similar contact patterns (**Fig. R2**). Moreover, we previously showed that we could detect the same clusters for structures at low 3Mb resolution from multiplexed FISH imaging (**Additional file 1: Fig. S8** in the SI of the original manuscript (now **Additional file 1: Fig. S13** in the revised manuscript)). Following the suggestion of reviewer 3, we also generated a positive and negative control population of structures to assess the robustness of our approach. We generated a negative control consisting of 20,000 genome structures modeled as random self-avoiding chromosome homopolymers (without Hi-C restraints) constrained only by nuclear volume. As anticipated, the negative control failed to produce our observed clusters (**Fig. R3**). As a positive control we created several datasets consisting of chromosome structures derived from average cluster distance matrices, with varying levels of random noise sampled from a Gaussian distribution (standard deviation ranging from 0.1 to 0.8). Our method successfully separated all data points in the conformation space and accurately clustered all structures from each positive control dataset into their correct clusters, regardless of the noise levels (**Fig. R4**)

We now added a whole new section at the beginning of the results section **“Assessing robustness of clustering method.”** and added two new figures as **Additional file 1: Fig. S6AB, S7** in the revised manuscript.


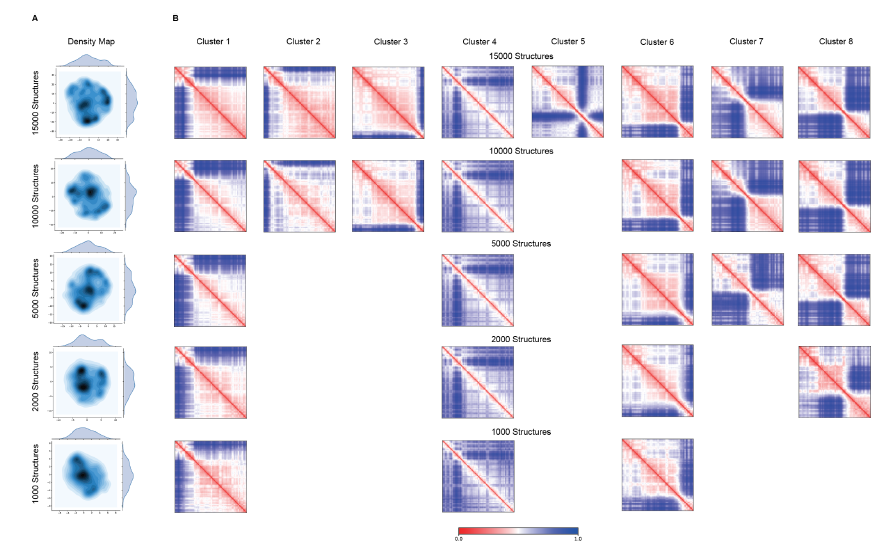


**Fig. R1: Cluster analysis for results of different numbers of input A,** Density distribution of the whole population shown in the conformation space for inputs with decreasing numbers of structures. **B,** Distance matrices of the clusters identified for each number of inputs. We observe that fewer clusters are identified when reducing the input size, but clusters are still among those found in results on 20,000 structures. Clusters are labeled with the same cluster indices from the original analysis.


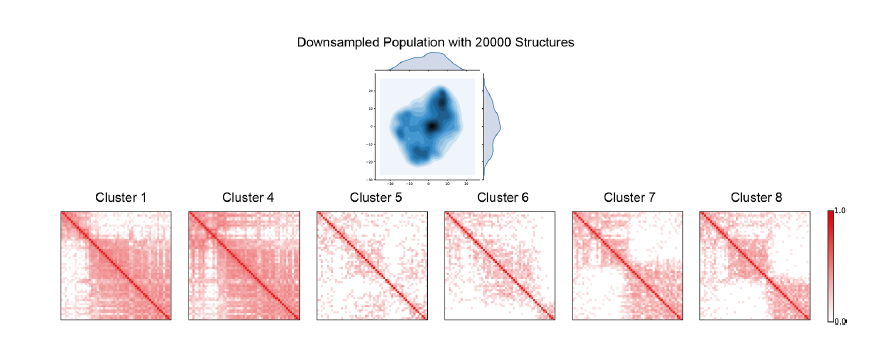


**Fig. R2: Cluster analysis for different negative control populations with 20,000 inputs** (Top row) Density distribution of the downsampled population of structures at 3Mb. Clusters are labeled with the same cluster indices from the original analysis. (Second row) Contact matrices of clusters identified from the downsampled population at 3Mb.


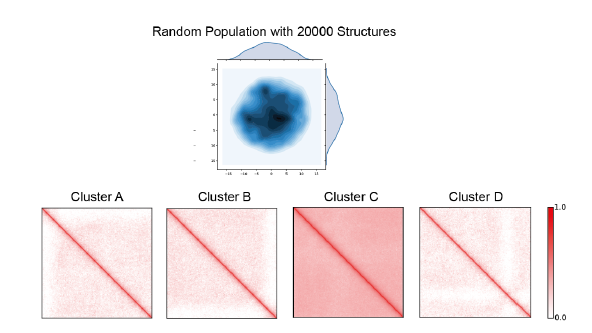


**Fig. R3: Cluster analysis for different negative control populations with 20,000 inputs** (Top row) Density distribution of the random population generated without Hi-C restraints. (Second row) Contact matrices of clusters identified from the random population generated without Hi-C restraints.


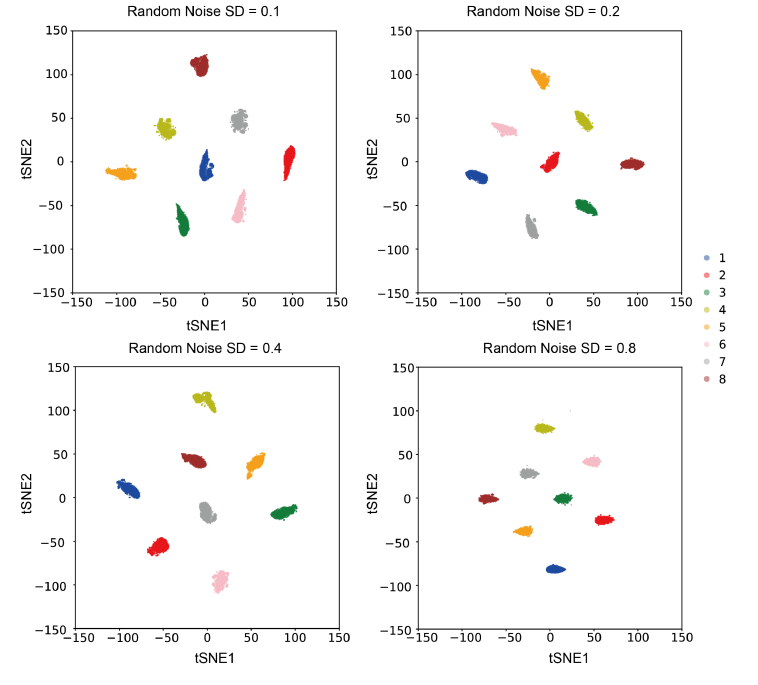


**Fig. R4: Positive control analysis.** Chromosome structures are derived from average distance matrices in a cluster by adding random noise sampled from a Gaussian distribution with specific standard deviation (SD) ranging from 0.1 to 0.8. Shown are the clusters derived from the positive control data sets by our two-step dimension reduction approach. We find that our method clearly separates data points generated from the 8 different clusters. Points are colored according to their ground truth cluster indices from which they are generated

Reviewer #2:

Major concerns:

1. The first question is, how robust is the method? The authors started with 20,000 chromosome structures and ended with 25%-50% of these structures can be clustered into 5-12 subpopulations. What happened when the authors started with variable numbers of chromosome structures (e.g., 10, 000, 15,000, 25,000, or 30, 000, etc)? Also, when run the experiment multiple times, are the results reproducible?

As suggested by the reviewer we now provide an extended discussion on the robustness of our results with varying population size of chromosome structures (see also response to reviewer #3.2b). We find identical clusters when the number of input structures is at least 15,000 (**Fig. R1B**). The number of detected clusters decreases with a lower number of input structures. Low-occupancy clusters go undetected because identifying local density maxima through kernel density estimation requires an adequate number of structures. However, clusters with higher occupancy are detected with almost identical average distance matrices, showing the robustness of our method even at lower chromosome structure numbers. For instance, for 10,000 input structures the method still predicts correctly seven out of eight clusters, while at 5000 input structures, the method detects five out of eight clusters (**Fig. R1B**). At 2000 structures we detect four and with 1000 structures three clusters (**Fig. R1B**). To clarify these observations and the robustness of our method we now expand **Additional file 1: Fig. S5** in the revised manuscript, here presented as **Figure R1**. The updated figure now includes the projected conformational spaces (**Fig. R1A**), average cluster distance matrices (**Fig. R1B**), in addition to the number of detected clusters.

We now added a new section in the revised manuscript to describe these assessments (Page 9, lines 186-201) and extended a supplementary figure (**Additional file 1: Fig. S5**). The updated figures not only contain the number of detected clusters, but now also includes the detected projected conformational phase spaces, cluster average distance matrices / average contact frequency matrices for analysis performed for 15,000, 10,000, 5000, 2000, and 1000 structures. The maximum number of structures available to us is 20,000. Following the reviewer’s suggestion we also highlight that our results are fully reproducible when running our method multiple times. At each run, we randomize the order of the input matrices to the autoencoder, and also randomize the locations of all embedded data points at the beginning of t-SNE. Besides that, the whole process is deterministic and no other random information is included. Hence, the original input configuration does not affect the results. We now added these details about the robustness of our method with respect to randomization of input data in the methods part. (Pages 9 to 10, lines 207 to 215.)


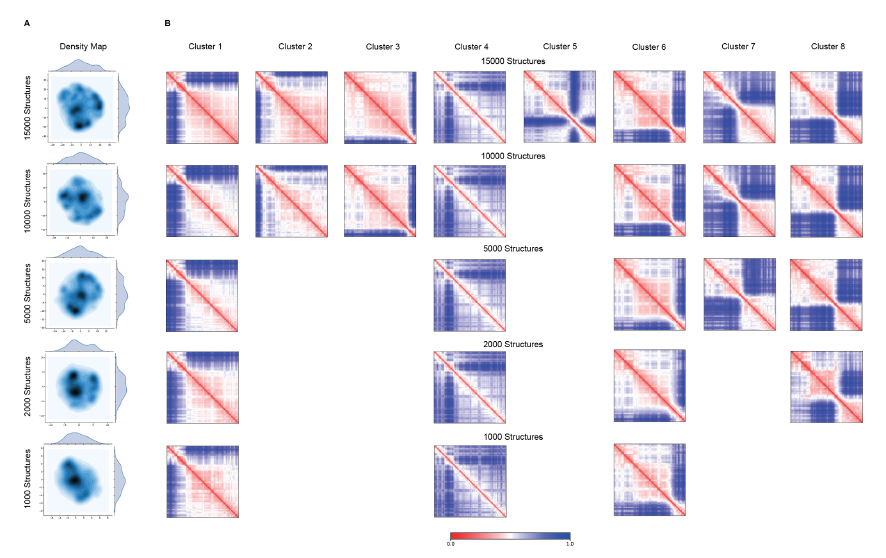


**Fig. R1: Cluster analysis for results of different numbers of input A,** Density distribution of the whole population shown in the conformation space for inputs with decreasing numbers of structures. **B,** Distance matrices of the clusters identified for each number of inputs. We observe that fewer clusters are identified when reducing the input size, but clusters are still among those found in results on 20,000 structures. Clusters are labeled with the same cluster indices from the original analysis.

2. Why only 25%-50% of the chromosome structures can be clustered into a few subpopulations? Does it because, in nature, there is no common features/characteristics among the rest of the chromosome structures?

Our method detects dominant conformational clusters with relatively high occupancy, meaning structures observed at relatively high probability in the cell population. To investigate the nature of more rare chromosome conformations outside detected clusters, we analyzed chromosome conformations between clusters in the projected phase space (**Additional file 1: Fig. S9**) (shown as **Fig. R5** here) in the original manuscript. We choose several trajectories in the projected phase space connecting two local maxima (cluster centers) and select at four equidistant locations 100 nearest neighbors representing intermediate conformations along this path. We then calculate the average distance matrices of these 100 nearest neighbors at the corresponding location. We show that these distance matrices represent intermediate transitions between the cluster conformations. This is shown by the gradual changes in Pearson correlations between average distance matrices of the clusters and the intermediate conformations (**Additional file 1: Fig. S9**). Conformations representing transition structures have relatively lower probability in the structure population and are therefore not detected as dominant clusters. We now updated **Fig. S9 in the revised manuscript and describe these observations in the text.** (Page 10, lines 224 to 227.) (for reference: “Unclustered structures located midway between two cluster centers…”.)


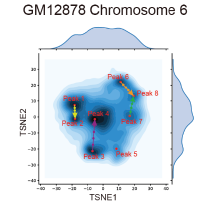


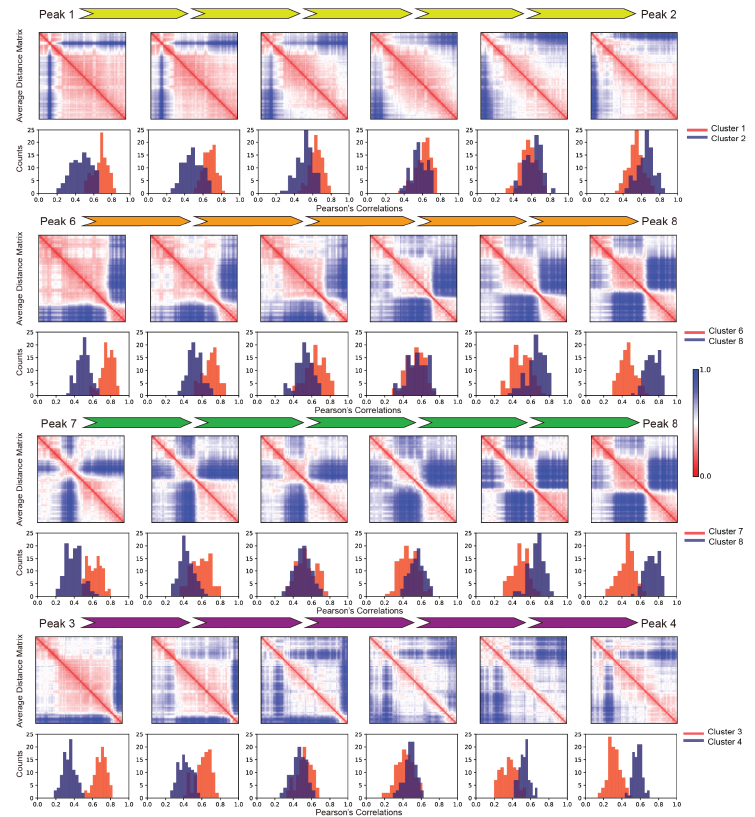


**Figure R5: Intermediate conformation analysis on GM12878 Chr6.** Various paths in different colors are chosen to select intermediate conformations from one peak (local maximum) in the projected conformational phase space to another peak (local maximum). Between every two peaks (red dots), four locations (dots in certain color) are selected equidistantly along the path (arrow in certain color) for conformation sampling. Each distance matrix is calculated by average of the 100 nearest neighbors of the corresponding location. Also shown below the matrix is the histogram of Pearson’s correlations between single-cell distance matrices of the 100 structures and both cluster average distance matrices to which the two peaks belong.

3. The authors said that the main difference between the 5-12 chromosome structure subpopulations lies in their long-range (>10 Mb sequence distance) chromatin interaction patterns, does it mean that all these subpopulations have similar short-range (<= 10 Mb) interaction patterns? Or because the resolution of the method is not sufficient to distinguish the differences in the short-range (<= 10 Mb) interaction patterns?

The dominant differences in chromosome conformations between clusters are due to long-range interaction patterns >10Mb. To make this point clearer we now also provide the contact probability between chromatin regions with sequence distance lower than 10Mb (**Fig. R6**). We see almost identical decay rates of contact probabilities between all clusters, which proves further that long-range interactions play a more crucial role than short-range in determining cluster differences. In contrast, contact probabilities for sequence distances larger than >10Mb differ substantially between clusters as shown in **Fig. 2F** in the revised manuscript. The resolution of our structures is sufficient to detect local structural features. However, the dominant conformation differences are detected at longer-range levels, and some local very short-range structural features are likely uncoupled from the larger structural variations.


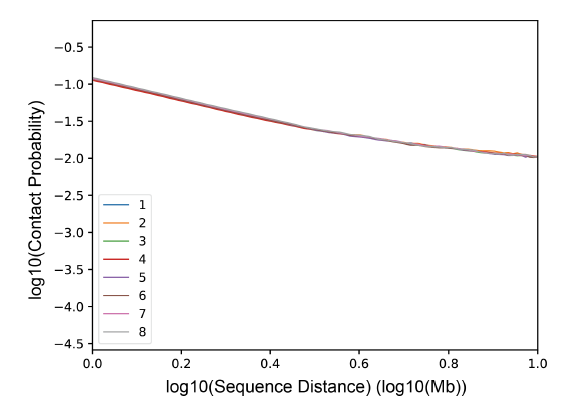


**Fig. R6: The log fold change of contact probability against log fold change of sequence distance at scales smaller than 10 Mb.** We observe an overall decrease in the contact probability over the sequence distance, indicating the same trend as the scale larger than 10 Mb. However, there are nearly no differences between the patterns of different clusters.

4. With respect to the interplay between the different levels of the chromosome morphological features, nuclear compartments, and gene transcription, it's hard to conclude as causal relationship without biological validation.

We agree with the reviewer that it is hard to conclude the causal relationship between

chromosome structure and gene function without biological validation. Using DNA-MERFISH

data [4] we did show a correlation between morphological features and expression for the

ensemble or specific clusters. However, to avoid confusion, we now clearly stated in the

manuscript that our analysis does not prove a causal relationship, which would require other

biological validation experiments. We added the following statement: (Page 21, lines 489-492.)

“These observations indicate that variations in chromosome conformations correlate with gene

transcription levels, possibly through variations in the specific nuclear locations of genes in

different conformations. However, further experimental evidence is required to establish a

definitive causal link between chromosome conformation and gene expression.“

5. The details about territory domain boundaries need to be further established/clarified. What

is the size of these boundaries? Do they exist in every chromosome structure? Or can they

only be identified in each of the subpopulations? Are their genomic location subpopulation

specific? Etc.

We agree with the reviewer and now better clarify how domain boundaries are detected using

an insulation score method (defined in methods) (See also reviewer #3, point 3.2e). We

apologize for not making this point clearer. We now provide more details on how domain

boundaries are determined by an insulation score calculated from average distance matrices in

each cluster. The method uses a sliding window approach to calculate an insulation score for

each genomic region. Specifically, we determine the ratio between average distance matrices in

a window centered at the target region and the product of the average distance matrices for

subwindows upstream and downstream from the target window (Methods). To detect domains

across different scales, we first use a larger window size (40MB) to detect approximate locations

of domain boundaries in each cluster, then we use a smaller window size (6Mb) to detect their

specific locations. The largest peak regions (local maxima) of the insulation score profiles were

detected by the package detecta (https://github.com/demotu/detecta.). These peaks correspond

to candidates for territory domain boundaries (Fig. R7). In addition, domain boundaries typically

align with major shifts in the average radial position profiles (RAD) at the detected peak regions.

As for instance for peaks indicated by the two rightmost arrows in cluster 8, which define

domain p (between both rightmost arrows) as a separate territory domain (Fig. R7 and 5A).

Moreover, domain boundaries are also confirmed by local chromatin fiber decondensation, as

defined by the local radius of gyration (RG) of the chromatin fiber (defined as the radius of gyration of a 1Mb window centered at the target region). Domain boundary locations often align

with peaks in the chromatin fiber decondensation (**Fig. 5A**).

Although the insulation score profiles are calculated from average cluster distance matrices,

boundaries can also be detected in single chromosome structures (**Fig. R8**). **Fig. R8** shows

examples of single-cell distance matrices from different clusters whose single cell insulation

scores reveal local peaks at the locations of detected domain boundaries in the cluster

averages. However, due to the high variability of patterns at single-cell level, the detection of

domain boundaries is more challenging.

We added this explanation in the revised manuscript page 11 lines 244 to 247 and added two

new supplementary figures (**Additional file 1: Fig. S10, S11** in the revised manuscript).


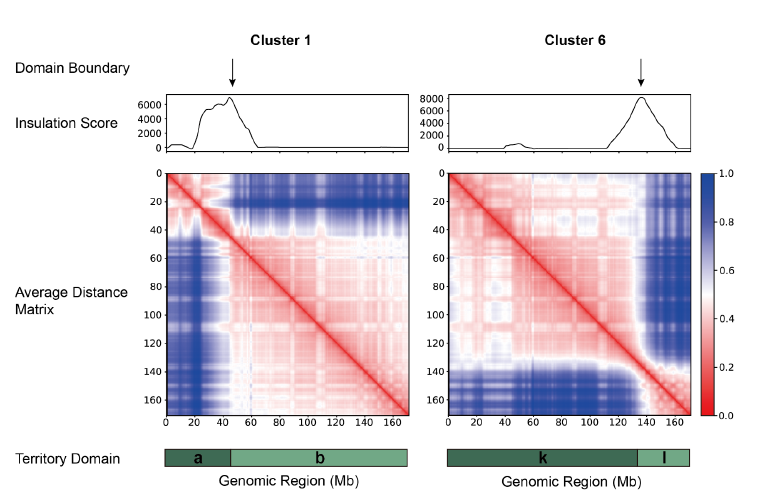


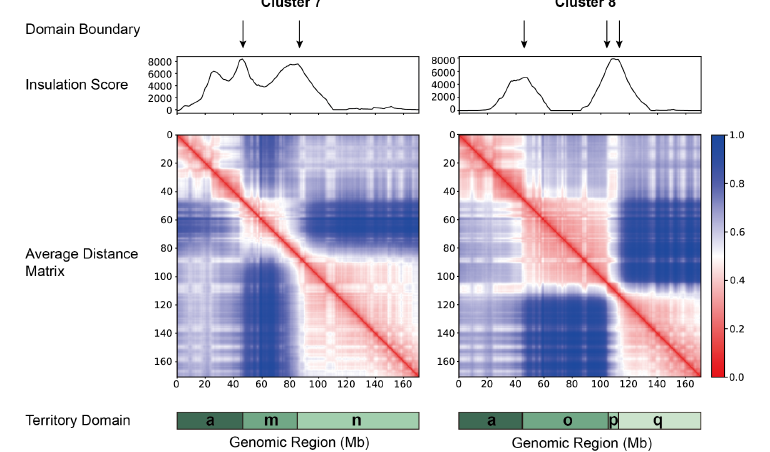


**Fig. R7: Domain boundary analysis for selected clusters from GM12878 Chr6** (From top to the bottom) The first row shows the insulation score calculated from the average distance matrix. The second row shows the average distance matrices of clusters. The third row indicates the territory domains separated by the domain boundaries.


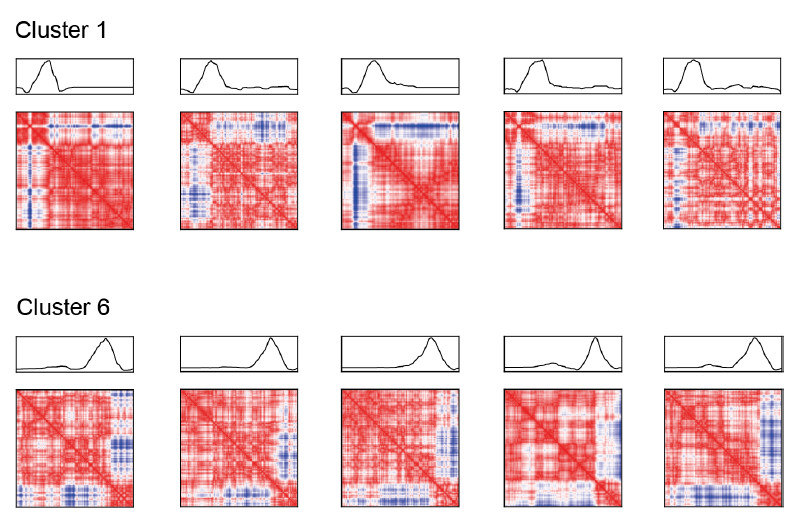


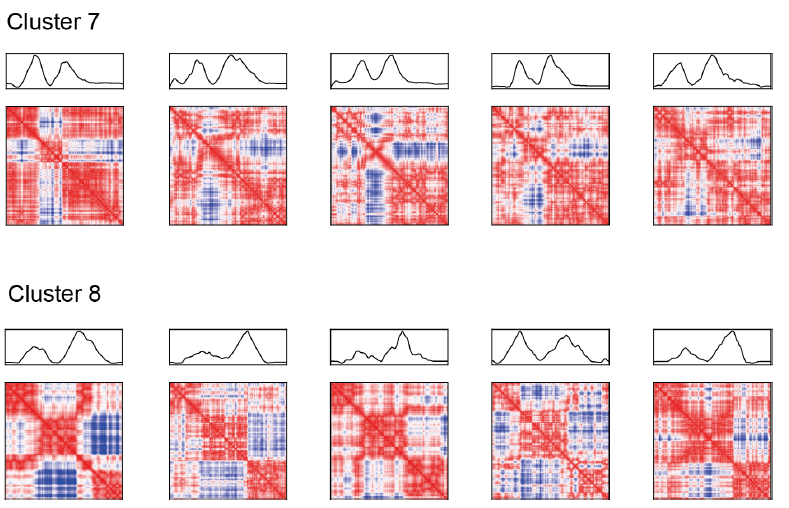


**Fig. R8: Selected single-cell examples for each cluster found in GM12878 Chr6** We find that single-cell distance matrices in each cluster show high similarity in large-scale conformation and territory domains which can be detected by the insulation score profile (shown on top of each distance matrix), although variability and flexibly distributed conformations are observed at small scale.

Minor concerns:

1. In this sentence (page 3 line 60-62), "and TAD domain boundaries are only rarely observed at the ensemble average position but are rather stochastically distributed, because of dynamic loop extrusion processes [19,34,36,37].", "rarely observed" is not appropriate, because TAD is one of the 3D features that can be regularly observed in bulk Hi-C assays.

We apologize for this misunderstanding. The sentence is rephrased as "and TAD domain boundaries are rarely observed in single cells at the exact ensemble locations but are rather stochastically distributed, because of dynamic loop extrusion processes." (Page 3, lines 46 to 48.)

Reviewer #3:

Zhan et al propose a method to cluster single-cell chromosome conformations into 'structural' clusters. Applying this method to structures inferred from population Hi-C map using a data-driven - previously published - approach (IGM), authors show that the clusters are characterized by different contact patterns and domains, and are compatible with single-cell experimental data (MERFISH and sc Hi-C). Then they analyze the characteristics of the clusters and show that they are associated with differential radial positioning, inter-chromosomal contacts, gene expression, etc. Finally, they show some degree of conservations of the domains inferred from different cell types. Overall, the work is interesting and properly done. In particular, the proposed clustering method seems promising even if its direct application to single-cell experimental data remains to be demonstrated. However, I found the rest of the paper very descriptive and the novelties in terms of new generic concepts on chromosome 3D organization or of specific biological contexts are unclear. In particular, the overall relation between 3D structure, condensation, radial positioning, gene expression at the single cell level was already characterized by the same authors in previous publications (and other authors also) (without the notion of clusters - which is new here - but the main conclusions seem very similar). The possibly interesting discovery of some genes being differentially expressed depending on clusters should be confirmed experimentally and characterized more deeply. The more original parts on syntenic blocks and comparisons between cell types is very sparse.

Major concerns:

1. I would suggest the authors - in addition to other concerns (see below) - to clarify the novelties of their results and develop more the last two parts of their work.

We thank the reviewer for the careful evaluation and valuable suggestions. We now have made efforts to better highlight the novel aspects of our method and findings. This study establishes, for the first time, a connection between single cell chromosome conformations, with gene function, and subnuclear locations of genes within the nuclear environment. This has not been achieved previously due to two key challenges: (1) the difficulty of classifying highly dynamic chromosome structures and (2) the lack of comprehensive analysis of chromosome conformation that integrates both genome architecture and the spatial locations of nuclear bodies. To our knowledge, no method currently exists that classifies chromosome structures into dominant conformational states. It has not been demonstrated so far that almost half of all chromosomes can be observed in several dominant conformational states, which are distinguished by the locations of territory domain boundaries that divide the chromosome territory domains into spatial subunits. Most of these boundaries are unique to specific conformational states. In the future, we plan to investigate the factors contributing to the formation of these boundaries in specific conformations. Determining causal relationships between specific features and boundary formation will require further experimental studies, which are beyond the scope of this paper. More importantly, our results demonstrate that specific chromosome conformational states are associated with the specific subnuclear positioning and functional properties of certain genes. By linking chromosome conformational states with functional properties, our study opens new avenues for a better understanding of cell-to-cell heterogeneity of chromosome structures and gene expression. Following the reviewer’s suggestion we now expand the last two parts of our work, namely the sections “Chromosome morphologies influence gene functions” and “Characteristic features of chromosome territory domain boundaries”.

To gain further insights into how chromosome conformations influence gene function, we calculated A/B compartments in each cluster by eigenvector decomposition of its contact frequency matrix following the approach in Rao et al [5]. Interestingly, we observe considerable differences in the A/B compartment annotation between different clusters, further supporting our conclusion that chromosomal conformations are linked to functional states. (Pearson’s correlations to the ensemble PC1 profile range between 0.72 and 0.99). For example, the terminal region of the q-arm on chromosome 6 (155 to 171 Mb, region **III** in **Fig. 7E** in the revised manuscript) changes from the B compartment in clusters 1, 2, 4, 5, 6, and 8 to the A compartment in cluster 3. This change is consistent with the observed increased transcriptional activity of genes in region **III** and greater speckle associations of this region in cluster 3, compared to other clusters (**Fig. 7E** in the revised manuscript). This observation supports our conclusion that chromatin's functional properties can vary when a chromosome adopts different conformational states—an important finding that has not been previously reported. Similarly, we observe a marked increase in eigenvalues values, indicating stronger A compartment associations for region **I** in cluster 1 compared to all other clusters. This again aligns with our observed higher transcriptional activity and closer speckle associations of these genes in cluster 1 (**Fig. 7E** in the revised manuscript). Additionally, Region **II** in cluster 8 shows elevated eigenvalues (PC1) and increased A compartment association relative to clusters 1 and 3, which is also consistent with the observed higher transcriptional activity of these genes in cluster 8 (**Fig. 7E** in the revised manuscript). We now added a new Figure in the supplementary information (**Additional file 1: Fig. S16**) (shown as **Fig. R9** here) showing the PC1 and PC2 component profiles calculated from the contact frequency matrix of each cluster. We also extended **Figure 7EF** (Shown as **Figure R10** here) in the revised manuscript, to highlight the agreement between changes in the A/B compartment annotations and gene transcription for chromosomes in clusters 1, 3, and 8.

We added a new section discussing the A/B compartment annotations of clusters on pages 17 to 18, lines 393 to 414. Interestingly, the first PC1 component (the eigenvector corresponding to the largest eigenvalue) typically corresponds to A and B compartment patterns, as is the case for clusters 1, 2, 3, 4 and 5. However, for clusters 6, 7 and 8, the PC2 component correlates with A-B compartments, while the PC1 component correlates with territory domain locations (**Fig. R9**), confirming the locations of our domain boundaries for these clusters. This may be explained by the relatively large number of territory domains in these clusters combined with large structural changes at domain boundaries (ie., radial transitions), in particular for clusters 7 and 8. Our observations agree with a recent study in mouse cortex brain cells [6], where PC1 components correlated with chromosomal megadomain structures in some neuronal cells, while the PC2 component correlated with A/B compartments. We added a new figure in the supplementary information to demonstrate the connection between PC1 eigenvalues and presence of territory domains for cluster 6, and 8 (**Additional file 1: Fig. S12**, here shown as **Fig. R11**).


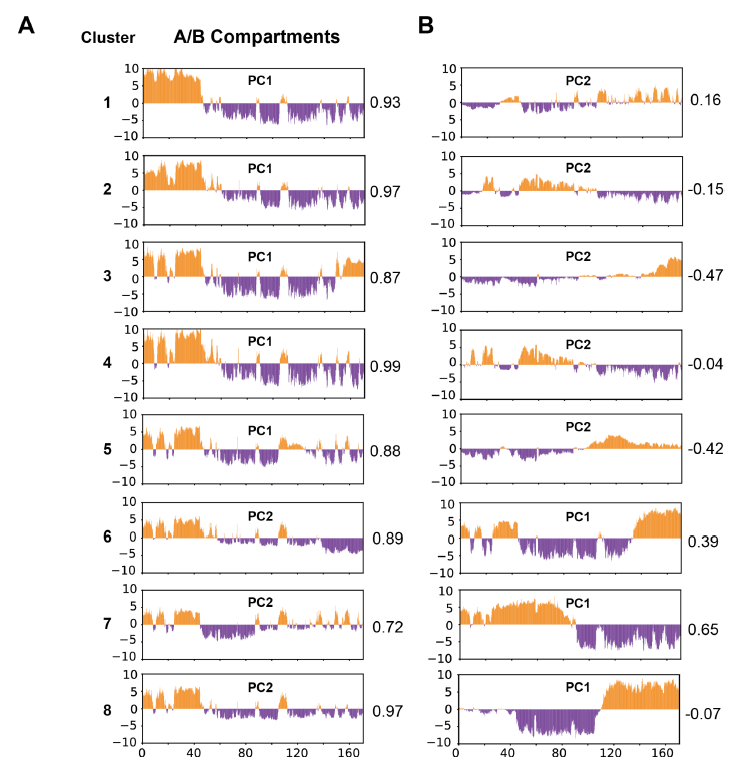


**Fig. R9: A/B compartments by principal component analysis on cluster contact frequency matrices of chromosome 6. A,** A/B compartment profiles for chromosomes in each cluster is shown together with Pearson's correlation with the A/B compartment profile calculated from the full ensemble. For clusters 1-5 the compartment profile corresponds to the first eigenvalue (PC1) of the PCA analysis. For clusters 6-8 the compartment profile is represented by the second eigenvalue (PC2). For these clusters the first eigenvalue corresponds to the territory domain segmentation. **B,** The second largest eigenvalue (PC2) profile for clusters 1-5 and the largest eigenvalue (PC1) profiles for clusters 6-8 together with the lower Pearson’s correlation of each profile with the ensemble PC1 profile.


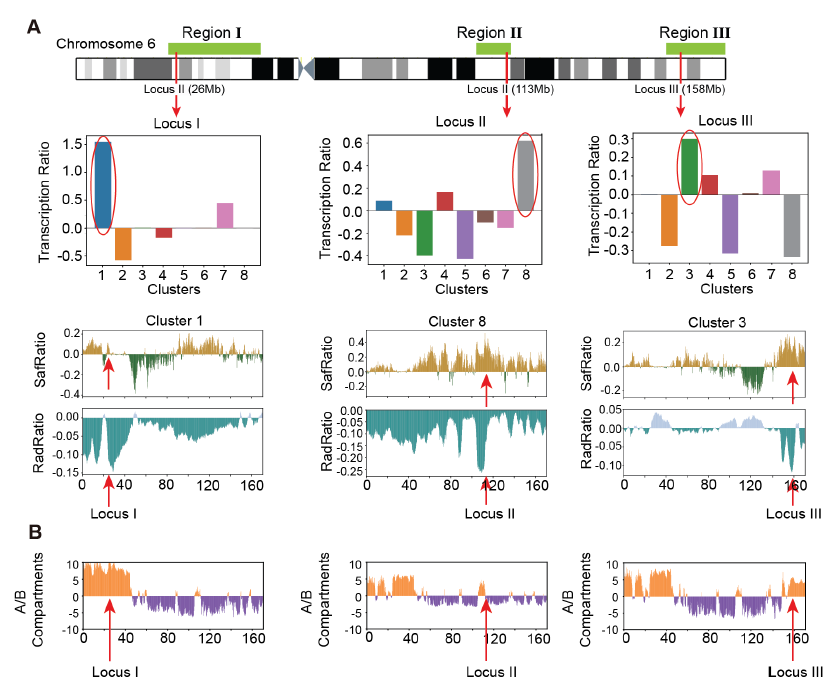


**Fig. R10 Potential linkage between chromosome morphologies and gene functions A,** (Top panel) The genomic positions of region **I** (24-48 Mb), region **II** (105-114 Mb) and region **III** (155-171 Mb) together with locus I (26 Mb), locus II (113 Mb) and locus III (158 Mb) on chromosome 6. (Second panel from top) The transcription ratio of the three selected locus I, II and III for all clusters measured by DNA-MERFISH [4] (Methods) (Third panel from top) SafRatio of cluster 1, 8 and 3. (Fourth panel from top) RadRatio of cluster 1, 8 and 3. **B,** A/B compartments (PC1) of cluster 1, 8 and 3.


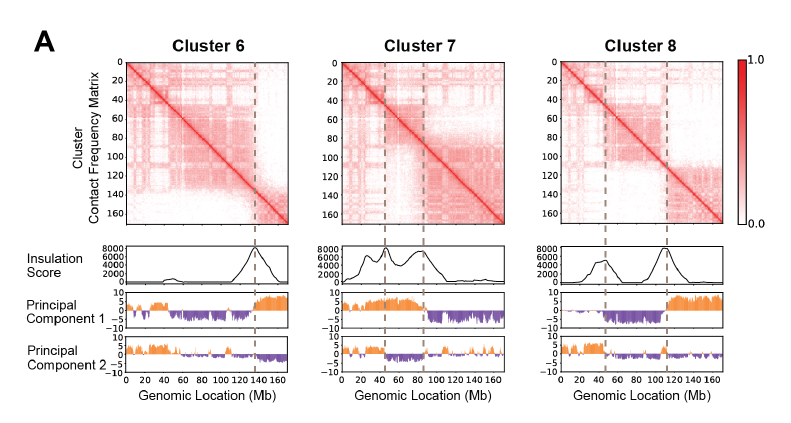


**Fig. R11: Analysis of structural features and regulation marks on territory domains A,** The principal component 1 (PC1) and the principal component 2 (PC2) showed below cluster contact frequency matrix for cluster 6, cluster 7 and cluster 8 from GM12878 Chr6. We find that PC1 basically correlates with the territory domains, while PC2 are related more with ensemble compartments.

2. On the approach/method:

a. Authors convincingly analyzed their approach and its consistency. However, I think it is missing a positive and a negative control where we know a priori the outcome to fully validate it. As a negative control, applications of the method to structures sampled from a simple homopolymer model (simple self-avoiding walk for example) may be used. As a positive control, a mixture of structures with clear differences (eg, presence or absence of loops) may be used.

We thank the reviewer for this suggestion. We now generated several control populations using IGM [7], including a negative control consisting of 20,000 genome structures modeled as random self-avoiding chromosome homopolymers (without Hi-C restraints) constrained only by nuclear volume. As anticipated, the negative control population does not produce the clusters observed in H1ESC cells but shows random contact patterns (**Fig. R3**). As a positive control we created multiple datasets consisting of chromosome structures derived from average cluster distance matrices, with varying levels of random noise sampled from a Gaussian distribution (standard deviation ranging from 0.1 to 0.8). Our method successfully clustered all structures from these positive control datasets into their correct clusters across all noise levels (**Fig. R4**), demonstrating its robustness (**Fig. R4**)**.** We now added a new section in the revised manuscript to describe these assessments (page 9 to 10, lines 207 to 215) and added two new supplementary figures (**Additional file 1: Fig. S6, S7** in the revised manuscript).


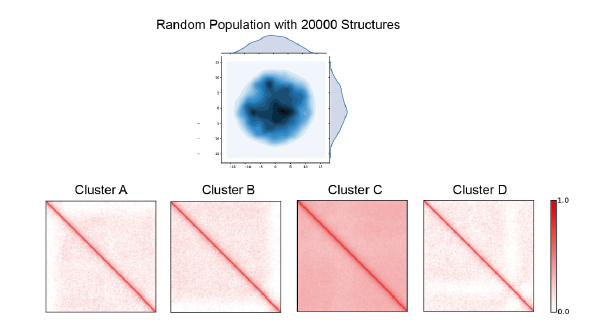


**Fig. R3: Cluster analysis for different negative control populations with 20,000 inputs B,** (Top row) Density distribution of the random population generated without Hi-C restraints. (Second row) Contact matrices of clusters identified from the random population generated without Hi-C restraints.


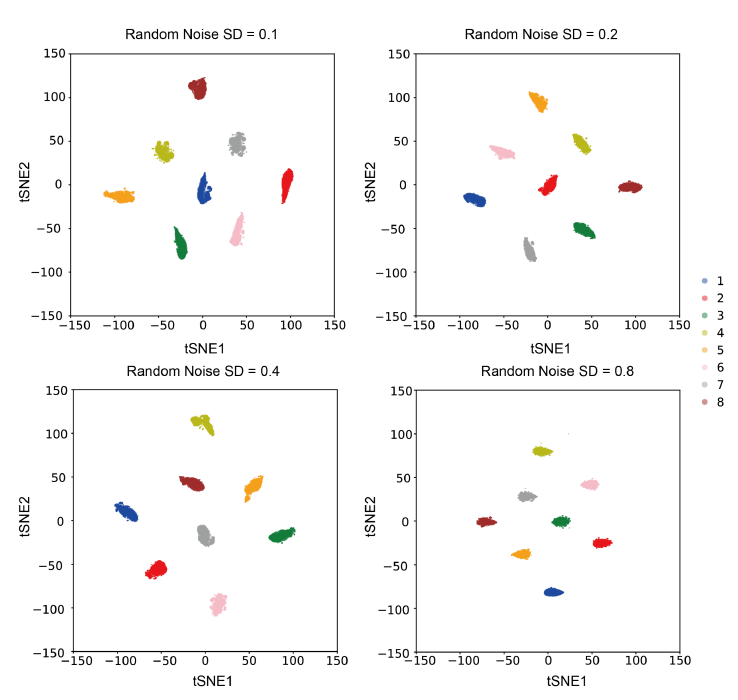


**Fig. R4: Positive control analysis.** Chromosome structures are derived from average distance matrices in a cluster by adding random noise sampled from a Gaussian distribution with specific standard deviation (SD) ranging from 0.1 to 0.8. Shown are the clusters derived from the positive control data sets by our two-step dimension reduction approach. We find that our method clearly separates data points generated from the 8 different clusters. Points are colored according to their ground truth cluster indices from which they are generated.

b. Fig.S2 (investigation of the role of the number of input structures): when fewer clusters are inferred with lower numbers of input structures, do these clusters "encompass" the 'new' clusters emerging with high number of inputs?

Following the reviewer’s suggestion we now provide an extended discussion on the robustness of our results with varying population size of chromosome structures (see also response to reviewer #2.1). We find identical clusters when the number of input structures is at least 15,000 (**Fig. R1B**). The number of detected clusters decreases with a lower number of input structures. Low-occupancy clusters go undetected because identifying local density maxima through kernel density estimation requires an adequate number of structures. However, clusters with higher occupancy are detected with almost identical average distance matrices, showing the robustness of our method even at lower chromosome structure numbers. For instance, for 10,000 input structures the method still predicts correctly seven out of eight clusters, while at 5000 input structures, the method detects five out of eight clusters (**Fig. R1B**). With 2000 input structures we detect four and with 1000 input structures we detect three clusters (**Fig. R1B**).

To clarify these observations and the robustness of our method we now expand **Fig. S5** in the revised manuscript, here presented as **Figure R1**. The updated figure now includes the projected conformational spaces (**Fig. R1A**), average cluster distance matrices (**Fig. R1B**), in addition to the number of detected clusters. We now added a new section in the revised manuscript to describe these assessments (page 9, lines 186 to 201) and extended a supplementary figure (**Additional file 1: Fig. S5**).


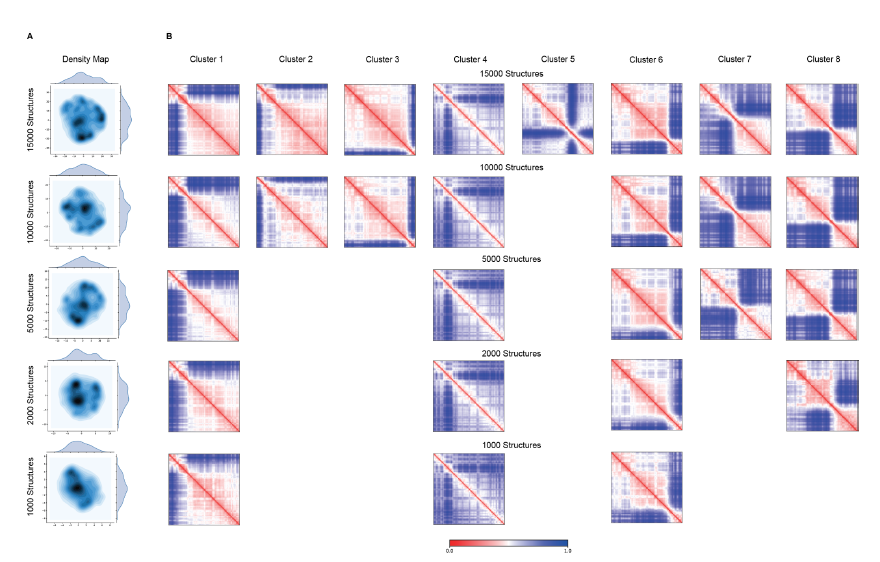


**Fig. R1: Cluster analysis for results of different numbers of input A,** Density distribution of the whole population shown in the conformation space for inputs with decreasing numbers of structures.. **B,** Distance matrices of the clusters identified for each number of input. We observe that fewer clusters are identified when reducing the input size, but clusters are still among those found in results on 20,000 structures. Clusters are labeled with the same cluster indexes from the original analysis.

c. How downsampling (or more generally the resolution) is affecting the clustering?

Following the reviewer’s suggestion, we tested the robustness of our method with respect to the resolution of the chromosome structures. We downsampled all chromosome structures to 3Mb resolution, meaning chromatin regions were sampled at intervals of only 3Mb. Despite the substantially lower structural resolution, we can still identify six out of eight clusters detected in our original 200kb resolution chromosome structures, with very similar contact patterns (**Fig. R2**). Moreover, we previously showed that we can detect the same clusters for structures at low 3Mb resolution from multiplexed DNA-MERFISH experiment [4] (**Additional file 1: Fig. S8** in the SI of the original manuscript (now **Additional file 1: Fig. S13**)). We added a paragraph in the revised manuscript to describe these assessments (page 9, lines 202-206) and extended a supplementary figure (**Additional file 1: Fig. S6**).


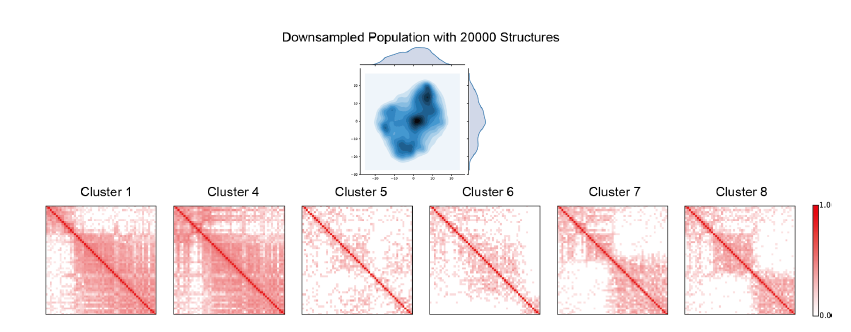


**Fig. R2: Cluster analysis for different negative control populations with 20,000 inputs** Density distribution of the downsampled population of structures at 3Mb. Clusters are labeled with the same cluster indices from the original analysis. **B,** Contact matrices of clusters identified from the downsampled population at 3Mb.

d. The majority clusters (eg, cluster 4 for chrom 6) for each chromosome seem always the ones with more compact structures, can the authors comment?

Yes, this is true for almost all chromosome examples and it is confirmed in experiments from both DNA-MERFISH and single cell sci-HiC [8]. Also in experimental data the compact conformations are found at higher frequency than more extended conformations. We can only speculate why this is the case. It may be possible that extended chromosome conformations are formed over time from more compact conformations. In cycling cells this may lead to a larger fraction of cells in a more compact formation as chromosomes exiting mitosis are in a more compact conformation. We noticed in a recent study that fully differentiated neuronal cells have more extended chromosome conformations [6].

e. It is unclear how domains are determined and how boundaries are defined for each cluster in the main text. Can the domain boundaries be inferred directly from the original population Hi-C matrix ?

We agree with the reviewer and now better clarify how domain boundaries are detected using an insulation score method (defined in the Methods section) (See also response to reviewer 2, point #2.5.). We apologize for not making this point clearer and we now provide more details on how domain boundaries are determined by an insulation score calculated from average distance matrices in each cluster. The method uses a sliding window approach to calculate an insulation score for each genomic region. Specifically, it uses the ratio between average distance matrices in a window centered at the target region and the product of the average distance matrices for subwindows upstream and downstream from the target window (Methods). To detect domains across different scales, we first use a larger window size (40MB) to detect approximate locations of domain boundaries in each cluster, then we use a smaller window size (6Mb) to narrow down their specific locations. The largest peak regions (local maximas) of the insulation score profiles were detected by the package detecta (https://github.com/demotu/detecta). These peaks correspond to candidates for territory domain boundaries (**Fig. R7**). Moreover, domain boundaries are also assessed based on the local radius of gyration (RG) of the chromatin fiber and transitions in the average radial position profile (RAD). For instance, domain boundary locations often align with peaks in the chromatin fiber decondensation (defined as the radius of gyration of a 1Mb window centered at the target region) (**Fig. 5A**). In addition, domain boundaries also typically align with transitions in the average radial position profiles (RAD) at the detected peak regions. (As for instance for peaks pointed by arrows in cluster 8, which define domain p as a separate territory domain (**Fig. R7 and 5A**). **Fig. R7** shows examples of insulation score profiles of clusters 1, 6, 7 and 8 aligned with the corresponding distance matrices and locations of domain boundaries. **Fig. R8** shows single-cell insulation scores together with single-cell distance matrices, which indicates domain boundaries can also be discovered at single-cell level. Although the profiles are calculated based on average matrices, they can also be detected in single chromosome structures (**Fig. R8**). **Fig. R8** shows examples of single-cell distance matrices from different clusters whose single cell insulation scores reveal local peaks at the locations of detected domain boundaries in the cluster averages.

We added this explanation in the revised manuscript (page 11 lines 244 to 247) and added two new supplementary figures (**Additional file 1: Fig. S10, S11** in the revised manuscript). Concerning the reviewers question: “Can the domain boundaries be inferred directly from the

original population Hi-C matrix ?“

No, not all boundaries can be detected, nor are all boundaries present at the same time. Boundaries are single cell specific, and only detected when single cells are clustered into conformational states. There are some boundaries that are present in multiple clusters, such as boundary 1 forming domain a, which is present in clusters 1, 7 and 8. However all other boundaries are specific to individual clusters. Therefore, we cannot infer all boundaries from the ensemble Hi-C matrix at once, since domain-like patterns are averaged out in the ensemble. Moreover, our distance-based insulation score is not able to detect clear differences for long range domain patterns from the average distance matrix of the whole population.


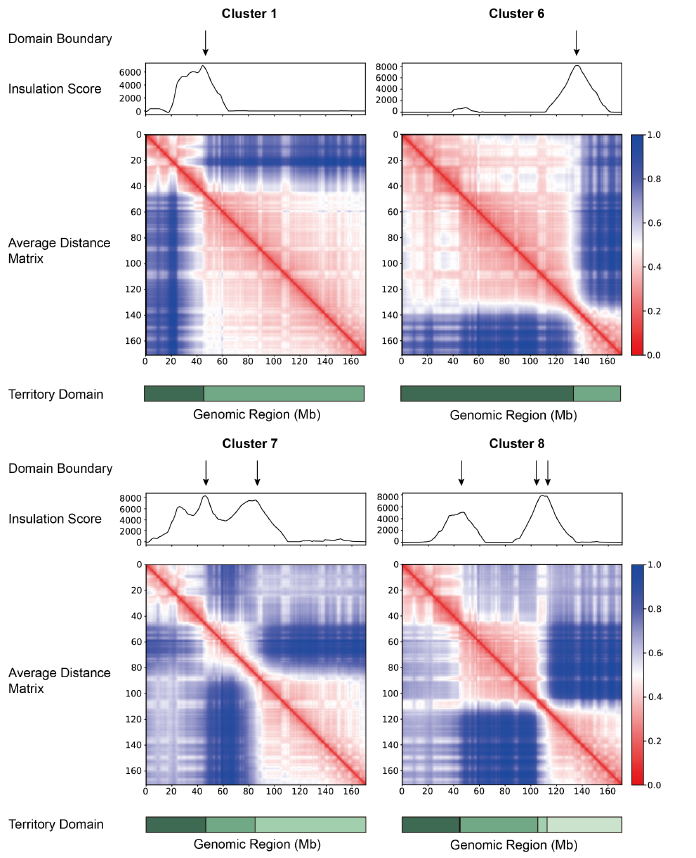


**Fig. R7: Domain boundary analysis for selected clusters from GM12878 Chr6** (From top to the bottom) The first row shows the insulation score calculated from the average distance matrix. The second row indicates the territory domains separated by the domain boundaries. shows the domain boundaries identified. The second rowWe find that the domain boundaries are actually among the major peaks found in the insulation score profile.


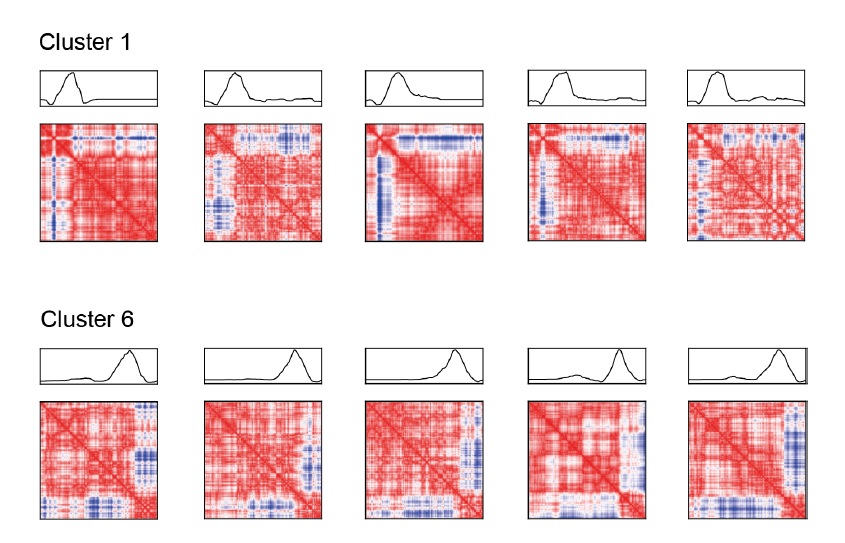


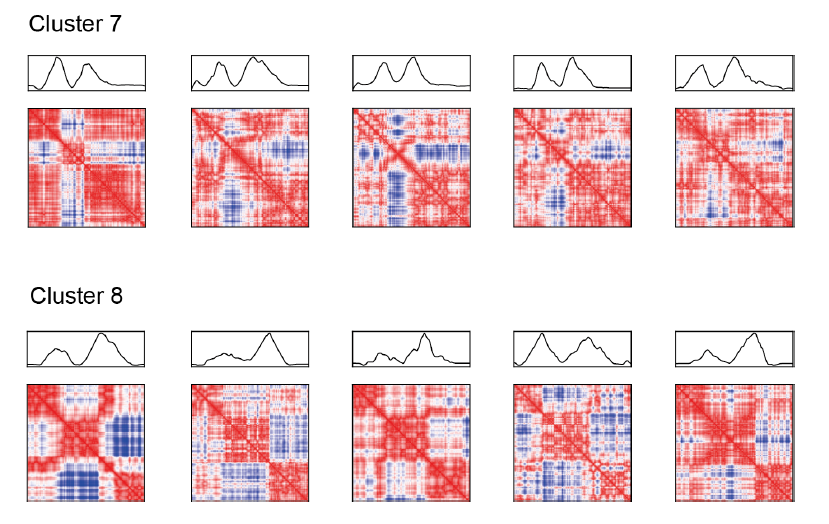


**Fig. R8 Selected single-cell examples for each cluster found in GM12878 Chr6** We find that single-cell distance matrices in each cluster show high similarity in large-scale conformation and territory domains which can be detected by the insulation score profile (shown on top of each distance matrix), although variability and flexibly distributed conformations are observed at small scale.

f. Why not applying the method directly to experimental data and check the direct consistency with data-driven structures at the same resolution and with same number of inputs ?

The available whole genome imaging data contains a relatively small number of cells and relatively low resolution. For example, the DNA MERFISH data provided by Su et al [4] imaged 3,668 whole-genome structures at 3Mb resolution. We showed that at relatively low resolution with a limited number of structures, only a subset of clusters—specifically those with the highest occupancy—can be reliably detected (see also reviewer #2 minor concern #1). We do provide results for direct clustering of experimental datasets in **Fig. S13**, which detected three clusters. However, our work emphasizes that in such cases genome structure models from Hi-C data allow detection of conformational clusters that can then be used to identify corresponding structures from low resolution imaging. This highlights the benefits of our approach using genome structure models from Hi-C data. Due to the rapid developments in multiplexed FISH imaging, we believe that there will be many more experimental data sets available in near future that contain higher resolution structures with larger sampling sizes.

3. It misses discussions on the limits of the method and of the approach. For example, fair discussions about the comparisons with experimental (MERFISH, scHIC) are missing. Comparisons with papers by other authors doing similar things would be useful to highlight the originality of the present work.

Following the reviewer’s suggestion we now provide a better description about the limits of our approach at lower structure resolution and population sizes on the clustering results in the results section. (Page 9, lines 186 to 206.) We also mention this limitation in the discussion section (page 25, lines 585 to 586): “At limited coverage and population size, clustering chromosome structures from tracing experiments is challenging.”

We have discussed other related work in the background section. We now added a sentence to highlight better the originality of our approach. “However, to our knowledge no method exists to study at higher resolution chromosome conformational states within the context of the subnuclear environment.” (Page 3, lines 58 to 59.)

The originality of our approach is further described in the background section (page 5, lines 95 to 100): “Unlike other unsupervised clustering methods [1,9,10], our approach does not coerce all the conformations into a defined set of clusters. Instead, it rather detects only those morphologies that are substantiated by a sufficient number of similar structures at a given sample size. Furthermore, we examine significantly larger chromosomes from the entire human genome and concentrate our analysis on the connection between chromosome morphology and nuclear topography.“

Minor concerns:

1. The term 'nuclear topography' is interesting but unusual in the field. It might be clearly defined when introduced for the first time.

We now add a definition of the term “nuclear topography” as the “subnuclear location with respect to nuclear bodies, radial position and nuclear compartments”. (Page 4, lines 64 to 65 in the revised manuscript.)

2. L95-96 "This is because certain …": unclear, please reformulate.

We now rephrase the sentence as “This is because certain functionally unrelated regions can show substantial variability in their relative positions, potentially obscuring the detection of functionally relevant structural similarities among other chromosomal regions within a subpopulation of structures.” (Page 5, lines 86 to 89 in the revised manuscript.)

3. The end of the introduction is a very long, very detailed summary of the paper which is redundant with the abstract and the conclusion part. I would suggest to just enumerate what authors will describe in the rest of the paper without detailing the results.

Following the reviewer’s suggestion, we shortened the end of the discussion section and the abstract. We see abstract and introduction as independent entities. So, we prefer to have a high-level discussion of the results at the end of the introduction section.

4. It would be interesting for visual comparison to show the experimental Hi-C map at 200kbp resolution for the different chromosomes and cell types investigated in the paper.

Following the reviewer’s suggestion we now add new panels **S8DI** in supplementary figure **Fig. S8,** comparing the input ensemble Hi-C matrix (lower diagonal) and the reconstructed matrix from the output model (upper diagonal) for each chromosome.

5. It should be stated in the figure caption and in the text that the cell type mostly investigated is GM12878.

We thank the reviewer for pointing this out. We now refer to GM12878 cells in Figure caption 2, 4, 5, 6, 7 and in the text.

6. In Table S1 may be nice to have the proportion of the structures that are clustered.

Following the reviewer’s suggestion we now added a new column in **Table S1** showing the

numbers of clustered structures for all chromosomes.

7. Fig.2D: in the caption, it is unclear what are the specific genomic regions I, II ,II below the RadRation & RGRatio profiles.

We assume that the reviewer refers to **Fig. 5A**, as **Fig. 2D** does not show RadRatio and RgRatio profiles nor regions **I**, **II**, **III** and **IV**. We now better clarify the definitions of regions **I**-**IV** in the figure caption of figure **5A**. Previously these regions were only defined in panel B and not A. We apologize for this mistake. We also increased the fonts indicating regions **I** to **IV** as a visual aid. We also added a red block indicating location of region **II** in RadRatio profile of cluster 4, which was missing.

8. Fig.2F: it seems that the decay of the P(s) curve is slower than the expected exponent ( ~ -1), why ?

The expected P(s) exponent of (~-1) in Lieberman-Aiden et al [11] and Rao et al [5] is calculated from the ensemble average over all chromosomes in GM12878, however exponents can vary depending on individual chromosomes, and in particular for long-range components in different conformational clusters. At shorter sequence distances (<5Mb), the decay rate of all chromosomes is similar to -1 (**Fig. R6**).

9. The term 'chromatin fiber compaction' could be confusing as it may refer to the local compaction of the fiber (nucleosome positioning, density) or to the larger-scale condensation of the fiber (which is the meaning used in the manuscript). It may be better to use another term or to clearly define it.

We thank the reviewer for this suggestion. As requested we changed “chromatin fiber compaction” to “chromatin fiber condensation”.

10. L242-244 "Overall, the relative …": unclear, please reformulate.

We removed this sentence.

11. Fig.S9: the caption should be more detailed to guide the reader. Maybe I misunderstood, but it seems that the 'null', randomized model is obtained by fully randomizing all the contacts, a better null model would be to randomize contact per sub-diagonal to conserve the overall polymeric structure.

I am sorry for the confusion. In **Fig. S9C** of the original manuscript (now **Additional file 1: Fig. S14C**), we indeed randomly reshuffled the sub-diagonal part of the matrix, while keeping the diagonal constant. This keeps the matrix symmetric and the total number of contacts unchanged.

12. L298-342, there are many 'squares' in the text.

We fixed all these issues.

13. Fig.5: in absolute values, the Radratio (also the Rgratio) are very small and may correspond to relative changes of 10-20 % compared to the average. Can the authors comment? Do we expect an anticorrelation between Radratio and Rgratio ?

These values are based on ensemble averages, which typically are very stable and thus 10-20% difference is a quite dramatic change. These differences are highly significant with very low p-values. Also, the total population average includes all conformations, including the target state conformations.

14. Fig. 5D: regarding the previous point, the scheme seems a little bit exaggerated, moreover in Fig.5, the speckle aspects are not mentioned.

We remove the speckle illustration in **Fig. 5D** to be consistent with the text.

15. Fig.6A: there is no blue line (while it is present in panel C)

We apologize for the confusion. In panel **Fig. 6A** we are only showing the interchromosomal proximity profile (IPP) for the full ensemble of structures. That’s why we show the red curve only. In panel **Fig. 6C**, we compare the IPP profiles calculated from structures in clusters (blue curve) with the IPP profile from the full ensemble (red curve). We now better explain this point in figure caption.

16. Fig.7C: how speckle distances are inferred from MERFISH?

The DNA-MERFISH dataset from Su et al [4] contains measured speckle distances and we directly use this data in our analysis. The details are included in the imaging assessment part in the method.

17. L452-453 "These observations …": this is an overstatement: authors show correlation but not a causal relation.

We reformulate the sentence as “These observations indicate that variations in chromosome conformations correlate with gene transcription levels, possibly as a result of variations in the specific nuclear locations of genes in different conformations. However, further experimental evidence is required to establish a definitive causal link between chromosome conformation and gene expression.” (Page 21, lines 489 to 492 in the revised version of the manuscript.)

18. Fig.8C: what are 'left 2' or 'left 4' loci ?

We now clarify this point in the revised manuscript. Since each genomic window (ie., bead) in our model represents a 200kb region, we replace left/right 2 with +/- 400kb in **Fig. 8C** and its captions.

19. Tables in the main text may be relocated in the Supplementary Material.

As suggested by the reviewer we moved **Table 1, 2 and 3** to the **Additional file 1** as **Table S2, S3 and S4**.

20. Both the conclusion and discussion parts contain very similar summaries of the work, this is very repetitive, one is enough (see also remark on the end of the introduction).

As suggested by the reviewer we cut down the conclusions to remove redundancies.

21. L859: refs [17,51] are repeated twice.

Thank you for pointing this out, we now fixed this issue.

22. L893: "multiples of 50 by bilinear interpolation": unclear.

For technical reasons, each input matrix is resized so that the voxel number per row is divisible by , because our autoencoder contains downsampling and 50 = 5×5×2 upsampling layers, two of which are of size (5, 5) and the other has size (2, 2). Otherwise the output matrices of the autoencoder will not match the size of the input matrices. For instance, if voxel numbers per row would be 102 the resizing would lead to a bin/voxel number of 100. We clarified this aspect now better in the Methods section and shifted the text to the Convolutional Autoencoder section of Methods. (Page 44, lines 926 to 931.)

23. About the different layers used for the auto-encoder: is it standard? if yes, please add references, if not what is the rationale behind these choices?

We use an autoencoder as described in AlexNet [12], which is a representative convolutional neural network framework and is frequently cited as reference for autoencoder structures. It contains the same layers such as 2D convolutional layers and 2D max pooling layers and the same activation functions such as ReLU and sigmoid used in our model. We now cite the paper in the beginning of the autoencoder description in the Methods section. (Page 44, lines 917 to 919.)

24. L1009: "can be we are able to find": typo

Thank you, we fixed the typo.

25. L1061: "is structure": typo

Thank you, we fixed the typo.

26. L1156: "The peaks of the insulation…": what is the algorithm use for peak detection?

For the peak detection, we use detecta https://github.com/demotu/detecta to calculate the positions of local maxima. We added the reference in the Methods section. We now better describe the insulation score in the Methods section and also show an example of the insulation score profiles for cluster 7 and cluster 8 in the **Fig. R7** and other clusters in **Fig. R8**. (Pages 56 to 57, lines 1178 to 1200.)

27. L1200-1214: the CIN, SAF and Markov clustering are never mentioned in the main text. It is unclear why there are materials and methods subsections for that.

We apologize for the confusion. We now rewrote this part in the Methods section under the paragraph: “Prediction of speckle locations in single cells“, which combines the use of CIN and Markov clustering as a tool to predict speckle locations in single cells. We follow our procedure as outlined in Yildirim et al [13]. Using these speckle locations we calculate several structural features, including speckle distance (SpD), speckle TSA-seq and speckle association frequency (SAF), as for instance shown and discussed in **Fig. 8**. (Page 59, lines 1245 to 1255.)

28. L1303: "contact range 2": unclear.

We added a sentence to clarify “contact when two beads are within the range of twice the bead radius”. (Pages 63 to 64, lines 1344 to 1345.)

29. L1308-1311: the rationale behind the specific choice is unclear (eg, theshholds 5 and -1)

The threshold 5 and -1 are around the top 0.1% entries and the bottom 0.1% entries in the difference matrices for all clusters. We select the extreme entries which represent both low-contact frequency entries and high-contact frequency entries to construct masks for these clusters used for classification of the experimental sci-HiC matrices [8].

**References**

1. Cheng RR, Contessoto VG, Lieberman Aiden E, Wolynes PG, Di Pierro M, Onuchic JN.

Exploring chromosomal structural heterogeneity across multiple cell lines. Elife. 2020;9:e60312.

2. Barbieri M, Chotalia M, Fraser J, Lavitas L-M, Dostie J, Pombo A, et al. Complexity of

chromatin folding is captured by the strings and binders switch model. Proc Natl Acad Sci USA.

2012;109:16173–8.

3. Patta I, Zand M, Lee L, Mishra S, Bortnick A, Lu H, et al. Nuclear morphology is shaped by

loop-extrusion programs. Nature. 2024;627:196–203.

4. Su J-H, Zheng P, Kinrot SS, Bintu B, Zhuang X. Genome-Scale Imaging of the 3D

Organization and Transcriptional Activity of Chromatin. Cell. 2020;182:1641-1659.e26.

5. Rao SSP, Huntley MH, Durand NC, Stamenova EK, Bochkov ID, Robinson JT, et al. A 3D

map of the human genome at kilobase resolution reveals principles of chromatin looping. Cell.

2014;159:1665–80.

6. Liu S, Zheng P, Wang CY, Jia BB, Zemke NR, Ren B, et al. Cell-type-specific 3D-genome

organization and transcription regulation in the brain. bioRxiv. 2023;2023.12.04.570024.

7. Boninsegna L, Yildirim A, Polles G, Zhan Y, Quinodoz SA, Finn EH, et al. Integrative genome

modeling platform reveals essentiality of rare contact events in 3D genome organizations. Nat

Methods. 2022;19:938–49.

8. Ramani V, Deng X, Qiu R, Gunderson KL, Steemers FJ, Disteche CM, et al. Massively

multiplex single-cell Hi-C. Nat Methods. 2017;14:263–6.

9. Sawh AN, Shafer MER, Su J-H, Zhuang X, Wang S, Mango SE. Lamina-Dependent

Stretching and Unconventional Chromosome Compartments in Early C. elegans Embryos. Mol

Cell. 2020;78:96-111.e6.

10. Götz M, Messina O, Espinola S, Fiche J-B, Nollmann M. Multiple parameters shape the 3D

chromatin structure of single nuclei at the doc locus in Drosophila. Nat Commun. 2022;13:5375.

11. Lieberman-Aiden E, Van Berkum NL, Williams L, Imakaev M, Ragoczy T, Telling A, et al.

Comprehensive Mapping of Long-Range Interactions Reveals Folding Principles of the Human

Genome. Science. 2009;326:289–93.

12. Krizhevsky A, Sutskever I, Hinton GE. ImageNet classification with deep convolutional

neural networks. Commun ACM. 2017;60:84–90.

13. Yildirim A, Hua N, Boninsegna L, Zhan Y, Polles G, Gong K, et al. Evaluating the role of the nuclear microenvironment in gene function by population-based modeling. Nat Struct Mol Biol.2023;30:1193–206.

**2nd round**

**Reviewer 2**

The authors has addressed all my concerns in the revised manuscript.

**Reviewer 3**

The authors have addressed or satisfyingly discussed most of my previous concerns.

Few minor suggestions/comments regarding the new add-ons:
- Line 46-48: in Drosophila, Szabo et al (Science Adv 2018) showed that TADs may exist at the single-cell level
- I still find the summary at end of introduction too long and redundant with the actual conclusion of the paper.
- In the negative control case (homopolymer, Page 9-10), several clusters are still found by the algorithm. Could it be used to fix the meta-parameters of the clustering method to avoid finding several clusters ?
- alpha, beta, gamma appear as squares in the PDF of the manuscript (Page 14-16).
- citation/discussion of the preprint Messina et al (biorxiv 2024.09.18.613689) may be interesting.
- Page 15: it would be nice to indicate the domain i on Fig.4A.
